# Disruption of the KLHL37–N-Myc complex restores N-Myc degradation and arrests neuroblastoma growth in mouse models

**DOI:** 10.1172/JCI176655

**Published:** 2025-06-10

**Authors:** Senfeng Xiang, Pengfei Chen, Xiaoxian Shi, Hanqi Cai, Zihan Shen, Luyang Liu, Aixiao Xu, Jianhua Zhang, Xingya Zhang, Shaowei Bing, Jinhu Wang, Xuejing Shao, Ji Cao, Bo Yang, Qiaojun He, Meidan Ying

**Affiliations:** 1 Institute of Pharmacology and Toxicology, Zhejiang Province Key Laboratory of Anti-Cancer Drug Research, College of Pharmaceutical Sciences, Zhejiang University, Hangzhou, China.; 2 Nanhu Brain-Computer Interface Institute, Hangzhou, China.; 3 Children’s Hospital, Zhejiang University School of Medicine, National Clinical Research Center for Child Health, Hangzhou, China.; 4 Engineering Research Center of Innovative Anticancer Drugs, Ministry of Education, Hangzhou, China.; 5 School of Medicine, Hangzhou City University, Hangzhou, China.

**Keywords:** Oncology, Therapeutics, Cancer, Drug therapy, Ubiquitin-proteosome system

## Abstract

The N-Myc gene *MYCN* amplification accounts for the most common genetic aberration in neuroblastoma and strongly predicts the aggressive progression and poor clinical prognosis. However, clinically effective therapies that directly target N-Myc activity are limited. N-Myc is a transcription factor, and its stability is tightly controlled by ubiquitination-dependent proteasomal degradation. Here, we discovered that Kelch-like protein 37 (KLHL37) played a crucial role in enhancing the protein stability of N-Myc in neuroblastoma. KLHL37 directly interacted with N-Myc to disrupt N-Myc–FBXW7 interaction, thereby stabilizing N-Myc and enabling tumor progression. Suppressing KLHL37 effectively induced the degradation of N-Myc and had a profound inhibitory effect on the growth of *MYCN*-amplified neuroblastoma. Notably, we identified RTA-408 as an inhibitor of KLHL37 to disrupt the KLHL37–N-Myc complex, promoting the degradation of N-Myc and suppressing neuroblastoma in vivo and in vitro. Moreover, we elucidated the therapeutic potential of RTA-408 for neuroblastoma using patient-derived neuroblastoma cell and patient-derived xenograft tumor models. RTA408’s antitumor effects may not occur exclusively via KLHL37, and specific KLHL37 inhibitors are expected to be developed in the future. These findings not only uncover the biological function of KLHL37 in regulating N-Myc stability, but also indicate that KLHL37 inhibition is a promising therapeutic regimen for neuroblastoma, especially in patients with *MYCN*-amplified tumors.

## Abstract 2

KLHL37 counteracts the FBXW7-induced degradation of N-Myc and enables the progression of neuroblastoma, indicating that inhibiting KLHL37 with RTA-408 could be a potential therapeutic strategy.

## Introduction

Neuroblastoma, derived from the peripheral sympathetic nervous system, is the most common extracranial solid tumor in children and accounts for 15% of pediatric tumor–associated deaths (1). Despite the standard treatments of surgical excision, radiotherapy, and chemotherapy, the prognosis for patients with high-risk neuroblastoma remains poor, with a 5-year survival rate of less than 50% (2–5). N-Myc gene (*MYCN*) amplification occurs in approximately 20% of patients with neuroblastoma, and all patients with *MYCN*-amplified tumors are classified as high risk with an unfavorable outcome (5–7).

Amplification of the *MYCN* oncogene leads to high expression levels and functional activation of N-Myc protein, which is a well-known oncogenic transcription factor. N-Myc is an important regulator that activates genes responsible for self-renewal, proliferation, and metastasis, while repressing genes involved in cell-cycle arrest, differentiation, and apoptosis (8–11). Transgenic overexpression of the N-Myc gene in mice and zebrafish induces tumors with characteristics similar to those of human neuroblastoma (12), whereas inhibition of N-Myc expression significantly arrests the growth of neuroblastoma (13). These insights suggest that efficient inhibition of N-Myc is supposed to achieve therapeutic benefits for patients with neuroblastoma. To date, many efforts have been made to develop targeted strategies against N-Myc, with inhibition targets including CDK7 (9), BRD4 (14), Aurora-A (15, 16), PLK1 (17), and ALDH18A1 (18). However, these strategies remain clinically unavailable in the treatment of neuroblastoma. Therefore, it remains urgent to elucidate the critical regulatory mechanisms of N-Myc and develop alternative efficient strategies that target N-Myc function.

The Kelch-like gene family (KLHL) proteins comprise 42 members that function as substrate adaptors in the Cullin 3–scaffold complex and regulate the ubiquitination process of substrate proteins, which have functions in the pathogenesis of various human diseases including cancer (19) Kelch-like protein 37 (KLHL37), also known as ectodermal-neural cortex 1 (ENC1), was first identified as being primarily expressed in the nervous system, where it participates in neuronal differentiation (20, 21), and studies have revealed that KLHL37 is upregulated in several tumors including colorectal carcinoma (22, 23) and breast cancer (24), and is associated with a poor patient prognosis (25, 26). Considering that both KLHL37 and N-Myc are associated with the nervous system, we wondered whether KLHL37 is associated with N-Myc and involved in the process of neuroblastoma.

In this study, we demonstrated that KLHL37 competitively interacted with N-Myc and released N-Myc from E3-mediated proteasomal degradation, leading to enhanced stabilization and aberrant activation of N-Myc, ultimately contributing to the malignant progression of neuroblastoma. Knockdown of KLHL37 or pharmacological inhibition of KLHL37 with the small molecule RTA-408 both markedly promoted the degradation of N-Myc and arrested neuroblastoma growth. These findings reveal the KLHL37/N-Myc axis as an essential oncogenic signaling axis underlying the progression of neuroblastoma. Moreover, we propose KLHL37 inhibitors as a selective therapeutic strategy for patients with *MYCN*-amplified neuroblastoma.

## Results

### KLHL37 coordinates with N-Myc to promote neuroblastoma progression.

**Figure 1:**
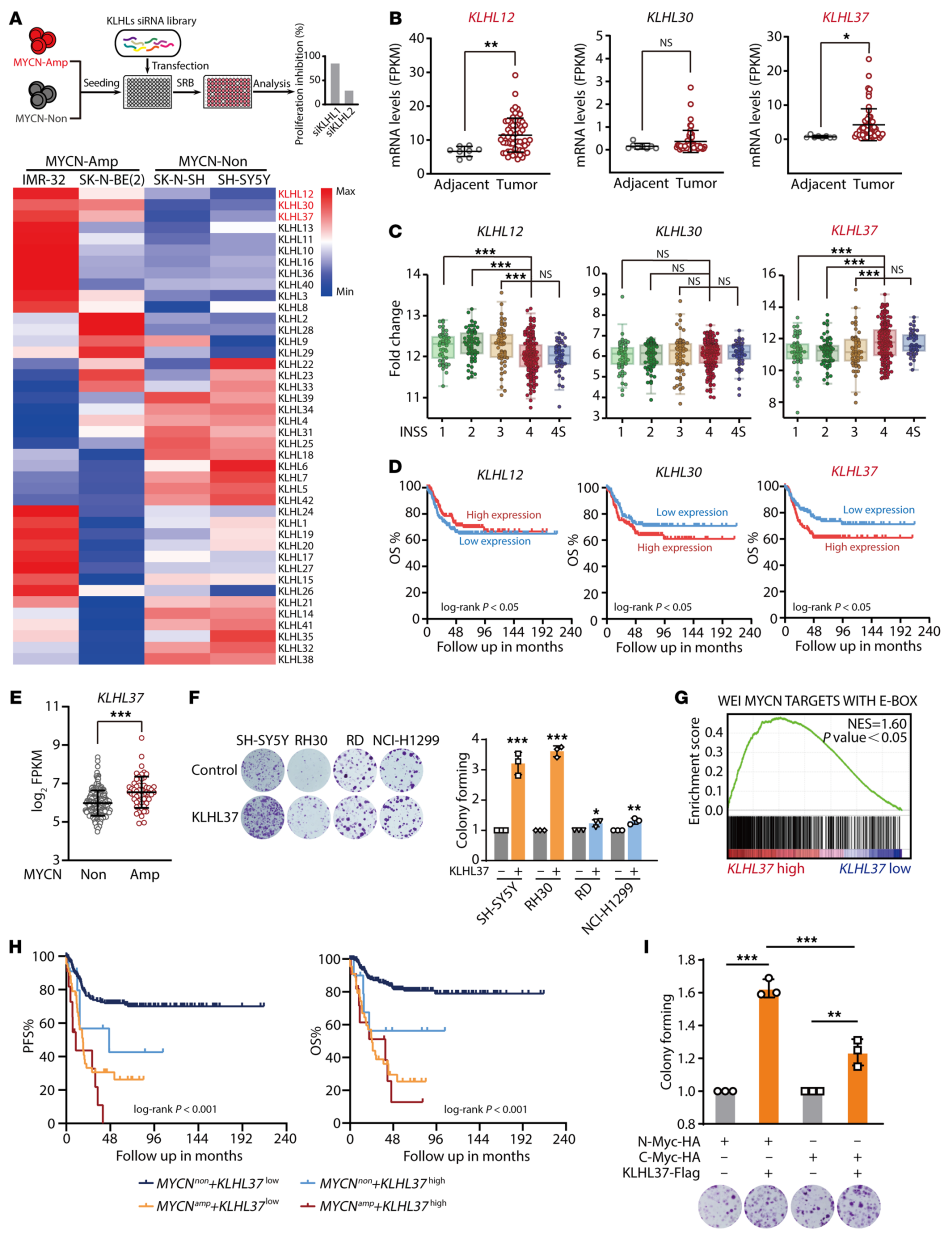
KLHL37 coordinates with N-Myc to promote neuroblastoma progression. (**A**) *MYCN*-amplified (MYCN-Amp) or non-*MYCN*-amplified (MYCN-Non) cells were transfected for 72 hours with a siRNA library specifically targeting KLHL proteins. The effect of the siRNAs on cell proliferation was examined and visually presented as a heatmap. SRB, sulforhodamine B. (**B**) *KLHL37* expression in tumor (*n* = 59) or adjacent (*n* = 8) tissue was analyzed. FPKM, fragments per kilobase of transcript per million mapped reads. (**C**) The correlation between expression levels of *KLHL12*, *KLHL30*, and *KLHL37* and clinical progression staging of patients with neuroblastoma was analyzed in comparison with the GEO GSE120572 cohort. The numbers of patients with stage 1, *n* = 54; stage 2, *n* = 67; stage 3, *n* = 58; stage 4, *n* = 168; stage 4S, *n* = 46, respectively. INSS, International Neuroblastoma Staging System. (**D**) Kaplan-Meier survival curves for OS of neuroblastoma patients with high (*n* =138) or low (*n* = 138) *KLHL12*, *KLHL30*, and *KLHL37* expression levels. (**E**) Analysis of *KLHL37* expression in patients with *MYCN*-amplified (*n* = 55) or non-*MYCN*-amplified (*n* = 222) neuroblastoma. Data were derived from the GEO GSE85047 cohort. (**F**) Effect of KLHL37-Flag overexpression on the clonogenic capacity of tumor cells with high N-Myc expression (SH-SY5Y and RH30) or low N-Myc expression (RD and NCI-H1299). (**G**) GSEA of the correlation of *MYCN* target gene signature enrichment with high *KLHL37* expression in the GEO GSE85047 cohort. NES, normalized enrichment score; WEI, gene set. (**H**) Analysis of the impact of high *KLHL37* expression and *MYCN* amplification on the progression-free survival (PFS) or OS of the patients with neuroblastoma based on the GEO GSE85047 cohort. The numbers of patients in the 4 groups are 211, 11, 44, and 11, respectively. (**I**) Effect of KLHL37 overexpression on the colony-forming ability of NIH-3T3 cells exogenously overexpressing N-Myc or C-Myc. *P < 0.05, **P < 0.01 and ***P < 0.001, by unpaired, 2-tailed Student’s *t* test (**B**, **E**, and **F**) and 1-way ANOVA (**C** and **I**). Data represent the mean ± SEM, except in **F** and **I** where data represent the mean ± SD.

To systematically uncover the relevance of KLHL family proteins to N-Myc, we first assessed the effect of the KLHL family on the proliferation of *MYCN*-amplified and non-*MYCN*-amplified neuroblastoma cells using an siRNA library targeting KLHLs. Our results revealed that selective inhibition of *MYCN*-amplified cells, but not non-*MYCN*-amplified cells, occurred upon silencing of KLHL12, KLHL30, and KLHL37 (Figure 1A). These observations suggest that these 3 KLHLs may have potential biological roles in neuroblastoma and may be associated with N-Myc. Moreover, we found that *KLHL12* and *KLHL37* had higher expression levels in tumor tissues compared with levels in adjacent tissues, whereas *KLHL30* did not (Figure 1B). According to the transcriptomics data from patients with neuroblastoma (27), *KLHL37* was observed to be significantly upregulated in patients with stage 4 disease, which is associated with a poor prognosis. By contrast, *KLHL12* was expressed a low levels in patients with stage 4 disease, and *KLHL30* did not exhibit significant changes across stages 1–4 (Figure 1C). Subsequent Kaplan-Meier analysis revealed that high expression of *KLHL37*, rather than *KLHL12* or *KLHL30*, was significantly associated with poor overall survival (OS) of patients with neuroblastoma (Figure 1D). These findings suggest that KLHL37 may play a crucial role in the progression of neuroblastoma.

Encouraged by the selective inhibitory effect of siKLHL37 on *MYCN*-amplified cells, we hypothesized that KLHL37 may play a regulatory role in N-Myc. By analyzing GSE85047 cohort (28), we found that *KLHL37* was significantly upregulated in patients with *MYCN-*amplified neuroblastoma (Figure 1E). Meanwhile, we observed that when KLHL37 was overexpressed in different cancer cells, it promoted the clonogenic ability of these cells, and this enhancement was particularly pronounced in cells with an N-Myc expression background, such as neuroblastoma SH-SY5Y and rhabdomyosarcoma RH30 cells (Figure 1F and Supplemental Figure 1A; supplemental material available online with this article; https://doi.org/10.1172/JCI176655DS1). Further gene set enrichment analysis (GSEA) revealed that patients with neuroblastoma who had high expression of *KLHL37* showed activation of N-Myc target genes (Figure 1G). Notably, high expression of *KLHL37* in patients with *MYCN*-amplified neuroblastoma was associated with a worse prognosis compared with those with low *KLHL37* expression (Figure 1H). These results indicate the potential correlation between N-Myc and KLHL37.

In addition, we observed that high *KLHL37* expression was also associated with poor outcomes for patients with non-*MYCN*-amplified neuroblastoma. Given that N-Myc is dominant in patients with *MYCN*-amplified neuroblastoma, whereas its highly homologous protein C-Myc plays a role in patients with non-*MYCN*-amplified disease (29–31), as also indicated by their specific expression and CRISPR sensitivity (Supplemental Figure 1, B and C), we suspected that KLHL37 might also have a regulatory effect on C-Myc that would explain the poor prognosis of patients with non-*MYCN*-amplified disease. Consistently, we found that KLHL37 could enhance the clonogenic ability of cells expressing either N-Myc or C-Myc, with a more pronounced effect observed in cells expressing N-Myc (Figure 1I). Taken together, our findings suggest that KLHL37 might play an important role in the malignant progression of neuroblastoma, especially in patients with *MYCN*-amplified disease.

### Targeting KLHL37 inhibits the progression of MYCN-amplified neuroblastoma.

**Figure 2:**
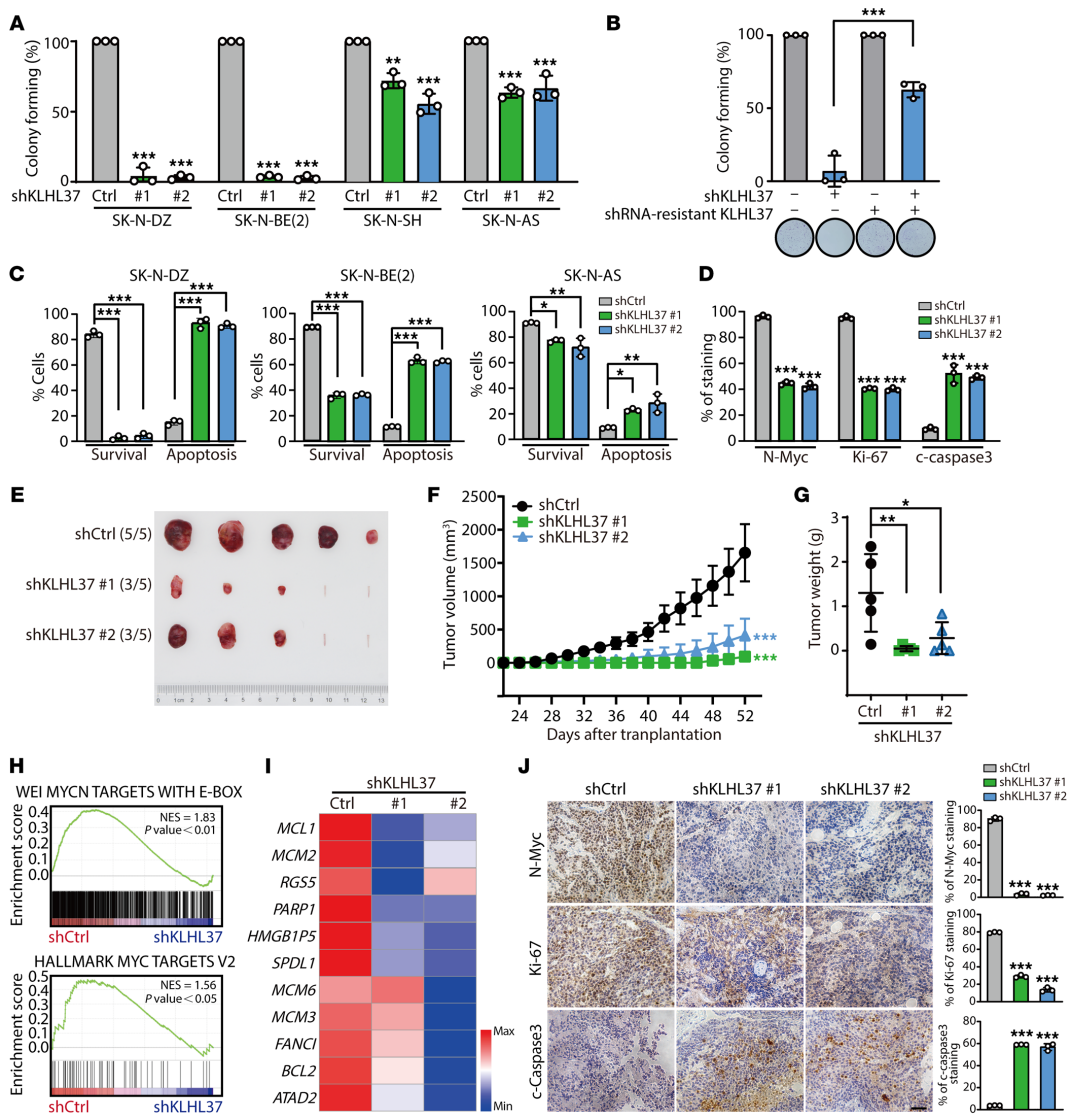
Targeting KLHL37 inhibits the progression of *MYCN*-amplified neuroblastoma. (**A**) Colony formation assay of neuroblastoma cells transduced with lentivirus-shKLHL37 (nos. 1 and 2). The colony-forming rate of shKLHL37 groups was calculated. (**B**) Overexpression of KLHL37-synonymous mutants, which were resistant to the knockdown effect of shKLHL37 no. 2, reversed the suppression of SK-N-DZ cell colony formation due to KLHL37 knockdown. (**C**) Effect of KLHL37 knockdown on apoptosis of SK-N-DZ, SK-N-BE(2), and SK-N-AS cells was determined by flow cytometry when cells were infected with shKLHL37 lentivirus for 5 days. (**D**) Analysis of histological staining for N-Myc and markers of proliferation and apoptosis in SK-N-DZ cells. (**E**) Images of SK-N-BE(2) xenograft tumors were captured at the end of the experiment. (**F**) Tumor volumes were measured every 2 days, and tumor growth curves are shown as the mean ± SEM. (**G**) Tumor weights of SK-N-BE(2) xenografts. (**H**) GSEA plots show the correlation between the enrichment of *MYCN* downstream genes and KLHL37 depletion. (**I**) Heatmap of changes in expression of survival-related genes in the KLHL37 depletion group. min, minimum; max, maximum. (**J**) Histological staining for N-Myc protein and markers of proliferation and apoptosis in SK-N-BE(2) xenograft tumors. Scale bar: 100 μm. *P < 0.05, **P < 0.01, and ***P < 0.001, by 1-way ANOVA (**A**–**D**, **G**, and **I**), unpaired, 2-tailed Student’s *t* test (**B**) and 2-way ANOVA (**F**) Data represent the mean ± SD in **A**, **B**, **C**, **D**, **G**, and **J**.

We next investigated whether targeting KLHL37 could inhibit the progression of *MYCN*-amplified neuroblastoma. The results showed that depletion of KLHL37 via an shRNA led to dramatic inhibition of the clonogenic ability in SK-N-DZ and SK-N-BE(2) cells, which harbor amplification of the *MYCN* gene, whereas a modest inhibitory effect was observed in non-*MYCN*-amplified SK-N-SH and SK-N-AS cells (Figure 2A and Supplemental Figure 2A). Next, 2 shRNA-resistant KLHL37 constructs based on shRNA 1 and 2 sequences were introduced, and they obviously rescued the inhibition effect of shKLHL37 on colony formation (Figure 2B and Supplemental Figure 2, B and C). Besides, KLHL37 knockdown induced significant apoptosis of *MYCN*-amplified neuroblastoma cells (Figure 2C and Supplemental Figure 2D). Further histological staining results indicated that KLHL37 knockdown significantly reduced N-Myc protein levels, inhibited cell proliferation, and induced cell apoptosis (Figure 2D and Supplemental Figure 2E). These findings indicate that targeting KLHL37 could inhibit the colony-forming ability and induce the death of *MYCN*-amplified neuroblastoma cells.

We further assessed the in vivo tumorigenicity of *MYCN*-amplified neuroblastoma cells transfected with shKLHL37. The results demonstrated that KLHL37 depletion obviously reduced the rate of tumor formation (100% in control vs. 60% in both shKLHL37 nos. 1 and 2) (Figure 2E and Supplemental Figure 3A). The average volume of tumors in the control groups increased continuously, reaching an average tumor volume at the end of the experiment of 1,652.8 mm^3^ compared with 92.7 mm^3^ and 411.7 mm^3^ in shKLHL37 no. 1 and no. 2 groups (Figure 2F). Moreover, shKLHL37 significantly reduced tumor weights (Figure 2G), and showed notable therapeutic activity, as indicated by a relative tumor volume (RTV) treatment/control (T/C) value of 6.31% and 25.26% (determined by RTV_Treatment_/RTV_Control_ × 100%) (Supplemental Table 1). Additionally, no apparent body weight loss was observed in the shKLHL37 groups (Supplemental Figure 3B). Overall, these findings demonstrate that targeting KLHL37 exerted a remarkable inhibitory effect on the growth of *MYCN*-amplified xenograft tumors. Further RNA-Seq analysis revealed that N-Myc downstream and prosurvival genes were inhibited in the shKLHL37 groups (Figure 2, H and I). Additionally, immunohistochemical analysis showed that KLHL37 knockdown depleted N-Myc expression concomitant with obvious intratumoral proliferation inhibition and apoptosis activation as quantified by N-Myc, Ki67 and cleaved caspase 3 (c–caspase 3) staining (Figure 2J). In summary, these results show that KLHL37 might be a promising therapeutic target for *MYCN*-amplified neuroblastoma.

### KLHL37 stabilizes N-Myc protein by interfering with its ubiquitination process.

**Figure 3:**
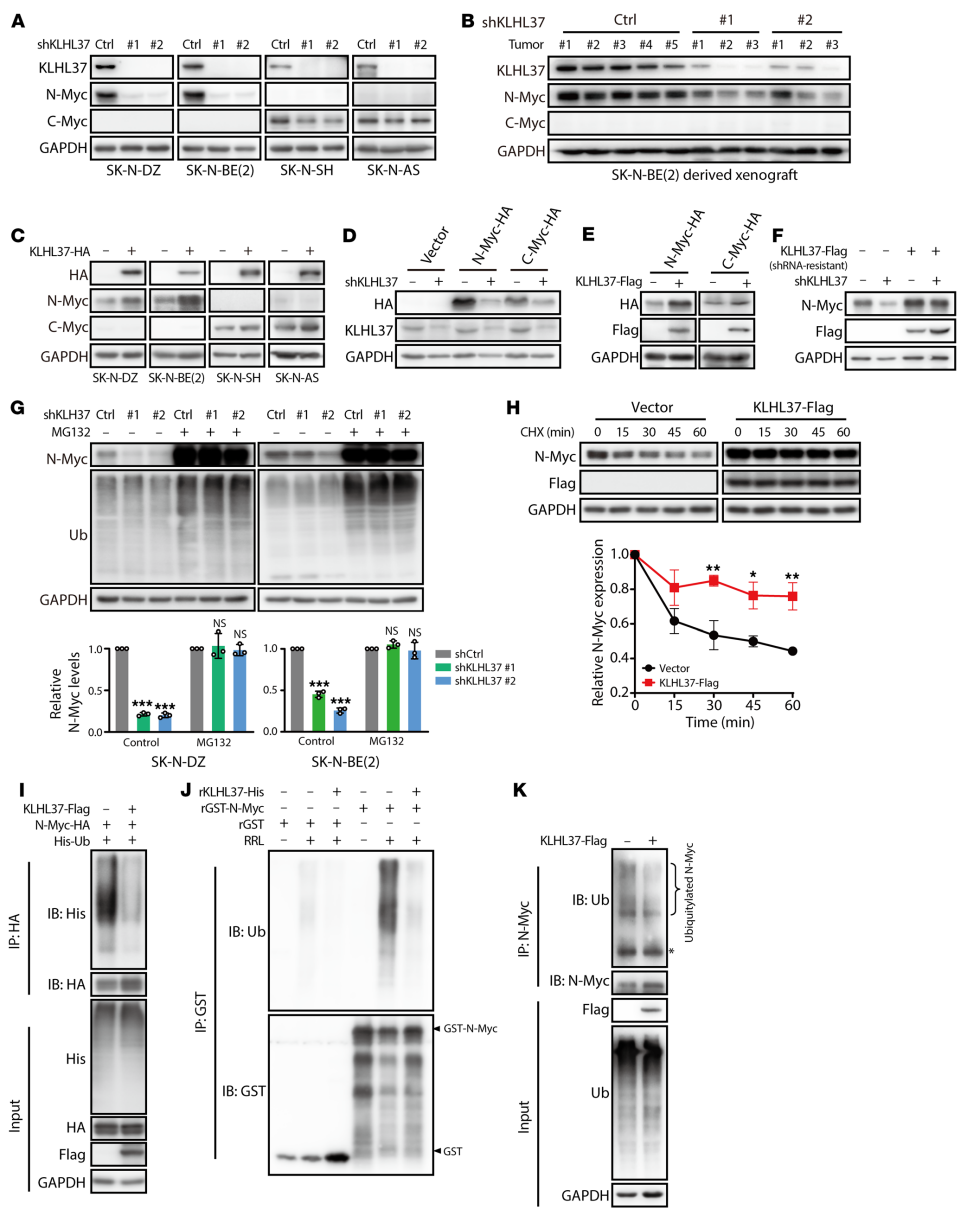
KLHL37 stabilizes N-Myc protein by interfering with its ubiquitination process. (**A**) Effect of KLHL37 knockdown on endogenous N-Myc protein expression. (**B**) KLHL37 depletion efficiency and N-Myc and C-Myc protein expression changes in SK-N-BE(2)-derived xenograft tumors were detected using immunoblotting. (**C**) Effect of KLHL37 overexpression on endogenous N-Myc protein levels in neuroblastoma cells. (**D**) Effect of KLHL37 knockdown on exogenously expressed N-Myc and C-Myc in RD cells. (**E**) Effect of KLHL37 overexpression on exogenous N-Myc protein levels in NIH-3T3 cells. (**F**) Overexpression of KLHL37-synonymous mutants, which were resistant to the knockdown effect of shKLHL37 no. 2, rescued the downregulation of N-Myc protein levels due to KLHL37 knockdown. (**G**) Effect of the proteasome inhibitor MG132 on the decline in N-Myc protein expression induced by knockdown of KLHL37. Cells were infected with lentivirus-shKLHL37 or control shRNA for 3 day and then treated with MG132 (10 μM) for 8 hours. (**H**) Effect of KLHL37 overexpression on the degradation rate of N-Myc protein. HEK-293T cells were infected with lentivirus to express N-Myc protein and then transfected for 48 hours with plasmids to overexpress KLHL37. Before harvesting, the cells were treated with cycloheximide (CHX) (10 μg/mL) for the indicated durations. (**I**) Effect of KLHL37 overexpression on the ubiquitination of N-Myc protein in the cell system. HEK-293T cells with stable expression of N-Myc-HA were transfected with plasmids to overexpress KLHL37 and His-Ub for 48 hours. Then cells were treated with MG132 (10 μM) for 8 hours before being harvested. (**J**) Effect of KLHL37 on the ubiquitination of N-Myc in vitro with the RRL system. (**K**) Effect of KLHL37 overexpression on the endogenous ubiquitination of N-Myc. CHP-126 cells were transfected for 48 hours with plasmids to overexpress KLHL37. *P < 0.05, **P < 0.01, and ***P < 0.001, by 1-way ANOVA (**G**) and 2-way ANOVA (**H**). Data represent the mean ± SD in **G** and **H**.

To understand the molecular process of the regulatory effect of KLHL37 on *MYCN*-amplified neuroblastoma, we wonder whether KLHL37 could directly regulate N-Myc protein. The results showed that KLHL37 knockdown led to a dramatic reduction of N-Myc protein levels in *MYCN*-amplified tumors both in vitro and in vivo (Figure 3, A and B). In addition, shKLHL37 also induced a modest reduction of C-Myc in non-*MYCN*-amplified cells (Figure 3A). Correspondingly, KLHL37 overexpression resulted in a remarkable upregulation of endogenous N-Myc protein levels and a moderate effect on C-Myc protein levels in cancer cells (Figure 3C and Supplemental Figure 4A). Similar phenomena were observed in exogenous N-Myc and C-Myc proteins using an isogenic cell model (Figure 3, D and E, and Supplemental Figure 4B). Notably, overexpression of shRNA-resistant KLHL37 successfully rescued the downregulation of N-Myc levels caused by shKLHL37 (Figure 3F and Supplemental Figure 4C). Altogether, these results demonstrate that KLHL37 was capable of regulating Myc protein level.

To gain insights into the reason why KLHL37 could regulate N-Myc protein levels, we first analyzed the mRNA levels of *MYCN*. We found that N-Myc mRNA levels showed no significant change upon KLHL37 overexpression (Supplemental Figure 4D), suggesting that KLHL37 might be involved in the posttranslational regulation process of N-Myc. Given that N-Myc is a short-lived protein that undergoes rapid degradation through the ubiquitin proteasome system (15, 17), we speculated that KLHL37 might potentially cause an imbalance in the ubiquitination-mediated degradation of N-Myc. The results showed that the decline in N-Myc levels caused by shKLHL37 was restored by treatment with the proteasome inhibitor MG132 (Figure 3G). Meanwhile, overexpressed KLHL37 markedly prolonged the degradation half-life of N-Myc protein (Figure 3H). We further performed ubiquitination assays on HEK-293T cells and found that ubiquitination of N-Myc was obviously suppressed by KLHL37 overexpression (Figure 3I). Furthermore, we constructed an in vitro model based on recombinant KLHL37 and N-Myc proteins, using rabbit reticulocyte lysate (RRL) as a tool for enabling ubiquitination modification, and found that KLHL37 was capable of removing the ubiquitin chains from N-Myc (Figure 3J). A similar result was observed on endogenous N-Myc ubiquitination in neuroblastoma cells (Figure 3K). Collectively, these findings demonstrate that KLHL37 could increase the stability of N-Myc by blocking its ubiquitination degradation process.

### KLHL37 competes with the E3 enzyme to bind N-Myc and prevents its degradation.

**Figure 4:**
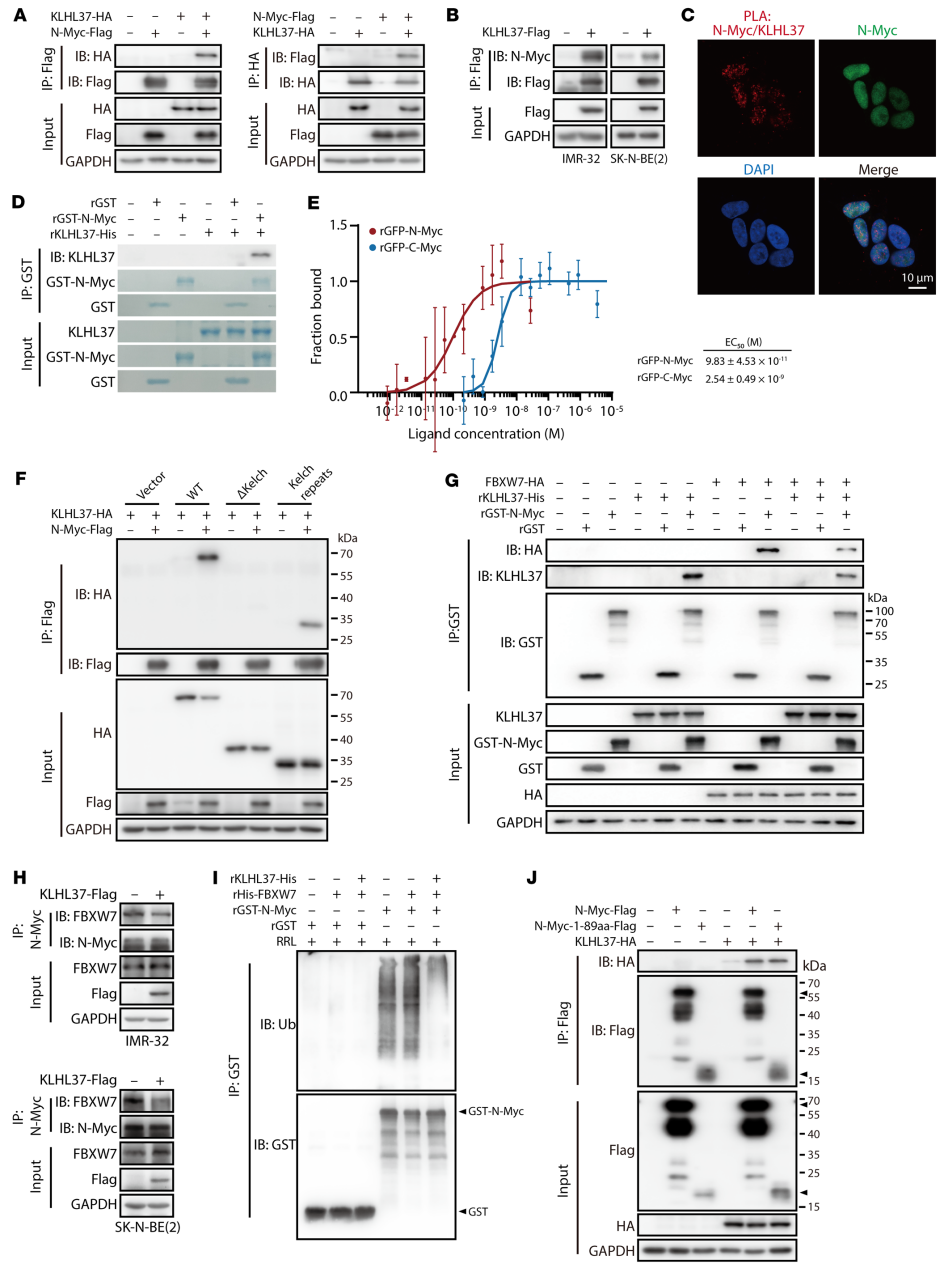
KLHL37 competes with the E3 enzyme to bind N-Myc and prevents its degradation fate. (**A**) The interaction between exogenous KLHL37 and N-Myc was detected using an IP assay. HEK-293T cells were transfected for 48 hours with plasmids overexpressing KLHL37-HA and N-Myc–Flag. (**B**) Cells were transfected for 48 hours with plasmids to overexpress KLHL37. KLHL37-Flag was enriched using anti-Flag resin, and then N-Myc signal was determined by immunoblotting. (**C**) A PLA was performed to detect the in situ interaction between KLHL37 and N-Myc proteins in SK-N-BE(2) cells. Scale bar: 10 μm. (**D**) Direct interaction between KLHL37-Flag and GST–N-Myc recombinant proteins in a cell-free system. KLHL37 bound to N-Myc was detected by immunoblotting, and other signals were visualized as Coomassie brilliant blue staining. (**E**) MST was performed to detect the interaction affinity of recombinant KLHL37 protein and recombinant GFP–N-Myc (rGFP–N-Myc) and GFP–C-Myc (rGFP–C-Myc) proteins. Mean ± SD.(**F**) HEK293T cells were transfected with different plasmids to overexpress N-Myc, WT KLHL37, and the truncated form of KLHL37 (Kelch repeats) for 48 hours. (**G**) Competition between KLHL37 and FBXW7 in binding to N-Myc proteins. GST or GST–N-Myc recombinant proteins were preincubated with KLHL37-His recombinant protein at 25°C for 4 hours. They were then incubated with cell extracts from HEK-293T cells overexpressing FBXW7-HA at 4°C for 12 hours. (**H**) Cells were transfected with plasmids to overexpress KLHL37 for 48 hours, and the interaction between endogenous N-Myc and FBXW7 was determined by co-IP and immunoblotting. (**I**) Effect of KLHL37 on the in vitro ubiquitination of N-Myc in a RRL cell-free system when recombinant FBXW7 protein was added to the system to promote N-Myc ubiquitination. (**J**) Interaction between KLHL37 and the N-Myc 1–89 aa segment. HEK-293T cells were transfected for 48 hours with plasmids to overexpress the indicated proteins, and the interaction was determined by immunoblotting after a co-IP assay.

To elucidate the mechanism underlying the regulation of N-Myc protein stability by KLHL37, we first investigated the potential direct interaction between KLHL37 and N-Myc. We found that KLHL37 interacted with exogenously overexpressed N-Myc (Figure 4A) and endogenous N-Myc by co-IP (Figure 4B). A proximity ligation assay (PLA) also indicated their in situ interaction in neuroblastoma cells (Figure 4C). Moreover, a direct interaction between recombinant KLHL37 and N-Myc proteins was further confirmed by co-IP (Figure 4D) and a microscale thermophoresis assay (Figure 4E). Besides N-Myc, we also observed the binding between C-Myc and KLHL37, which was weaker than that of N-Myc (Figure 4E and Supplemental Figure 5A). It is well known that the 6-repeat Kelch domain of the KLHLs family of proteins serves to form a pocket to capture substrate proteins (32, 33), thus we constructed KLHL37-ΔKelch and KLHL37-Kelch–repeat truncation mutants to clarify which region of KLHL37 is required for N-Myc binding (Supplemental Figure 5B). Co-IP results showed that deletion of the Kelch domain abolished the interaction between KLHL37 and N-Myc (Figure 4F), suggesting that N-Myc binds to the Kelch domain of KLHL37. Taken together, these findings uncover the interaction basis of the regulatory effect of KLHL37 on N-Myc.

It is established that KLHL family proteins act as E3 ligase adaptors, which bind to the scaffold protein Cullin 3 and in turn recruit Rbx1 and E2-Ub, thereby promoting the ubiquitination and degradation of substrate proteins (34–36). However, our findings showed that KLHL37 suppressed the degradation of N-Myc, which contrasts with the Cullin 3 E3 ligase function of other KLHLs. Accordingly, we hypothesized that the role of KLHL37 in regulating N-Myc might be independent of the KLHL–Cullin 3 E3 complex. To prove this, we deleted the 3-box region of KLHL37, which is crucial for binding Cullin 3 (32). The results showed that KLHL37 (Δ3-box) failed to interact with Cullin 3 as expected (Supplemental Figure 5C), but it still had the same effect as the WT KLHL37 in decreasing the ubiquitination levels of N-Myc (Supplemental Figure 5D). Furthermore, only the ΔKelch mutant could inhibit the upregulatory effect of KLHL37 on N-Myc, whereas the Δ3-box mutant failed to do so (Supplemental Figure 5E). In summary, these findings indicate that KLHL37 may regulate N-Myc protein independently of the KLHL–Cullin3 E3 complex.

FBXW7, the ubiquitin E3 ligase, has been found to be responsible for the proteasome-mediated degradation of N-Myc (37–40), thus we wondered whether KLHL37 stabilizes the N-Myc protein by disrupting FBXW7–N-Myc interaction to block its ubiquitination degradation. The results showed that recombinant KLHL37 could reduce binding between N-Myc and FBXW7 (Figure 4G). Meanwhile, the interaction between endogenous N-Myc and FBXW7 was also inhibited when KLHL37 was overexpressed (Figure 4H). Moreover, when the key N-Myc phosphorylation sites (T58, S62), which are crucial for FBXW7 binding (15, 17, 38), were mutated, the regulatory effect of KLHL37 overexpression as well as shKLHL37 on N-Myc was diminished (Supplemental Figure 6, A and B). These results indicate that KLHL37 may compete with FBXW7 in binding N-Myc. Correspondingly, using an in vitro ubiquitination system, we further observed that KLHL37 markedly suppressed the ubiquitination of N-Myc mediated by FBXW7 (Figure 4I). To understand the potential basis for the competition between KLHL37 and FBXW7, we introduced the 1–89 aa region of N-Myc, which is reported to be the essential segment for FBXW7 binding (41). Results showed that KLHL37 strongly bound to the 1–89 aa of N-Myc even when T58 and S62 residues were mutant, but failed to suppress the ubiquitination of N-Myc T58A/S62A (Figure 4J and Supplemental Figure 6, C–E), indicating that KLHL37 might compete with FBXW7 to bind the same region of N-Myc and that T58 and S62 residues of N-Myc protein are required for KLHL37’s interference with the stabilization of N-Myc protein. In summary, it is reasonable to postulate that KLHL37, when not functioning as a Cullin 3 scaffold E3 enzyme, competitively binds to N-Myc with FBXW7 and thereby enhances the protein stability of N-Myc.

### Discovery of RTA-408 as a KLHL37 inhibitor to induce N-Myc degradation.

**Figure 5:**
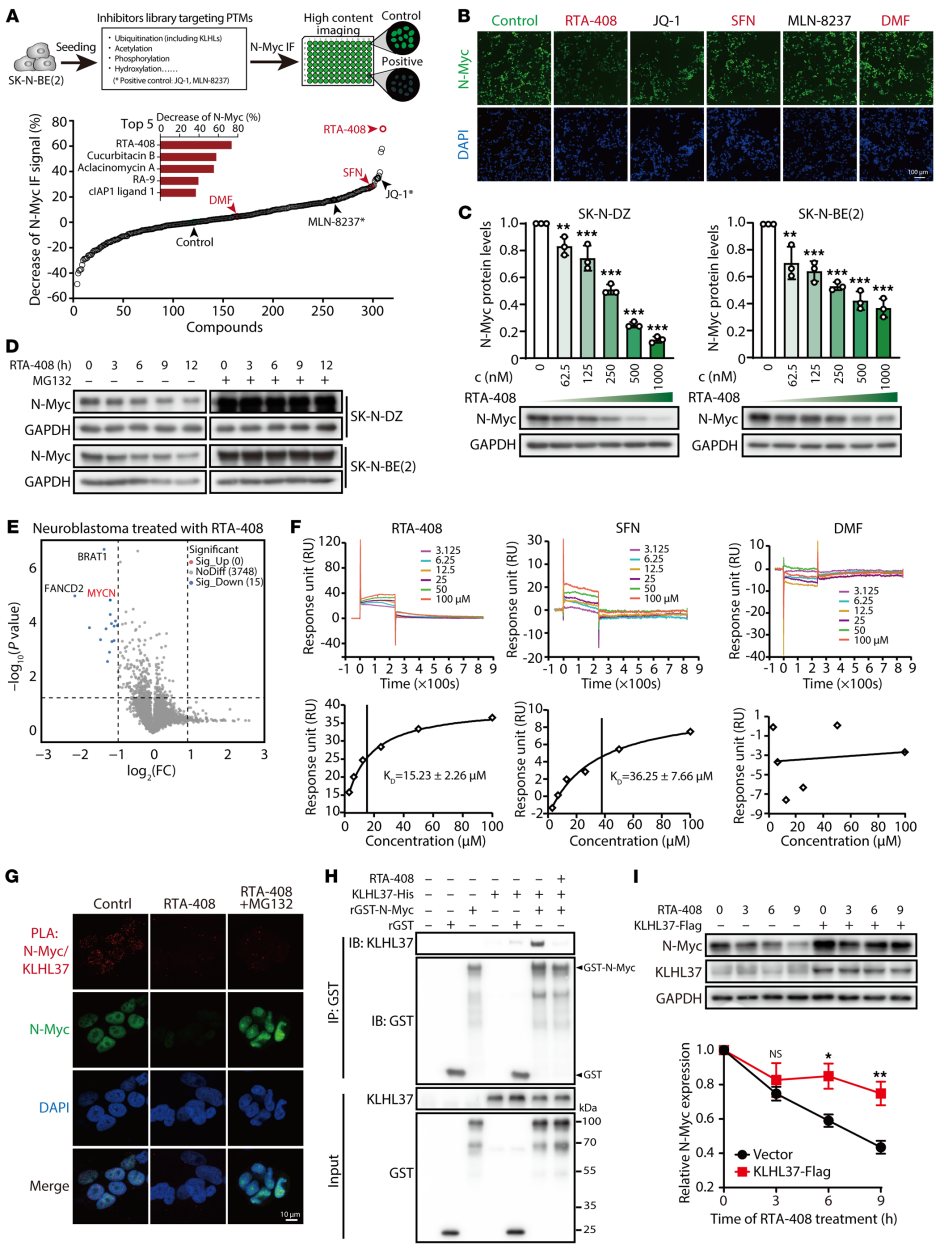
Discovery of RTA-408 as a KLHL37 inhibitor. (**A**) Schematic view of the high-content screening assay for compounds that downregulate N-Myc expression on the basis pf N-Myc immunofluorescence (IF). SK-N-BE(2) cells were treated with a compound library (1 μM) for 9 hours. The library was composed of 306 inhibitors of posttranslational modification enzymes. The compounds are ranked in descending order according to their effectiveness in decreasing N-Myc IF signals. (**B**) Representative IF images of N-Myc and DAPI staining in the groups treated with the indicated compounds. Scale bar: 100 μm. (**C**) Effect of RTA-408 treatment at the indicated concentrations (9 hours) on downregulating N-Myc protein expression. (**D**) Effect of the MG132 (10 μM, 3 hours) on the reduction of N-Myc protein expression induced by RTA-408 treatment. (**E**) Proteome-wide mass spectrometry was performed after neuroblastoma cells [SK-N-DZ, SK-N-BE(2) and CHP-126] were acutely treated with RTA-408 (1 μM, 3 hours). The protein changes identified in the proteomics analysis of the 3 cell lines are presented in the form of a volcano plot. A fold change of 2 or greater and a *P* value of less than 0.05 indicated significantly altered abundance. (**F**) Kinetics profile of binding of RTA-408, SFN, and DMF to KLHL37-His recombinant protein from the SPR analysis. The binding equilibrium dissociation constant (*K_D_*) of each compound were presented. (**G**) Representative images following the PLA to determine the in situ effect of RTA-408 on N-Myc–KLHL37 interaction in cells. SK-N-BE(2) cells were treated with RTA-408 (1 μM) as well as MG132 (4 μM) before the PLA. Scale bar: 10 μm. (**H**) The effect of RTA-408 on the direct interaction of KLHL37 and N-Myc proteins in a recombinant protein system. (**I**) The effect of KLHL37 overexpression on the degradation of N-Myc protein induced by RTA-408 treatment in SK-N-BE(2) cells. *P < 0.05, **P < 0.01, and ***P < 0.001, by 1-way ANOVA (**C**) and 2-way ANOVA (**I**) Data represent the mean ± SD in **C**, **F**, and **I**.

Direct targeting of N-Myc poses a considerable challenge due to the absence of a small-molecule compound binding pocket (42). Our findings have revealed a promising strategy to treat *MYCN*-amplified neuroblastoma by inhibiting KLHL37. Considering that KLHL37 functions in a posttranscriptional manner to regulate N-Myc, the compound library composed of 306 compounds that target various posttranslational modifications was constructed for the screening to identify inhibitors that reduce N-Myc (Supplemental Table 2). RTA-408 (omaveloxolone) had the most robust downregulating effect on N-Myc protein levels (Figure 5, A and B), and the degradation of N-Myc by RTA-408 occurred in a concentration-dependent manner (Figure 5C). Moreover, we found that RTA-408 induced the dramatic protein level decline of N-Myc without obvious changes in mRNA levels (Supplemental Figure 7A), and this downregulation could be restored by a proteasome inhibitor (Figure 5D), indicating that RTA-408 promoted the proteasomal degradation of N-Myc. Then, proteome-wide mass spectrometry results showed that N-Myc as well as BRAT1 and FANCD2 were the top 3 downregulated proteins upon RTA-408 treatment (Figure 5E and Supplemental Figure 7B). Since the mRNA levels of BRAT1 and FANCD2, which might be potential downstream targets of N-Myc, were decreased by RTA-408 (Supplemental Figure 7, C and D), we suspected that N-Myc might be the initial protein downregulated by RTA-408.

RTA-408 has also been reported as an inhibitor of KEAP1 (Kelch-like ECH-associated protein 1), disrupting its conformation necessary for binding to the substrate NRF2 (Nuclear factor erythroid 2-related factor 2) and effectively preventing the proteasomal degradation of NRF2 (43, 44). To determine whether the degradation of N-Myc mediated by RTA-408 relies on the inhibitory effect of KEAP1, we introduced an shRNA targeting KEAP1. Interestingly, we observed that shKEAP1 successfully upregulated NRF2 levels but did not lead to a significant change in N-Myc levels. And even in the presence of KEAP1 knockdown, RTA-408 could still decrease N-Myc levels (Supplemental Figure 8A), suggesting that RTA-408 may induce the degradation of N-Myc independently of KEAP1. Moreover, we noticed that sulforaphane (SFN) and dimethyl fumarate (DMF), which also function as inhibitors of KEAP1 (45), showed an effectiveness similar to that of RTA-408 in terms of increasing cumulative NRF2 protein levels. However, SFN and DMF were found to be less effective than RTA-408 in degrading N-Myc protein (Supplemental Figure 8B). These insights indicate that RTA-408 degrades N-Myc proteins through a mechanism that is independent of KEAP1.

Since KLHL37 is a critical regulator of N-Myc protein stability, we aimed to determine whether KLHL37 is involved in the effect of RTA-408 on N-Myc. We performed a surface plasmon resonance (SPR) assay to evaluate the binding affinity between RTA-408 and KLHL37 and observed a dose-dependent signal enhancement of RTA-408 binding to recombinant KLHL37 protein. The *K_D_* for the interaction between RTA-408 and KLHL37 was determined to be 1.523 ± 0.226 × 10^–5^ mol/L, meeting the *K_D_* value requirements of small molecules and proteins with a favorable affinity (≤1 × 10^–4^ mol/L). In contrast, SFN had a weaker binding affinity, while DMF did not show a significant affinity for KLHL37 protein (Figure 5F). The affinity results are consistent with the effects of RTA-408, SFN, and DMF on N-Myc degradation (Supplemental Figure 8B). Furthermore, 2 homologs of RTA-408 (45), RTA-401 and RTA-402, both showed remarkable efficacy in degrading N-Myc protein and inhibiting the growth of neuroblastoma cells (Supplemental Figure 8, C–F). To elucidate the dependency of N-Myc degradation on KLHL37 by RTA-408, we performed a PLA and observed a disruptive effect of RTA-408 on KLHL37–N-Myc interaction in neuroblastoma cells (Figure 5G and Supplemental Figure 8G). Meanwhile, RTA-408 effectively disrupted the direct interaction between recombinant KLHL37 and N-Myc proteins in vitro (Figure 5H). Moreover, overexpression of KLHL37 attenuated the degradation of N-Myc protein induced by RTA-408 (Figure 5I). Similar to shKLHL37, RTA-408 also failed to degrade the N-Myc (T58A/S62A) mutant that could not bind FBXW7 (Supplemental Figure 8H). Collectively, these findings indicate that RTA-408 functions as an inhibitor of KLHL37 to effectively promote the degradation of N-Myc by disrupting its interaction with KLHL37.

### KLHL37 inhibitor exerts dramatic antitumor effects against MYCN-amplified neuroblastoma cells.

**Figure 6:**
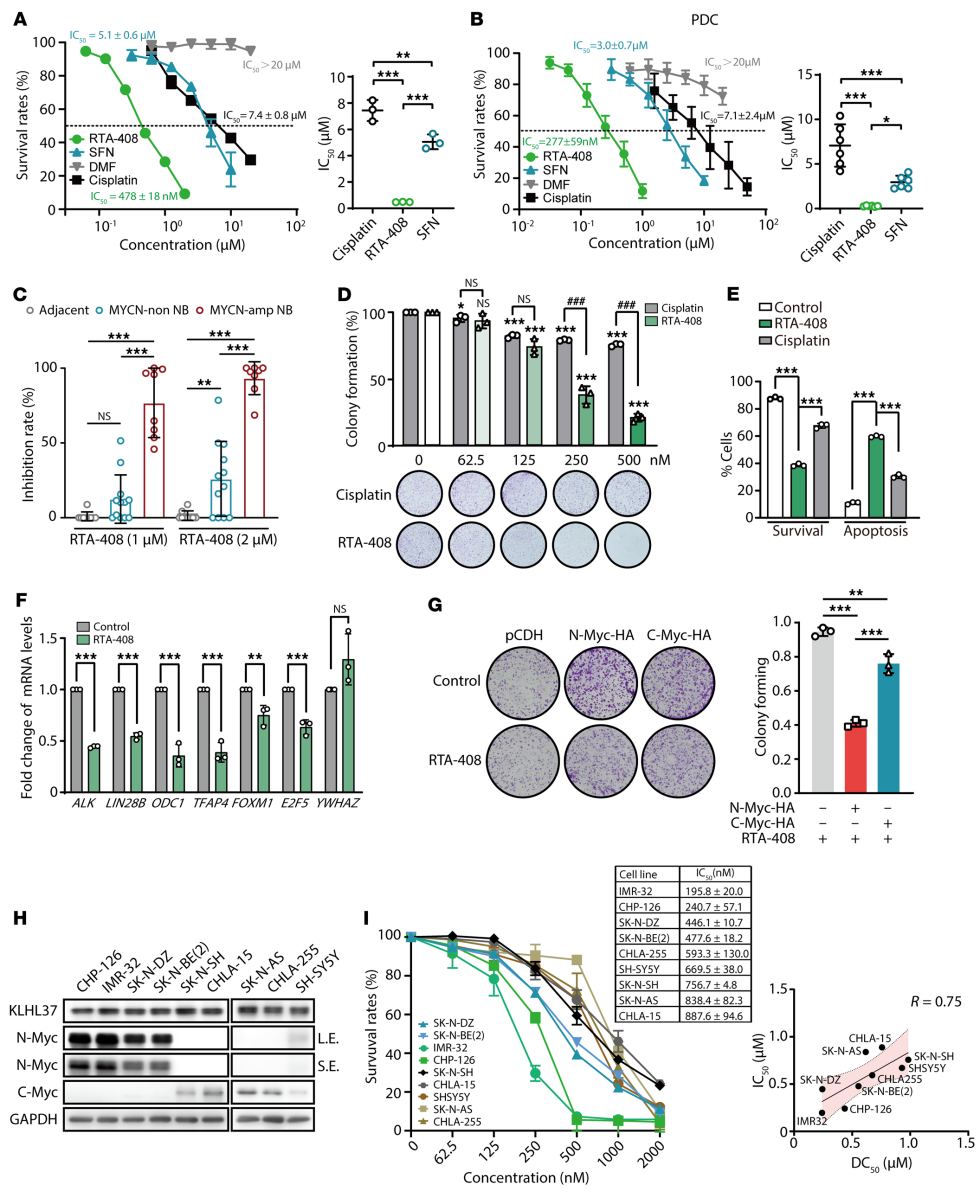
KLHL37 inhibitor exerts superior antitumor effects against *MYCN*-amplified neuroblastoma cells. (**A**) Proliferation of SK-N-BE(2) cells treated with RTA-408 (0.0625–2 μM), SFN (0.3125–10 μM), DMF (0.625–20 μM), or cisplatin (0.625–20 μM) for 72 hours, as measured by SRB assay. The IC_50_ values for each compound are shown as a scatter plot. (**B**) Proliferation of *MYCN*-amplified neuroblastoma PDCs treated with RTA-408 (0.03125–1 μM), SFN (0.3125–10 μM), DMF (0.625–20 μM), or cisplatin (1.5625–50 μM) for 72 hours, as measured by SRB assay. (**C**) PDCs from different patients with neuroblastoma were treated with RTA-408 (1 or 2 μM) for 72 hours, and then cell survival was determined by CellTiter-Glo Luminescent Cell Viability Assay. (**D**) Colony formation assay on SK-N-DZ cells treated with RTA-408 or cisplatin at the indicated concentrations. The colony formation rate was determined as the ratio of the number of clones in the RTA-408 (or cisplatin) treatment group to the number of clones in the control group. (**E**) Apoptosis of SK-N-DZ cells was determined by flow cytometry after cells were treated with RTA-408 or cisplatin (1 μM) for 48 hours. (**F**) Real-time qPCR analysis of representative *MYCN* downstream target genes in SK-N-DZ cells treated for 24 hours with RTA-408 (0.5 μM). *YWHAZ* gene is reported to be unaffected by N-Myc and was thus used as a reference control. (**G**) Effect of RTA-408 on the colony-forming ability of RD cells exogenously overexpressing N-Myc, C-Myc. (**H**) KLHL37, N-Myc, and C-Myc protein expression levels in different cancer cell lines were determined by immunoblotting. L.E., long exposure; S.E., short exposure. (**I**) Relationship of IC_50_ of RTA-408 and DC_50_ showing that RTA-408 promotes N-Myc, C-Myc degradation in different neuroblastoma cells. *P < 0.05, **P < 0.01, and ***P < 0.001, by 2-tailed Student’s *t* test (**F**) and 1-way ANOVA (**A**–**E**, and **G**). ^###^*P* < 0.001, and NS, no significance, by 2-tailed Student’s *t* test (RTA-408 vs. cisplatin groups) (**D**). Data represent the mean ± SD.

To assess the inhibitory potential of the KLHL37 inhibitor RTA-408 on the growth of *MYCN*-amplified neuroblastoma cells, SK-N-BE(2) cells were treated with RTA-408, SFN and DMF. As shown in Figure 6A, RTA-408 exerted a strong inhibitory effect on the proliferation of neuroblastoma cells, with an IC_50_ value of 478 ± 18 nM. In contrast, SFN, DMF, and the first-line chemotherapeutic drug cisplatin had higher IC_50_ values. Next, patient-derived neuroblastoma cells (PDCs) from patients with high-risk *MYCN*-amplified neuroblastoma were introduced. Results showed that RTA-408 also showed the highest degree of proliferation inhibition compared with SFN, DMF, and cisplatin in the PDC model (Figure 6B). Moreover, cells from adjacent tissue exhibited no obvious response to RTA-408, and non-*MYCN*-amplified cells showed a certain level of inhibition of cell proliferation. In contrast, *MYCN*-amplified cells were particularly sensitive to RTA-408 treatment, as evidenced by an inhibition rate of 93.2% ± 11.0% (Figure 6C), suggesting that RTA-408 shows pronounced therapeutic selectivity and potency in neuroblastoma cells driven by Myc. Additionally, compared with cisplatin, RTA-408 induced higher rates of both clonogenicity inhibition and apoptosis in *MYCN*-amplified neuroblastoma cells (Figure 6, D and E, and Supplemental Figure 9, A–C). Finally, real-time quantitative PCR (qPCR) analysis showed that RTA-408 resulted in significant downregulation of N-Myc target genes, including *ALK*, *LIN28B*, *ODC1*, *TFAP4*, *FOXM1*, and *E2F5* (46–52) (Figure 6F and Supplemental Figure 9D). *YWHAZ* served as a negative control, since it was unaffected by N-Myc regulation (53). Taken together, RTA-408, functioning as an inhibitor of KLHL37, showed a promising therapeutic effect on *MYCN*-amplified neuroblastoma cells.

To further investigate the role of N-Myc in the inhibitory effect of RTA-408, we first evaluated the therapeutic efficacy of RTA-408 in isogenic cells overexpressing N-Myc as well as C-Myc. The results showed that, compared with the control cells, RTA-408 had an obvious colony-forming inhibitory effect on N-Myc–overexpressing cells that was stronger than that on C-Myc–expressing cells (Figure 6G and Supplemental Figure 9E), which was consistent with the degradation efficiency of N-Myc and C-Myc (Supplemental Figure 9F). Moreover, we further investigated the correlation between the anti-neuroblastoma effect of RTA-408 and its Myc degradation efficiency. Given the distinct expression patterns of N-Myc and C-Myc in *MYCN*-amplified and non-amplified cells (Figure 6H), we assessed the degradation efficiency (half-maximal degradation concentration [DC_50_]) of N-Myc in *MYCN*-amplified cells and of C-Myc in non-*MYCN*-amplified cells upon RTA-408 treatment. The results showed that the degradation efficiency of N-Myc and C-Myc could form a great positive correlation with the IC_50_ of RTA-408 (Figure 6I and Supplemental Figure 10A). Meanwhile, the relative decline in the expression of Myc downstream genes (*ALK*, *ODC1*, and *TFAP4*) also formed a negative correlation with the IC_50_ value of RTA-408 (Supplemental Figure 10, B and C). In summary, these results revealed that RTA-408 exerted dramatic antitumor effects against *MYCN*-amplified neuroblastoma cells by promoting the degradation of N-Myc protein.

### Pharmacological inhibition of KLHL37 arrests the tumor growth of MYCN-amplified xenografts in vivo.

**Figure 7:**
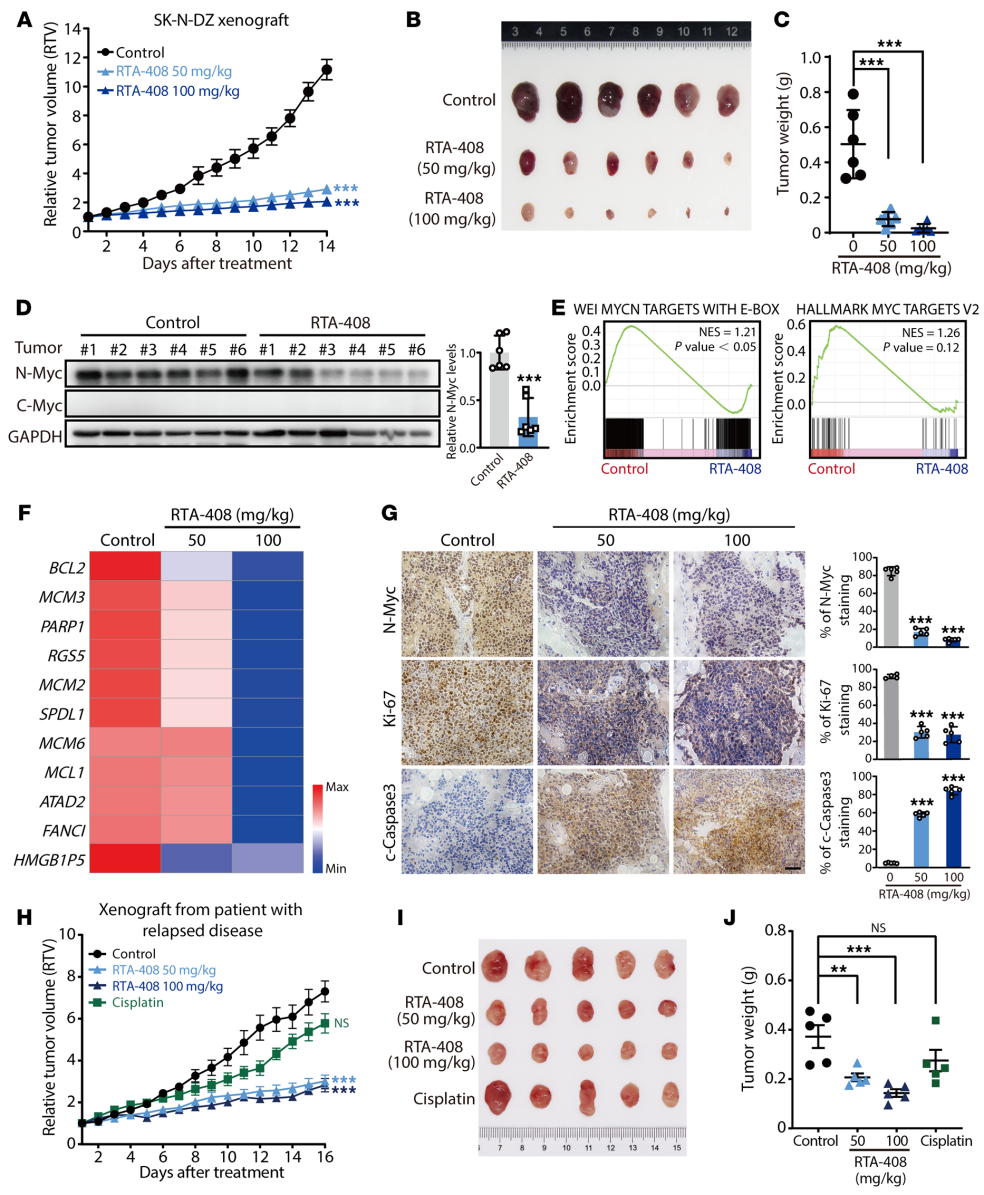
Pharmacological inhibition of KLHL37 arrests tumor growth of *MYCN*-amplified xenografts in vivo. (**A**) Tumor growth of SK-N-DZ xenografts. Tumor volume was measured every day, and the growth curves are drawn to show the mean ± SEM (*n* = 6). (**B**) Images of SK-N-DZ xenograft tumors were captured 14 days after RTA-408 administration. (**C**) Tumor weight of the SK-N-DZ xenografts 14 days after RTA-408 administration. (**D**) N-Myc and C-Myc protein levels in SK-N-DZ xenografts were detected by immunoblotting. (**E**) GSEA of the correlation of the *MYCN* target gene enrichment with KLHL37 inhibition via RTA-408 administration. (**F**) Heatmap of changes in expression of survival-related genes upon RTA-408 administration. (**G**) Histological staining for N-Myc protein and markers of proliferation and apoptosis in SK-N-DZ xenograft tumors. Scale bar: 100 μm. (**H**) Tumor growth of relapsed PDXs. Tumor volume was measured every day, and growth curves are drawn to show the mean ± SEM (*n* = 5). (**I**) Images of PDX tumors were captured 16 days after RTA-408 administration. (**J**) Tumor weight of the PDX 16 days after RTA-408 or cisplatin administration. **P < 0.01, and ***P < 0.001, by 2-way ANOVA (**A** and **G**), 1-way ANOVA (**C**, **E**, and **F**), and unpaired, 2-tailed Student’s *t* test (**D**). Data represent the mean ± SD in **C**, **D**, **G**, and **J**.

To further evaluate the therapeutic potential of RTA-408 against neuroblastoma in vivo, we established a cell-derived xenograft (CDX) model in mice using *MYCN*-amplified cells (SK-N-DZ) and non-*MYCN*-amplified cells (SK-N-AS). When the tumor volume reached 50–100 mm^3^, the mice were randomized into 3 groups and treated with CMC-Na solution (negative control) or RTA-408 (50 or 100 mg/kg/d) for 2 weeks. We observed that RTA-408 treatment significantly inhibited subcutaneous xenograft growth and markedly reduced the tumor weight in mice compared with the negative control (Figure 7, A–C, and Supplemental Figure 11, B–D). For the SK-N-DZ xenograft, RTA-408 showed significant therapeutic activity, with T/C values of 26.1% and 18.6% observed in the 50 and 100 mg/kg treatment groups, respectively (Supplemental Table 3). For the SK-N-AS xenograft, RTA-408 showed relatively moderate therapeutic activity, with T/C values of 85.0% and 69.3% (Supplemental Table 4). Meanwhile, we verified the degradation of N-Myc and C-Myc proteins in response to RTA-408 treatment with tumor tissues from a CDX (Figure 7D and Supplemental Figure 11E). These findings suggest that RTA-408 demonstrates a more potent therapeutic effect on *MYCN*-amplified neuroblastoma compared with non-*MYCN*-amplified neuroblastoma. This enhanced efficacy may be attributed to the stronger inhibitory activity of RTA-408 against N-Myc, as opposed to C-Myc. Further RNA-Seq analysis revealed that RTA-408 treatment could suppress N-Myc downstream targets and prosurvival genes (Figure 7, E and F). Additionally, immunohistochemical analysis showed that RTA-408 treatment depleted N-Myc expression concomitant with obvious intratumoral proliferation inhibition and apoptosis activation, as quantified by N-Myc, Ki67, and c–caspase 3 staining (Figure 7G and Supplemental Figure 11F). These results indicate the therapeutic potential of RTA-408 for *MYCN*-amplified neuroblastoma through N-Myc reduction.

To further investigate the therapeutic effect of RTA-408, we introduced a patient-derived xenograft (PDX) tumor based on *MYCN*-amplified neuroblastoma tissue from a patient with relapsed disease. The results showed that RTA-408 significantly arrested tumor growth, and did so better than cisplatin (Figure 7, H–J, and Supplemental Table 5), indicating the strong clinical therapeutic potential of RTA-408 for neuroblastoma. Additionally, we did not observe obvious body weight loss, and there were no significant alterations in organ or blood biochemical indicators following the administration of RTA-408 (Supplemental Figure 11, G–T), further demonstrating that RTA-408 is a promising therapeutic strategy for patients with neuroblastoma.

## Discussion

In this study, we have revealed that KLHL37 was aberrantly overexpressed in neuroblastoma cells and closely related with a poor prognosis for patients with neuroblastoma, especially those harboring *MYCN* gene amplification. Mechanistically, KLHL37 possesses a strong direct interaction with N-Myc and attenuates the access of the E3 ubiquitin ligase FBXW7 to N-Myc, thereby preventing N-Myc from degradation and enabling the carcinogenic properties. Notably, we demonstrate that the FDA-approved drug RTA-408 acted as a potent inhibitor of KLHL37, which disrupted the KLHL37–N-Myc interaction to facilitate the rapid degradation of N-Myc and neuroblastoma inhibition. These findings highlight the therapeutic potential of RTA-408 as a promising treatment strategy for neuroblastoma, especially for patients with *MYCN* amplification.

N-Myc, a widely recognized oncogenic driver, plays a pivotal role in neuroblastoma. In pathological conditions, various regulators, such as Aurora A (15, 16), PLK1 (17), HAUSP (54), and ALYREF (55), have been reported to stabilize N-Myc protein. These regulators counteract the function of E3 enzymes or enhance the activity of deubiquitinating enzymes. Here, we identify KLHL37 as a critical factor that maintains N-Myc stability. Interestingly, KLHL37 shares a similar mechanism with Aurora A to compete with FBXW7, enriching the mechanisms underlying the enhanced stability of N-Myc in neuroblastoma. However, whether KLHL37 competes with Aurora A to interact with N-Myc, or whether these 2 proteins collaborate to protect N-Myc from degradation driven by E3 ligases, remains to be investigated. Significantly, while Aurora A has been implicated in the regulation of multiple partners, it is noteworthy that KLHL37 appears to specifically target N-Myc as its substrate, as revealed in our study. This suggests that the functional role of KLHL37 may be more confined to neuroblastoma and its involvement in N-Myc stability.

Studies have revealed that KLHL37 is overexpressed in several cancer types and associated with poor prognosis, including colorectal carcinoma (22, 23), breast carcinoma (24), and lung cancer (56). These studies suggest that KLHL37 may possess carcinogenic potential. However, the molecular mechanism by which KLHL37 contributes to cancer progression remains to be elucidated. Our study reveals the procancer biological function of KLHL37, which works as a collaborating factor with N-Myc, in neuroblastoma. Importantly, we have proved that targeting KLHL37 leads to remarkable suppression of *MYCN*-amplified neuroblastoma in vitro and in vivo, indicting that KLHL37 is an appropriate therapeutic target for neuroblastoma. Additionally, we found that KLHL37 also had a regulatory effect on C-Myc, and we suspect that this effect may contribute to the carcinogenic potential of KLHL37 in non-*MYCN*-amplified neuroblastoma as well as other tumors, which merits further investigation. Certainly, there exists a differential regulatory effect of KLHL37 on N-Myc and C-Myc. We consider that those regulatory differences might result from the distinct binding affinities between KLHL37 and N-Myc and KLHL37 and C-Myc, and the detailed structural basis for these difference remains to be clarified in the future.

Direct pharmacological approaches to inhibiting N-Myc function have proven notoriously challenging, with research on N-Myc–targeting strategies long focused on interfering with *MYCN* gene transcription (9, 57), destabilizing the N-Myc protein (15–17, 54), and inhibiting crucial downstream oncogenes of N-Myc (3, 18, 58). So far, no targeted inhibitors of N-Myc have successfully reached the market or become available for clinical neuroblastoma treatment. Here, we reveal a pharmacological approach that specifically targets and disrupts the KLHL37–N-Myc complex and promotes N-Myc degradation by RTA-408 treatment. Notably, RTA-408 showed a higher inhibition rate on the N-Myc level compared with Aurora A and the BRD4 inhibitor, indicating that RTA-408 is a potent molecule that promotes the degradation of N-Myc protein. Encouragingly, the FDA recently approved RTA-408 (omaveloxolone) as the first treatment for Friedreich’s ataxia, a rare inherited degenerative disease, with a safety profile considered suitable for clinical application (59). Therefore, we propose RTA-408 as a promising drug candidate for fast translational application in treating *MYCN*-amplified neuroblastoma. Certainly, all KLHL family proteins share a similar secondary structure, characterized by the BTB/POZ domain, the BACK domain, and the Kelch domain (32, 33). Thus, RTA-408 may potentially target other KLHL family proteins including KLHL19, indicating that RTA-408 may indeed have KLHL37-independent effects, an idea that needs further study. Since RTA-408 is not a selective inhibitor of KLHL37, in the future, it would be beneficial to develop specific protein-protein interaction inhibitors that target the unique interface of the KLHL37–N-Myc complex, offering patients with neuroblastoma better therapeutic drug options.

In summary, our study provides the evidence identifying KLHL37 as a crucial regulator in the progression of *MYCN*-amplified neuroblastoma and provides valuable insights into the pathological mechanism of how N-Myc maintains high stability in neuroblastoma. Furthermore, we propose a strategy for the targeted disruption of the KLHL37-N-Myc complex, and suggest that RTA-408, an inhibitor of KLHL37, may serve as a potential pharmacological approach for the treatment of patients with *MYCN*-amplified neuroblastoma.

## Methods

### Sex as a biological variable.

Sex was not considered as a biological variable in this study. Female nude mice were used in this study.

### Cells and cell cultures.

The human neuroblastoma IMR-32, SK-N-BE(2), SH-SY5Y cell lines, NIH-3T3 cells, as well as the lung cancer NCI-H1299 cell line were purchased from the Shanghai Institute of Biochemistry and Cell Biology. The human neuroblastoma SK-N-DZ, SK-N-SH, SK-N-AS, CHLA-15, CHLA-255 cell lines, as well as the rhabdomyosarcoma RH30 and RD cell lines were provided by Ting Tao (The Children’s Hospital, Zhejiang University School of Medicine, Hangzhou, China). The CHP-126 cell line was a gift from Lingtao Wu (University of Southern California, Los Angeles, California, USA). The Human embryonic kidney HEK-293T and HEK-293FT cell lines were supplied by Invitrogen, Thermo Fisher Scientific.

SK-N-DZ, SH-SY5Y, RD, HEK-293T, NIH-3T3, and HEK-293FT cells were cultured in DMEM; IMR-32 and SK-N-SH cells were cultured in MEM supplemented with 10 mM pyruvate, 2 mM l-glutamine, and 0.1 mM nonessential amino acids. CHP-126, SK-N-AS, CHLA-15, CHLA-255, RH30, and NCI-H1299 cells were cultured in RPMI 1640 medium. All media were supplemented with 10% FBS (Gibco, Thermo Fisher Scientific) and 1% penicillin/streptomycin. All cells were supplemented with 10% FBS (Gibco, Thermo Fisher Scientific) and 1% penicillin/streptomycin. The cell lines were maintained at 37°C in a humidified atmosphere of 5% CO_2_ and passaged for a maximum of 2 months. PDCs from patients with neuroblastoma were culture in DMEM/F12 medium supplemented with 10 mM HEPES, 100 ng/mL hydrocortisone, 10 μg/mL transferrin, 400 pg/mL T3, 10 pg/mL glucagon, 1 ng/mL insulin, 100 pg/mL EGF, 200 pg/mL FGF2, 12% FBS (Gibco, Thermo Fisher Scientific), and 2% penicillin/streptomycin.

### Plasmids and reagents.

The full-length cDNA sequences of KLHL37, N-Myc, and FBXW7 were amplified from the total RNA extractions by PCR and then subcloned into pCDNA3.0, pCDH, or pSIN vectors. The indicated truncation sequences were amplified from the constructed KLHL37 or N-Myc plasmids and then subcloned into the pCDNA3.0 vector. The indicated N-Myc mutations were produced by site-directed mutagenesis following the manufacturer’s protocol (Transgene). The coding sequence of KLHL37-His (6×) was subcloned into the pET-28a vector for purification of prokaryotic recombinant proteins. The N-Myc and C-Myc coding sequence with N-terminal fusion of the GST tag was subcloned into the pFastBac Dual vector for purification of eukaryotic recombinant protein. The pEBB-His-C1-Ub plasmid was provided by Ezra Burstein (University of Michigan Medical School, Ann Arbor, Michigan, USA). The packaging plasmid pRΔ8.9 and envelope plasmid pMD.G were provided by Donald B. Kohn (UCLA, Los Angeles, California, USA). The shRNA oligonucleotides that target KLHL37 and KEAP1 were obtained from the open resource of MilliporeSigma and subcloned into pLKO.1 (shRNA sequences are shown in Supplemental Table 6).

The KLHL siRNA library (QTE-2735384G) was customized and purchased from Dharmacon. RTA-408, RTA-401, RTA-402, SFN, DMF, and MG132 were purchased from Selleck Chemicals and dissolved in DMSO to the final working concentration. Cisplatin powder was purchased from the Jiangsu Haosen Pharmaceutical Group and dissolved in DMSO to the final working concentration. The jetPRIME transfection reagent (no. 114-15) was purchased from Polyplus (Sartorius), and the RRL system was purchased from Promega. The PLA kit (DU092101) was purchased from MilliporeSigma. The CellTiter-Glo Luminescent Cell Viability Assay (G9243) was purchased from Promega.

### IP and Western blotting.

When detecting the interaction between KLHL37 and N-Myc, HEK-293T cells were transfected with plasmids that overexpressed proteins and were harvested 48 hours after transfection. Cells were harvested and lysed in RIPA lysis buffer (50 mmol/L Tris-base, 150 mmol/L NaCl, 1% Triton X-100, 0.1% SDS, and 0.5% sodium deoxycholate, pH 7.4) that was supplemented with 2 μg/mL leupeptin, 0.1 mM PMSF, and 0.1% (v/v) saturated sodium vanadate (Na3VO4) solution for 30 minutes on ice. To observe the effect of KLHL37 on polyubiquitination of N-Myc, cells were lysed in 4% SDS buffer (4% SDS, 150 mmol/L NaCl and 50 mmol/L triethylamine, pH 8.0). The whole-cell lysate was quantified using a BCA protein quantification kit (20201ES86, YESEN) and prepared for incubation with the indicated IP beads at 4°C overnight. Cell lysate in 4% SDS buffer was diluted 10 times before incubation with the IP beads. On the second day, the beads were washed with washing buffer (50 mmol/L Tris-HCl, 150 mmol/L NaCl, and 1% NP40, pH 8.0) five times and then denatured with loading buffer at 95°C for 15 minutes. Finally, the denatured samples were subjected to SDS-PAGE for separation, and target proteins were detected with the indicated antibodies.

### Antibodies.

Antibodies against N-Myc (D1V2A, no. 84406), C-Myc (D84C12, no. 5605), KEAP1 (D6B12, no. 8047), c–caspase 3 (no. 9661), Cullin 3 (no. 2759), and Flag (D6W5B, no. 14793) were obtained from Cell Signaling Technology. Antibodies against KLHL37 (7a, sc-517590) and Ub (P4D1, sc-8017) were purchased from Santa Cruz Biotechnology. Anti-NRF2 (ab62352) and anti–Ki-67 (ab9260) antibodies were obtained from Abcam. Anti-FBXW7 antibody (MA5-26563) antibody was purchased from Invitrogen (Thermo Fisher Scientific). Anti-FANCD2 (CPA7087) and anti-BRAT1 (CQA3925) antibodies were purchased from Cohesion Biosciences. Antibodies against GAPDH (db106) and HA (db2603) were obtained from Diagnostic Biosystems. Anti-His antibody (R130420) was purchased from HuaBio, and anti-GST antibody (A00865) was purchased from GenScript.

### High-content screening for compounds that regulate N-Myc using an inhibitor library targeting posttranslational modifications.

SK-N-BE(2) cells were seeded in a 96-well plate and treated with different compounds in the inhibitors library (1 μM) in each well for 9 hours. The inhibitor library was composed of 306 compounds that target various posttranslational modifications, including ubiquitination, acetylation, phosphorylation, methylation, hydroxylation, glycosylation, palmitoylation, and farnesylation (Supplemental Table 2). JQ-1 and MLN-8237 were used as positive controls. Then, we performed immunofluorescence staining for endogenous N-Myc protein. DAPI was used for nuclear staining to indicate cell numbers. High-content imaging technology was used to capture endogenous N-Myc fluorescence intensity and calculate the average N-Myc fluorescence intensity per cell.

### PLA.

Cells were plated beforehand in the fluorescence chamber slides. After fixation with 4% paraformaldehyde (in PBS) at room temperature (RT) for 15 minutes and permeabilization with Triton X-100, the cells were incubated at 4°C overnight with primary antibody pairs of different species targeting KLHL37 (sc-517590, Santa Cruz Biotechnology, mouse monoclonal, 1:200) and N-Myc (D1V2A, 84406, Cell Signaling Technology; rabbit polyclonal, 1:200). Next, secondary antibodies coupled to an oligodeoxynucleotide (single-stranded DNA), also known as the PLA probe, were added to bind the primary antibodies. In the presence of ligase and polymerase, oligodeoxynucleotides on neighboring PLA probes formed a closed loop and were amplified, followed by the addition of a fluorescein detection solution. An in situ PLA was conducted according to the manufacturer’s instructions. During the amplification step, Alexa Fluor 488–conjugated anti-rabbit antibody (Invitrogen, Thermo Fisher Scientific) was added 1:200 to the amplification solution to counterstain for N-Myc protein. Confocal images were observed under a confocal microscope.

### Real-time qPCR.

Total RNA extraction, reverse transcription to cDNA, and qPCR were performed as described previously (55). Real-time qPCR was used to detect mRNA levels of *KLHL37*, *MYCN*, and downstream target genes of N-Myc, and *GAPDH* was used as an internal standard for normalization. The primers used for real-time qPCR are listed in Supplemental Table 7.

### Cell proliferation and colony formation assays.

The cell proliferation assays for the KLHLs siRNA library screen and plate colony formation assays were performed as described previously (60).

### Lentivirus production and transduction.

Virus production, condensation, and transduction were performed as described previously (60).

### Recombinant protein purification.

Plasmids (pET-28a-KLHL37-His) containing the KLHL37-His, GFP-N-Myc, and GFP-C-Myc coding sequence were transformed into *E*. *coli* BL21 cells. After induction with isopropyl β-d-thiogalactopyranoside (IPTG) for 36 hours at 16°C, the bacteria in buffer (50 mmol/L imidazole, 12 mmol/L Na_2_HPO_4_, 8 mmol/L NaH_2_PO_4_ and 500 mmol/L NaCl) were lysed by a High-pressure Bacteria Breaker (Union Biotech). KLHL37-His protein was purified using a HisTrap HP column (Cytiva) and AKTA Pure 25L (GE Healthcare). For the SPR assay, KLHL37-His protein was further purified using a HiLoad 16/600 Superdex 75pg column (GE Healthcare) after elution from the HisTrap HP column.

Plasmids (pFastBac Dual-GST–N-Myc) containing the GST–N-Myc and GST-C-Myc coding sequence were transformed into *E*. *coli* DH10Bac cells and incubated for the blue/white screen. After 48 hours, a white single colony was selected for purification of GST–N-Myc bacmid DNA with the Bacmid Extraction Kit (Saint-Bio). Then, GST–N-Myc bacmid DNA was transfected into SF9 insect cells using X-treme GENE HP DNA transfection reagent (Roche), and the cells were incubated at 27°C with a shaking rate of 300 rpm for 96 hours to obtain P0 baculovirus. To generate P1 baculovirus, 400 μL P0 baculovirus was mixed with 40 mL SF9 cells (2.1 × 10^6^ cells per mL) and incubated at 27°C with a shaking rate of 125 rpm for 48 hours. For GST–N-Myc protein expression, 1.3 mL P1 baculovirus was mixed with 250 mL SF9 cells at (2.1 × 10^6^ cells per mL) and incubated at 27°C with a shaking rate of 125 rpm for 48 hours. Finally, SF9 cells were harvested and suspended with PBS buffer. The cell suspension was lysed by a High-pressure Bacteria Breaker (Union Biotech). GST–N-Myc protein was purified using a GST TRAP HP column (Cytiva) and AKTA Pure 25L. GST protein was obtained from the cleaving of GST–N-Myc with thrombin.

### SPR assay.

All SPR experiments were performed on a Biacore X100 (GE Healthcare) with Biacore Sensor Chip CM5 (Cytiva). For the protein immobilization assay, recombinant KLHL37-His protein was diluted to 200 μg/mL by sodium acetate (10 mmol/L, pH 3.6), and KLHL37-His was immobilized to the chip for 1,080 seconds to reach a saturation state. For the binding affinity assay, the concentrations of compounds were all set in a range between 50 μM and 3.125 μM through 2-fold dilution steps with PBS-P (2% DMSO) buffer. Next, the compound solution was injected at a flow rate of 20 μL/min for 240 seconds to incubate with KLHL37-His protein on the chip, followed with a dissociation for 600 seconds. Finally, the NaOH solution was injected to regenerate the chip surface for 180 seconds. All SPR data analyses were performed with Biacore Analysis software, and the *K_D_* value was calculated to determine the affinity of the compounds for binding KLHL37-His protein.

### Microscale thermophoresis assay.

Target protein dilution: GFP–N-Myc and GFP–C-Myc proteins were diluted to 200 nM using PBS-T buffer (PBS containing 0.05% Tween 20). For ligand protein dilution, because of the different affinities between KLHL37 and N-Myc or C-Myc, 2 different concentration gradients were set for the measurement. To determine the EC_50_ of KLHL37 with N-Myc, KLHL37-His protein was serially diluted 16 times starting from 27.3 nM. For the EC_50_ measurement of KLHL37 with C-Myc, KLHL37 was serially diluted 16 times starting from 7 μM. The target and ligand proteins were mixed at a 1:1 ratio and then sampled using capillaries for microscale thermophoresis (MST) measurement. The results were fitted using Monolith (NanoTemper, catalog MO-K022) analysis software to calculate the EC_50_ of the protein interaction.

### In vitro ubiquitination and co-IP assays.

To detect the effect of KLHL37 on the ubiquitination of N-Myc, recombinant GST–N-Myc and KLHL37-His were preincubated at RT for 3 hours. For the ubiquitination reaction, the mixture of recombinant protein was incubated in 20 μL reaction buffer containing 50 mmol/L Tris-HCl, 5 mmol/L MgCl_2_, 0.5 mmol/L DTT, an ATP-regenerating system (2 mmol/L ATP, 10 mmol/L creatine phosphate, 100 μg/mL creatine kinase), and 2.5 μL RRL (Promega). After incubation at 37°C for 1 hour, 1 mL NP40 buffer (50 mmol/L Tris-HCl, 150 mmol/L NaCl, and 1% NP40, pH 8.0) was added to terminate the reaction, and then the whole system was incubated with GST resin (BBI) at 4°C overnight for IP. On the second day, GST resin was washed with NP40 buffer 5 times and then denatured with loading buffer at 95°C for 20 minutes. Finally, the denatured samples were put into the SDS-PAGE for separation, and target proteins were detected with the indicated antibodies.

For the in vitro co-IP assay, recombinant KLHL37-His was incubated with GST or GST–N-Myc at RT (25°C) for 3 hours and then incubated with GST resin (preblocked with 1% BSA at 4°C for 3 hours) for IP. For the in vitro competition assay, recombinant GST or GST–N-Myc was preincubated with KLHL37-His at RT for 3 hours and then coincubated with HEK-293T lysate at 4°C overnight, which induced overexpression of FBXW7-HA by plasmid transfection, followed by IP with GST resin. For the interaction intervention assay, recombinant KLHL37-His was preincubated with RTA-408 (100 mM, dissolved in DMSO) at RT for 1 hour, and the final DMSO concentration was controlled to remain lower than 1%, then GST or GST–N-Myc was added for further interaction at RT for 3 hours, and final IP was performed with GST resin at 4°C overnight.

### Immunohistochemical staining.

For immunohistochemical staining, all tissue specimens were stained with 3% hydrogen peroxide (ZSGB-BIO) after deparaffinization and blocked by incubation with blocking buffer containing 5% goat serum (Gibco, Thermo Fisher Scientific). The specimens were treated with antibodies against N-Myc, c–caspase 3 (1:100), or Ki-67 (1:200) at 4°C overnight. The specimens were then incubated with HRP-conjugated secondary antibodies (ZSGB-BIO), stained with hematoxylin for nuclei, differentiated with hydrochloric acid ethanol, and, finally, mounted with neutral resin. All immunohistochemical slides were photographed using an Olympus microscope, and the images were quantitatively analyzed with ImageJ software (NIH).

### Animal experiments.

To generate a subcutaneous *MYCN*-amplified neuroblastoma xenograft model with KLHL37 deletion, SK-N-BE(2) cells were transduced with shControl (shCtrl) or shKLHL37 (no. 1 and no. 2) lentiviruses. On the second day of lentivirus infection, SK-N-BE(2) cells (1 × 10^7^ cells) were injected into the underarm of 5- to 6-week-old female nude mice (purchased from SLRC Laboratory Animal). Tumor formation, growth rate, and mouse body weight were recorded every 2 days. The mice were sacrificed at the end of experiment, then the tumor size was photographed, and the tumor weight was recorded. All animals were alive during RTA-408 administration, and each animal was euthanized at the specified point (day 14). To determine the in vivo therapeutic effect of RTA-408 on a *MYCN*-amplified neuroblastoma xenograft model, SK-N-DZ cells (1 × 10^7^ cells) were injected into the underarm of 5- to 6-week-old female nude mice (purchased from Shanghai Laboratory Animal Center [SLRC] Laboratory Animals). For the non-*MYCN*-amplified neuroblastoma xenograft model, SK-N-AS cells (1 × 10^7^ cells) were injected into the underarm of 5- to 6- week-old female NOG mice (purchased from Vital River Laboratory Animal Technology). Tumor-bearing mice were randomly divided into 3 groups: a control group, a low-dose RTA-408 (50 mg/kg) group, and a high-dose RTA-408 (100 mg/kg) group. The mice were treated daily for 2 weeks either with a solvent (CMC-Na suspension) or with RTA-408 administered intragastrically (i.g.). Throughout the treatment period, the tumor volume and mouse weight were recorded. At the end of the experiment, the tumor size was photographed, and the tumor weight was recorded upon sacrifice of the animals. Given the limited amount of tumor tissues, only partial samples were subjected to RNA-Seq.

For PDXs, tumor tissues from patients with neuroblastoma were cut into small pieces and injected into the underarm of nude mice. After the tumors had grown to approximately 100 mm^3^, the nude mice were randomly grouped and given a solvent (i.g.), RTA-408 (i.g.), or cisplatin (i.p.) for a 2-week period (2 days of dosing and 1 day off). The tumor volume and mouse weight were recorded every day, and blood samples were collected for investigation of complete blood counts and the metabolic panel before the animals were sacrificed. All animals were alive during RTA-408 administration, and each was euthanized at the specified time point (day 16).

### RNA-Seq assay.

RNA-Seq analysis was performed at Shanghai Biotechnology Corporation.

### Statistics.

Parameters measured through independent experiments were analyzed as average values along with the SD or the SEM. An unpaired, 2-tailed Student’s *t* test, 1-way ANOVA, or 2-way ANOVA was used to determine the statically significance of results in different groups. The results were considered significant when *P* was less than 0.05.

### Study approval.

For animal studies, we have complied with the guidelines approved by the IACUC of Zhejiang University in Hangzhou, China (IACUC-s20-033). All animal procedures were performed according to the guidelines approved by the IACUC of Zhejiang University. PDCs were extracted from tumor issues of patients with neuroblastoma (The Children’s Hospital, Zhejiang University School of Medicine). Written informed consents from patients and approval from the Institution Research Ethics Committee of the hospital were obtained before the use of these clinical materials for research purposes.

### Data availability.

All data associated with this study are present in this article or the supplemental materials. The RNA-Seq data are available in the NCBI’s Gene Expression Omnibus (GEO) database (GEO GSE294960). Values for all data points in graphs are reported in the Supporting Data Values file.

## Author contributions

MY and QH conceived the study and analyzed data. SX, PC, X Shi, HC, and LL performed the immunofluorescence and Western blotting procedures. HC, ZS, and XZ performed in vitro assays to evaluate KLHL37 regulation of Myc and proteome-wide mass spectrometry. AX and JZ performed the PLAs and cell viability assay of PDCs. SX and SB performed RNA-Seq and database analysis. SX and PC performed animal studies. PC and X Shi performed compound screening and in vitro recombinant protein assays. SX, X Shi, and JW collected clinical neuroblastoma samples and patient information. X Shao, JC, and BY conceived the experiments and helped organize the manuscript. MY and SX wrote the manuscript. The order of the co–first authors’ names was established on the basis of their equal overall contributions to the project, with SX listed first because of his lead role in organizing the manuscript and creating the original draft of the manuscript.

## Supplementary Material

Supplemental data

Unedited blot and gel images

Supplemental table 2

Supporting data values

## Version 1 06/10/2025

In-Press Preview

## Version 2 07/15/2025

Electronic publication
