# Supplementary material for: Disruption of the KLHL37–N-Myc complex restores N-Myc degradation and arrests neuroblastoma growth in mouse models
Source: J Clin Invest. 2025 Jun 10;135(14):e176655. doi: 10.1172/JCI176655 (PMC12259267; doi:10.1172/JCI176655)
Supplement: Supplemental data [file jci-135-176655-s061.pdf]

SUPPLEMENTARY FIGURES

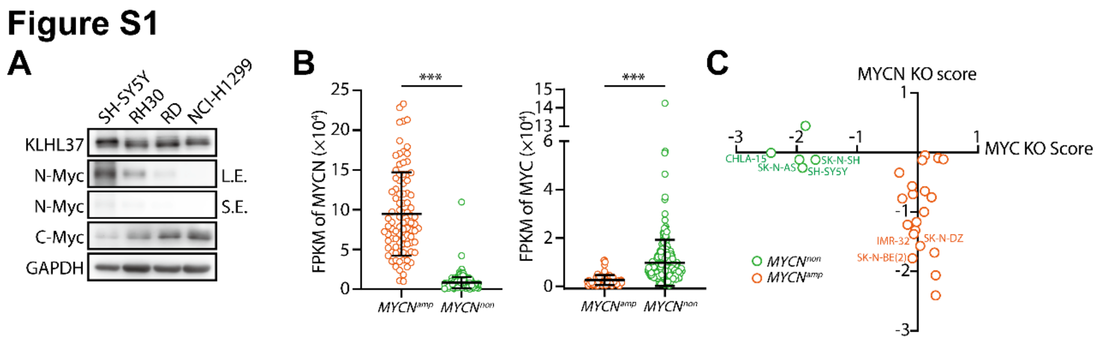

Supplementary Figure S1: Relationship between MYCN and MYC in cancer. (A) The endogenous expression of N-Myc and KLHL37 in SH-SY5Y, RH30, RD and NCI-H1299. L.E. (long exposure), S.E. (short exposure). **(B)** The N-Myc and C-Myc protein expression of neuroblastoma patients and data was derived from the GSE49710 cohort. **(C)** The score of neuroblastoma cell lines when *MYCN* or *MYC* is KO by CRISPR, and lower scores mean that cell survival is more dependent on the gene. Data was derived from DepMap Public dataset. Data was analyzed by unpaired 2-tailed Student's t-test (B).

Figure S2

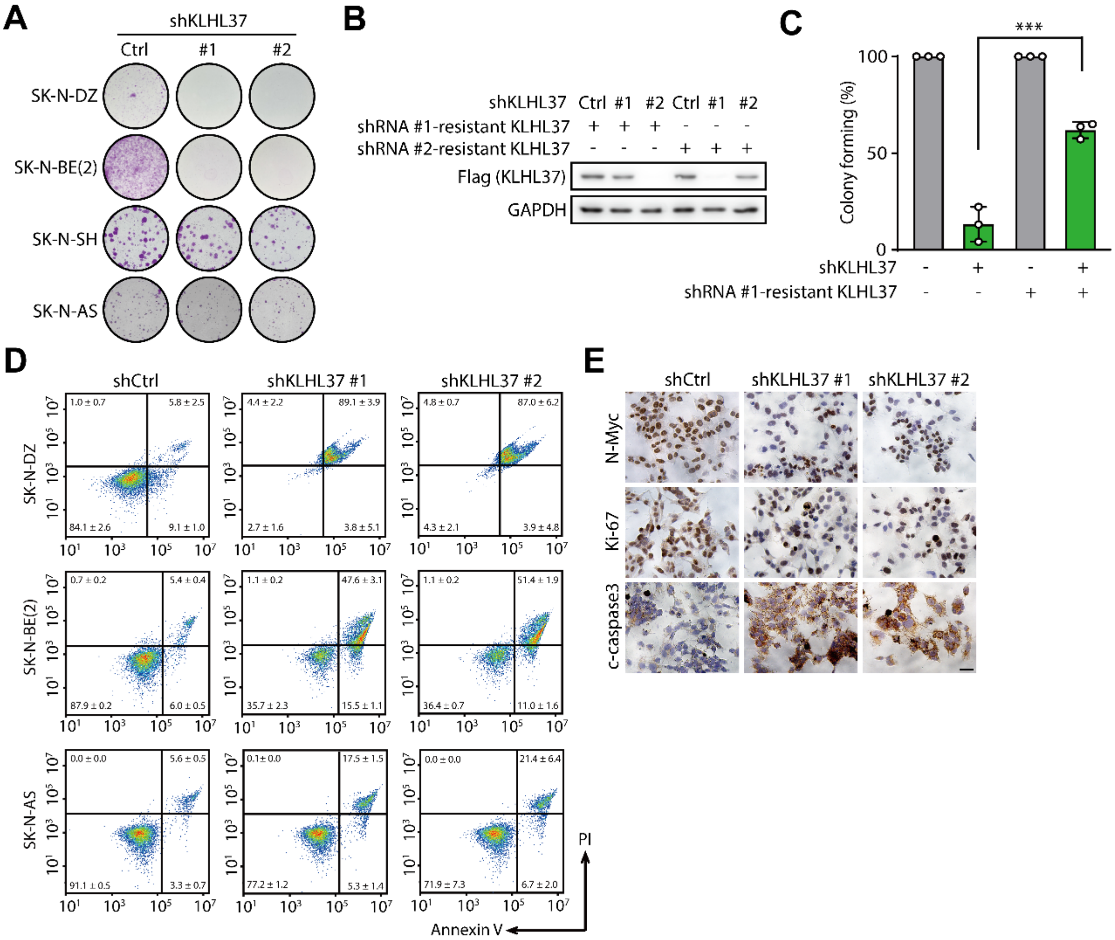

Supplementary Figure S2: The effect of KLHL37 depletion on the colony forming and survival of *MYCN*-amplified neuroblastoma cells. (A) Colony formation assay of neuroblastoma cells transduced with lentivirus-shKLHL37 (#1 and #2). Representative images were shown. **(B)** The resistance of KLHL37 synonymous mutants to the knockdown effect of shKLHL37 #1 and #2 in SK-N-DZ cells. **(C)** The resistance of KLHL37 synonymous mutants to the suppression of SK-N-DZ cell colony formation caused by shKLHL37 #1 in SK-N-DZ cells. **(D)** Representative flow cytometry plots and quantitative analysis of apoptosis induced by shKLHL37. **(E)** Representative images of the histological staining for N-Myc and markers of proliferation and apoptosis in SK-N-DZ cells. Scale bar represents 20 μm. Data was analyzed by unpaired 2-tailed Student's t-test (C).

Figure S3

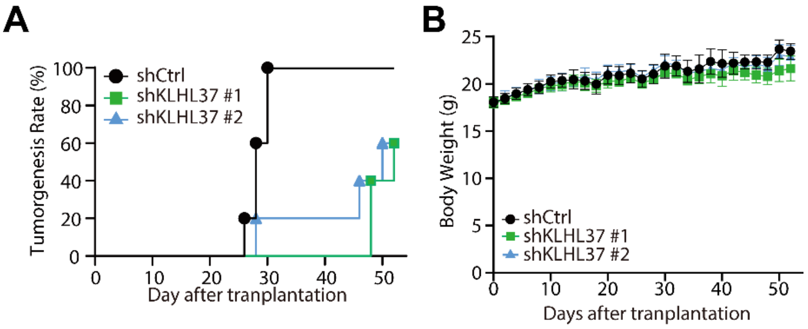

Supplementary Figure S3: The effect of KLHL37 depletion on the growth of *MYCN*-amplified neuroblastoma xenograft tumors. (A) The tumorigenesis rates of SK-N-BE(2) xenografts in shRNA control group or shKLHL37 (#1, #2) groups were calculated after transplantation. **(B)** Body weight of mice was measured every day since transplantation.

Figure S4

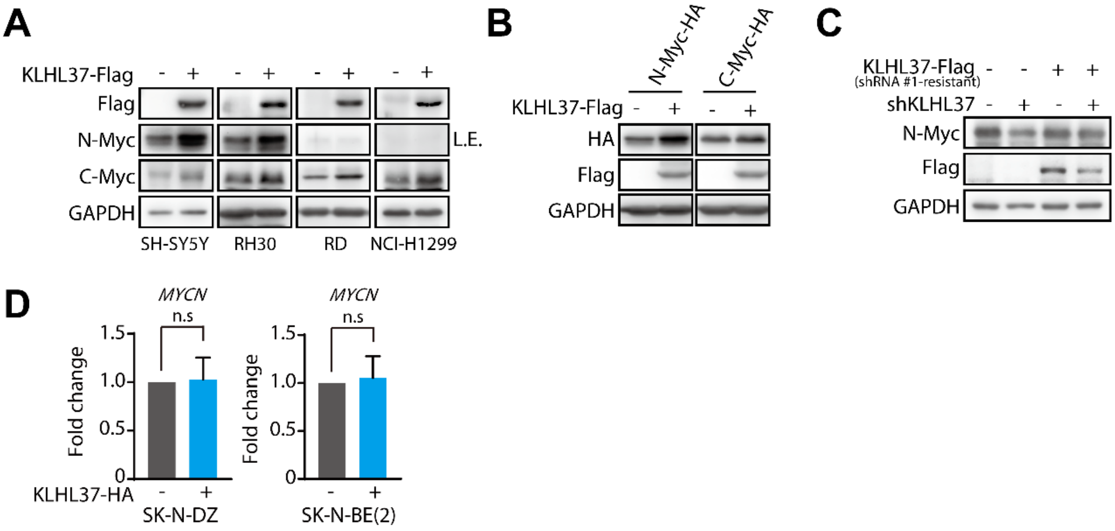

Supplementary Figure S4: Regulation of N-Myc and C-Myc protein by alterations in KLHL37. (A) The effect of KLHL37 overexpression on N-Myc and C-Myc protein expression in different tumor cells. Cells were transduced with lentivirus that overexpressed KLHL37 for 3 days. **(B)** The effect of KLHL37 overexpression on exogenously expressed N-Myc and C-Myc protein in HEK-293T cells. **(C)** The resistance of KLHL37 synonymous mutants to the

downregulation of N-Myc protein caused by shKLHL37 #1 in SK-N-DZ cells. **(D)** The effect of KLHL37 overexpression on the mRNA expression of *MYCN* gene in neuroblastoma cells. Cells were transfected with plasmids to overexpress KLHL37 for 48 h. Data was analyzed by unpaired 2-tailed Student's t-test (D).

Figure S5

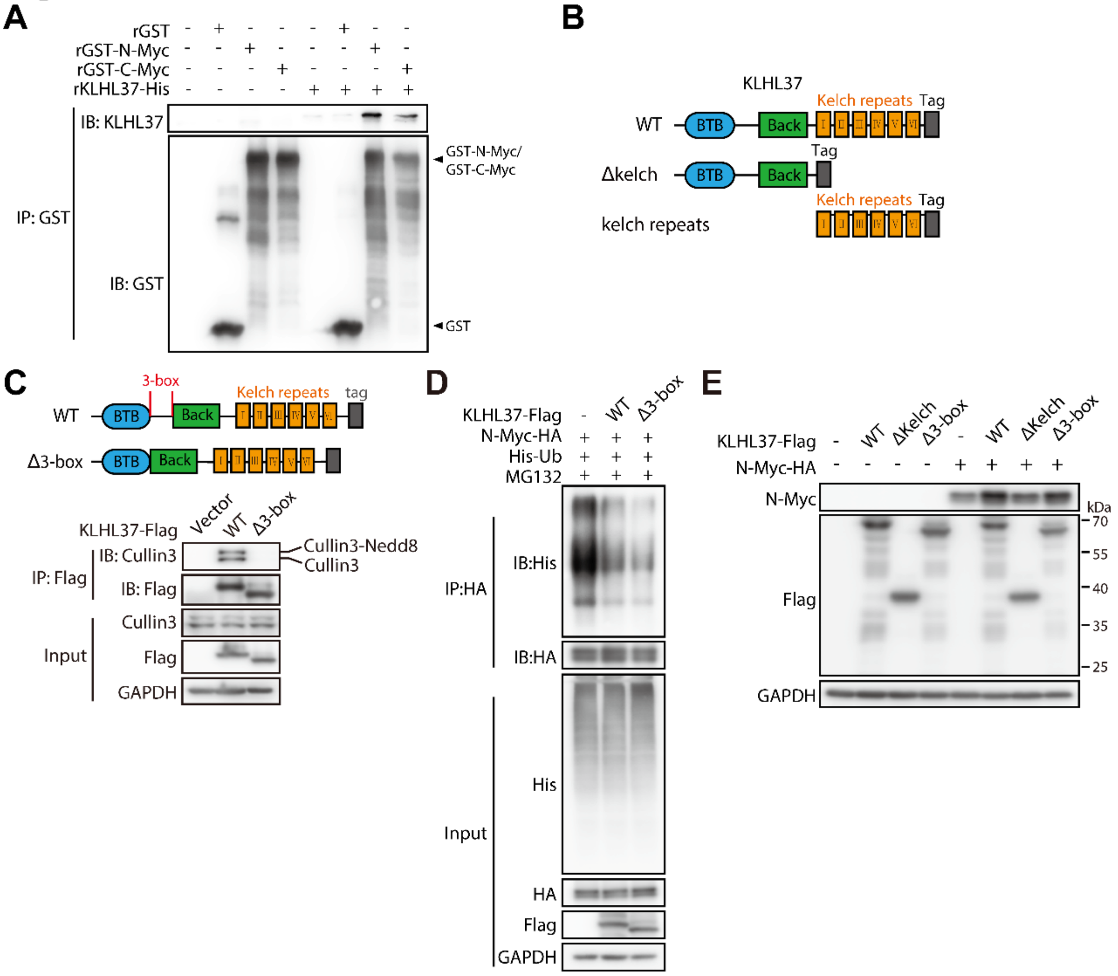

Supplementary Figure S5: Interaction with Cullin3 is not required for the regulation of N-Myc by KLHL37. (A) The direct interaction between recombinant KLHL37 and N-Myc, C-Myc proteins. **(B)** Scheme of KLHL37 region distribution and the interaction between N-Myc and KLHL37 deficient mutants. **(C)** Scheme of the key region (3-box) that is responsible for KLHL37 to interact with Cullin 3. The interaction between N-Myc and KLHL37-Δ3-box

was detected using an IP assay. **(D)** The effect of 3-box region deletion on KLHL37 regulation of N-Myc protein ubiquitination. HEK-293T cells expressing N-Myc-HA were transfected with plasmids for overexpressing either KLHL37 wild type or a mutant lacking the 3-box region, along with His-Ub, for 48 h. Cells were then treated with MG132 (10 μM) to inhibit proteasome activity for 8 h before harvest. **(E)** The effect of KLHL37 wild type, a mutant lacking the 3-box region, or a mutant lacking the Kelch repeats domain on the regulation of N-Myc expression in HEK-293T cells.

Figure S6

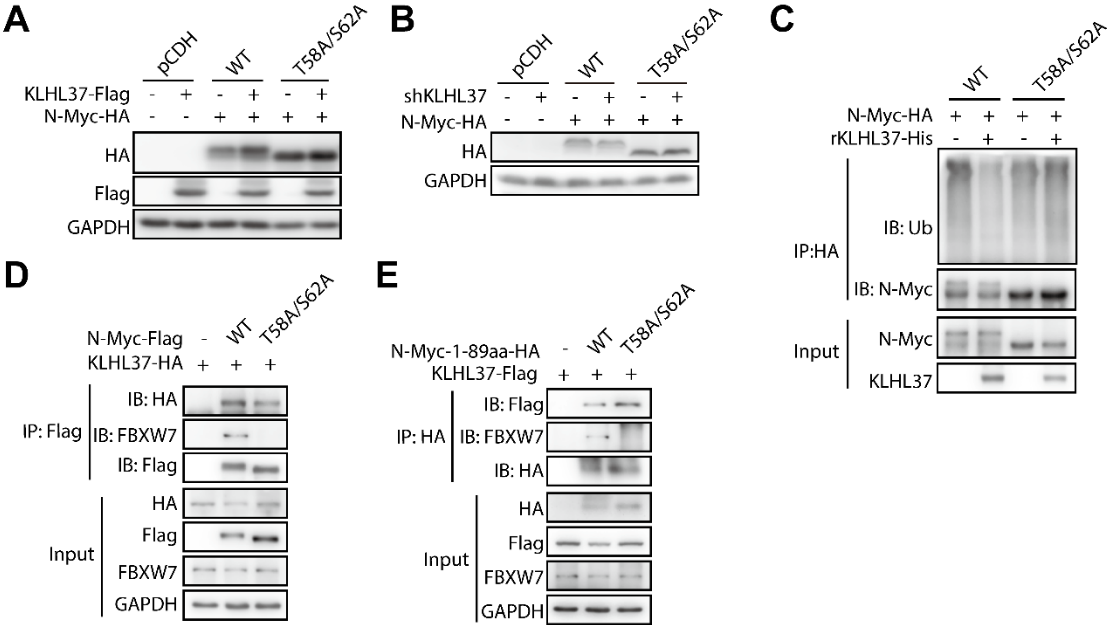

Supplementary Figure S6: The effect of KLHL37 on N-Myc mutants. (A) The effect of KLHL37 overexpression on the protein expression levels of N-Myc mutant (T58A/S62A) in HEK-293T cells, which was infected with lentivirus to stably expressed N-Myc-HA. **(B)** The effect of KLHL37 knockdown (sh #1) on exogenous overexpression of N-Myc wide-type or T58A/S62A mutant in RD cells. Cells were transduced with lentivirus to overexpress N-Myc for 3 days and then followed by transduction with shKLHL37 lentivirus for 3 days. **(C)** The

effect of rKLHL37-His protein on the ubiquitination of N-Myc wide type and T58A/S62A mutant protein in RRL system. **(D)** The interaction between exogenous KLHL37 and N-Myc wide type or T58A/S62A mutant was detected using an IP assay. **(E)** The interaction between exogenous KLHL37 and N-Myc-1-89aa wide type or T58A/S62A mutant was detected using an Co-IP assay.

Figure S7

A

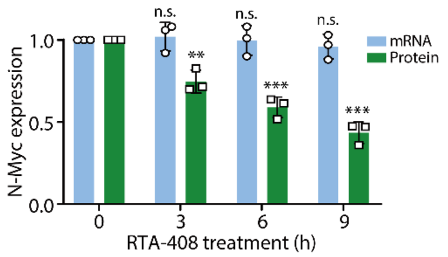

B

Downregulated proteins upon RTA-408 treatment

	Protein	P value	Relationship with Myc	References or GEO
1	BRAT1	1.65E-07	potential Myc downstream	GSE138295 (Chip-seq)
2	FANCD2	8.71E-06	potential Myc downstream<br>Myc protein interactors in gene transcription	GSE138295 (Chip-seq)<br>PMID 36424410 (Solvie D, 2022)
3	MYCN	1.24E-05	/	/
4	PI4KA	4.25E-05	potential Myc downstream	GSE138295 (Chip-seq)
5	TIPIN	7.36E-05	reported Myc downstream	PMID 18504438 (Wei JS, 2008)
6	TIMELESS	9.20E-05	potential Myc downstream<br>Myc protein interactors in gene transcription	GSE138295 (Chip-seq)<br>PMID 37340461 (Dong X, 2023)
7	IPO4	1.09E-04	reported Myc downstream	PMID 26771021 (Liberzon A, 2015)
8	NCAPD3	1.14E-04	potential Myc downstream	GSE138295 (Chip-seq)
9	TONSL	1.31E-04	potential Myc downstream	GSE138295 (Chip-seq)
10	POLE	1.43E-04	reported Myc downstream	PMID 18504438 (Wei JS, 2008)
11	ARMC6	3.63E-04	reported Myc downstream	PMID 32593299 (Wang H, 2020)
12	RRN3	3.92E-04	reported Myc downstream	PMID 21177653 (Poortinga G, 2011)
13	ZMYM4	4.25E-04	unknown	/
14	HEATR3	1.03E-03	potential Myc downstream	GSE138295 (Chip-seq)
15	CIP2A	2.28E-03	potential Myc downstream	GSE138295 (Chip-seq)

C

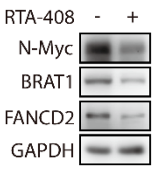

D

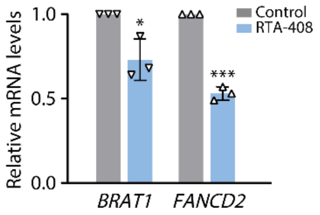

Supplementary Figure S7: The effect of RTA-408 acute treatment on N-Myc and other proteins. (A) The *MYCN* mRNA levels and N-Myc protein levels were detected when SK-N-BE(2) cells were exposed to RTA-408 treatment for indicated time. **(B)** The information of top

ranking proteins that significantly altered in abundance when neuroblastoma cells (SK-N-DZ, SK-N-BE(2) and CHP-126) were acutely treated with RTA-408 (1 μM, 3 h). **(C)** The effect of RTA-408 acutely treatment on top 3 ranking protein levels in SK-N-BE(2) cells. **(D)** The effect of RTA-408 acutely treatment on the mRNA levels of top 2 ranking proteins in SK-N-BE(2) cells. Data was analyzed by one-way ANOVA test (A), and unpaired 2-tailed Student's t-test (D).

Figure S8

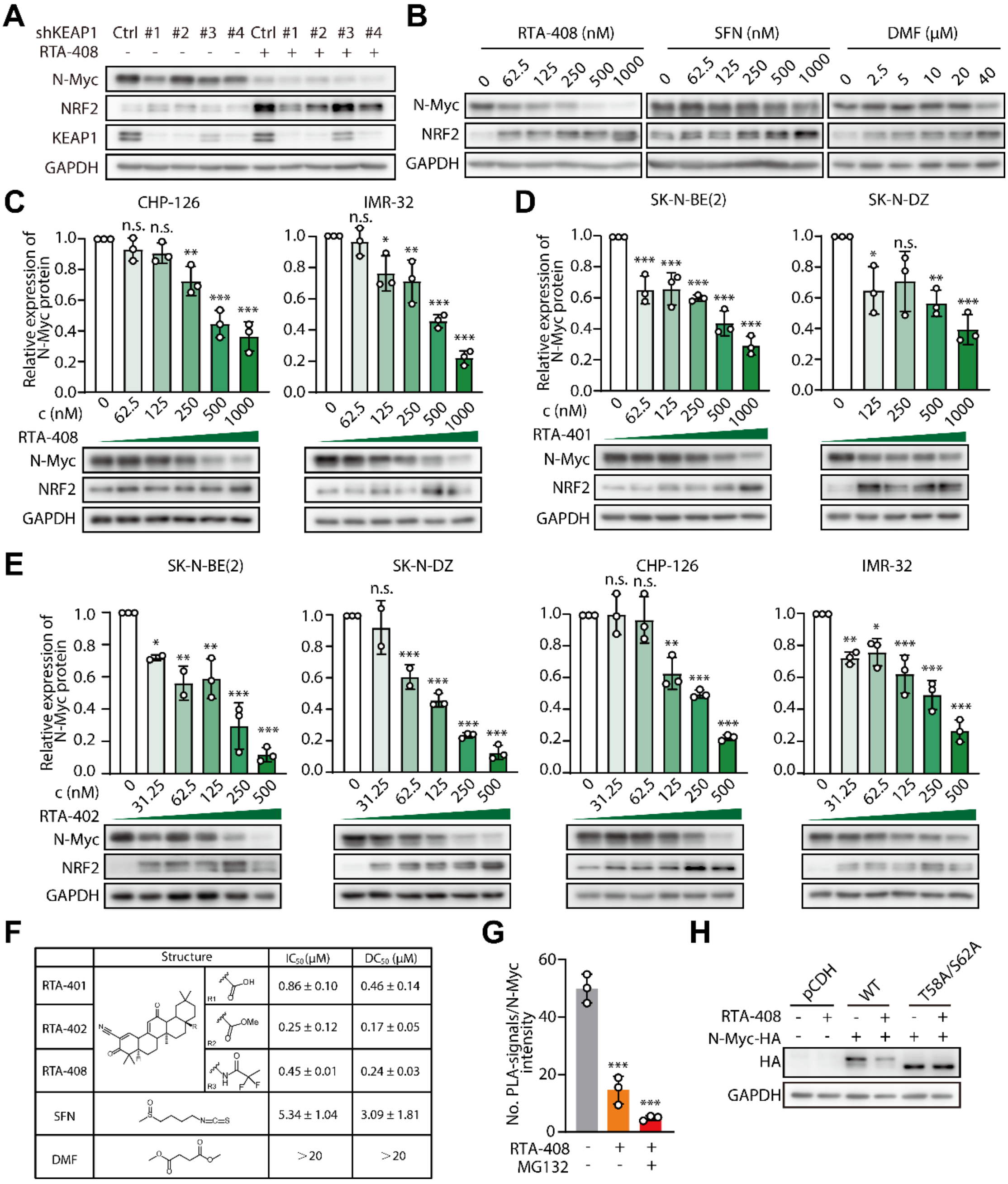

Supplementary Figure S8: The effects of several KEAP1 inhibitors on N-Myc protein degradation. (A) The relationship of RTA-408-mediated N-Myc degradation with KEAP1 depletion. SK-N-DZ cells were infected with shKEAP1 lentivirus for 3 days, then were treated with RTA-408 (1 μM) for 9 h. N-Myc protein expression was determined by immunoblot. **(B)** N-Myc and NRF2 protein expression was detected after the treatment of KEAP1 inhibitors with different concentrations in SK-N-DZ cells for 9 h. **(C)** The effect of RTA-408 on the

degradation of N-Myc protein in CHP-126 and IMR-32 cells. Cells were treated with RTA-408 at the indicated concentrations for 9 h. **(D)** The effect of RTA-401 (9 h) on the degradation of N-Myc protein in SK-N-BE(2) and SK-N-DZ cells. **(E)** The effect of RTA-402 (9 h) on the degradation of N-Myc protein in SK-N-BE(2), SK-N-DZ, CHP-126, and IMR-32 cells. **(F)** Summary of the effects of RTA-408, RTA-401, RTA-402, SFN, and DMF on N-Myc degradation, as well as their effects on the proliferation of SK-N-DZ cells. IC_{50}, concentration of half proliferation inhibition for 72 h. DC_{50}, concentration of half N-Myc protein degradation for 9 h. **(G)** Quantitative results of PLA experiments in which RTA-408 disrupts KLHL37/N-Myc interaction. **(H)** The effect of RTA-408 on exogenous overexpression of N-Myc wide-type or T58A/S62A mutant in RD cells. Cells were transduced with lentivirus to overexpress N-Myc for 3 days and then followed by treatment with RTA-408 (0.5 μM) for 9 h. Data was analyzed by one-way ANOVA test (C-E, G).

Figure S9

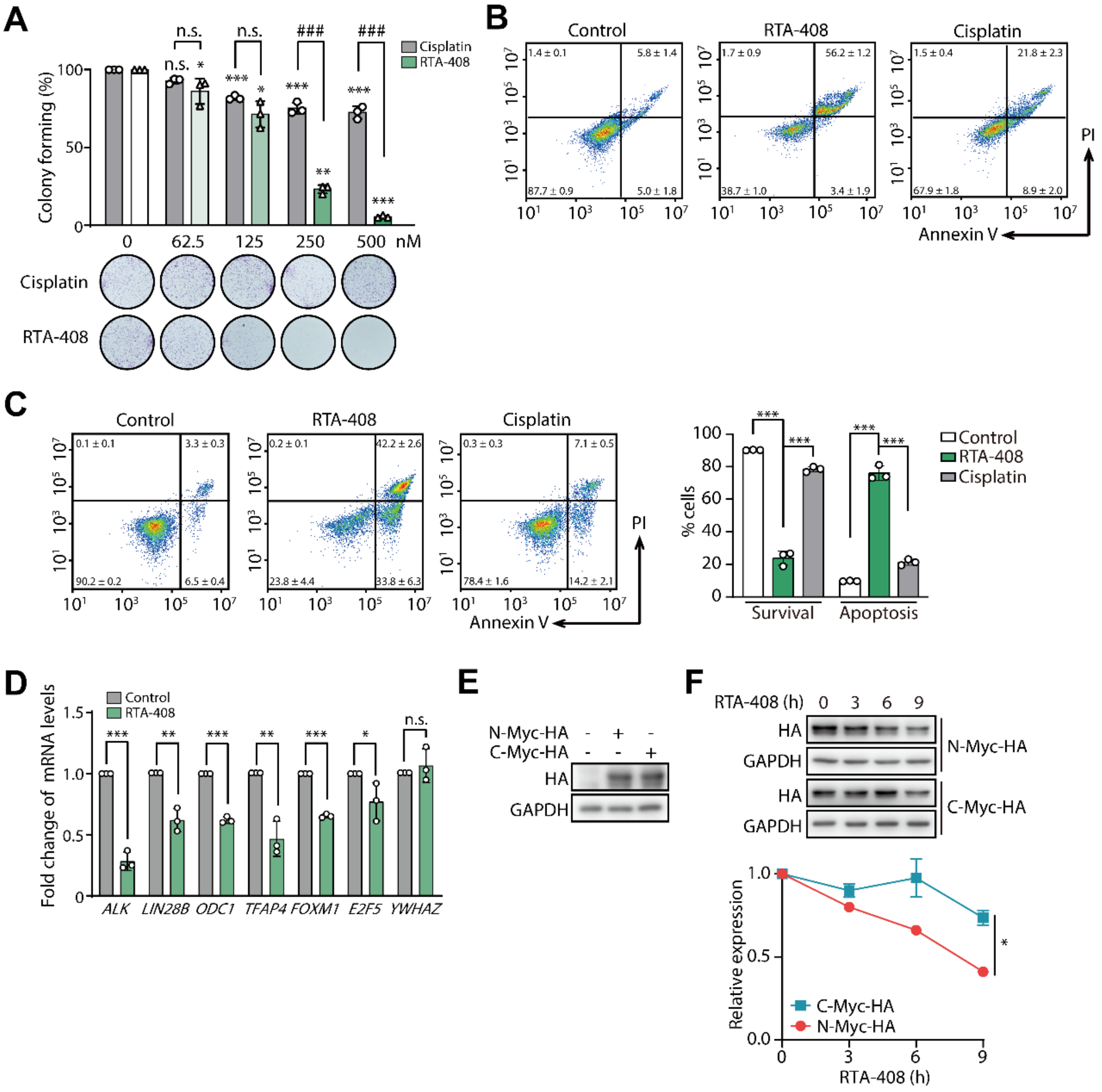

Supplementary Figure S9: The effect of RTA-408 on the colony forming ability and survival of neuroblastoma cells. (A) Colony formation assay on SK-N-BE(2) cells treated with RTA-408 or cisplatin at the indicated concentrations. Colony formation rate was determined as the ratio of the number of clones in RTA-408 (or cisplatin) treatment group to the number of clones in the control group. **(B)** Representative flow cytometry plots of apoptosis induced by RTA-408 treatment (1 μM, 48 h) in SK-N-DZ cells. **(C)** Representative flow cytometry plots and quantitative analysis of apoptosis induced by RTA-408 treatment (1 μM, 48 h) in SK-N-BE(2) cells. **(D)** Real-time qPCR analysis of representative *MYCN* downstream

target genes in SK-N-BE(2) cells with RTA-408 (0.5 µM) treatment for 24 h. *YWHAZ* gene was used as a reference control. **(E)** Overexpression of N-Myc or C-Myc in RD cells with lentivirus was determined by immunoblot. **(F)** The effect of RTA-408 on the degradation of N-Myc and C-Myc proteins. RD cells were infected with lentivirus to stably express N-Myc-HA or C-Myc-HA proteins and then treated with RTA-408 (0.5 µM) at the indicated times. N-Myc and C-Myc protein levels were quantitatively analyzed. Data was analyzed by one-way ANOVA test (A, C), unpaired 2-tailed Student's t-test (D) and two-way ANOVA test (F).

Figure S10

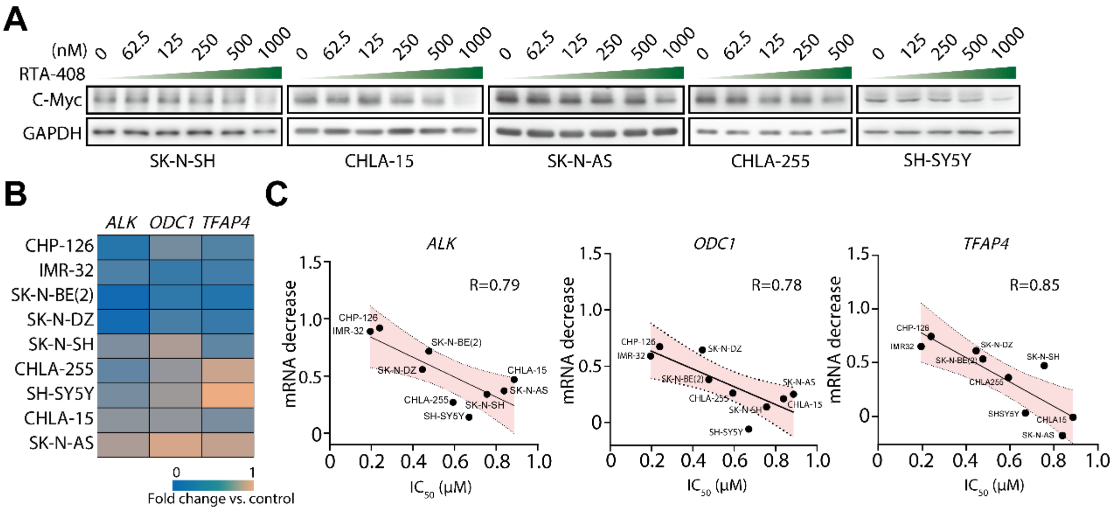

Supplementary Figure S10: The role of Myc signaling inhibition in the therapeutical efficiency of RTA-408. (A) The effect of RTA-408 treatment at the indicated concentrations for 9 h on C-Myc protein levels in SK-N-SH, CHLA-15, SK-N-AS, CHLA-255 and SH-SY5Y. **(B)** The effect of RTA-408 (0.5 µM, 24 h) on mRNA levels of *ALK*, *ODC1*, and *TFAP4* in different neuroblastoma cells. The mRNA downregulation *vs.* control was presented as a heatmap. **(C)** The relationship between downregulation of *ALK*, *ODC1*, *TFAP4* genes transcriptional levels and the therapeutical effect on neuroblastoma cells by RTA-408.

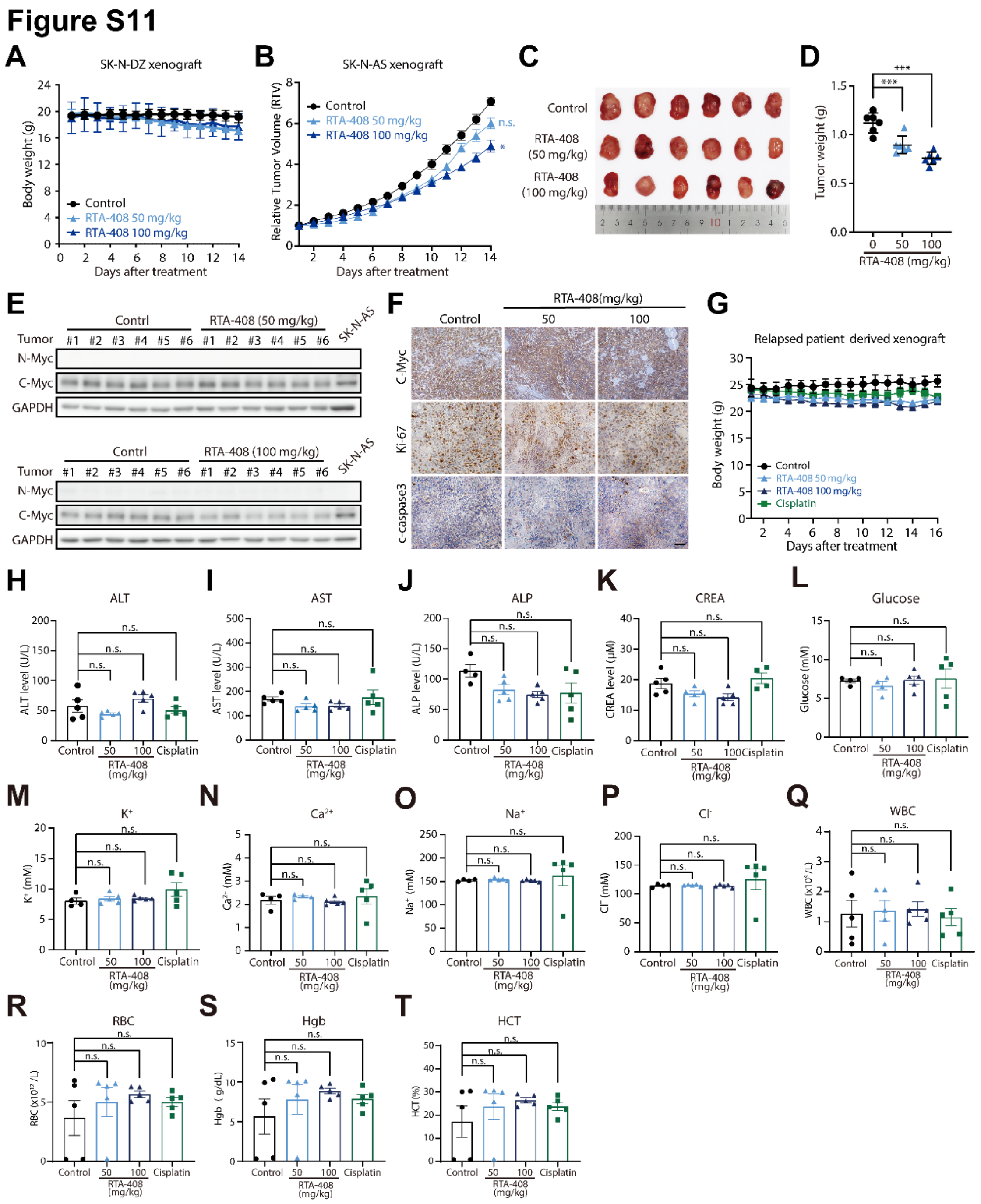

Supplementary Figure S11: The body weight, organ and blood biochemical indicators changes upon RTA-408 administration. (A) Body weight of CDX-bearing mice was measured every day, and growth curves were drowned. **(B)** Tumor growth of SK-N-AS xenografts. Tumor volume was measured every day and the growth curves were drown based as mean ± SEM (n = 6). **(C)** Image of SK-N-AS xenograft tumors were captured on the 14th

day after RTA-408 administration. **(D)** Tumor weight of the SK-N-AS xenografts on the 14th day after RTA-408 administration. **(E)** N-Myc and C-Myc protein levels of SK-N-AS xenograft were detected using immunoblot. **(F)** The histological staining for C-Myc protein and markers of proliferation and apoptosis of SK-N-AS xenografts. Scale bar represents 100 μm. **(G)** Body weight of PDX-bearing mice was measured every day, and growth curves were drowned. **(H-J)** Levels of ALT, AST, and ALP (liver function indicators) of mice after RTA-408 treatment for 16 days. **(K)** Levels of CREA (kidney function indicators) in response to RTA-408. **(L-T)** Other hematological and biochemical parameters change in response to RTA-408, including Electrolytes: glucose, K^+, Ca^{2+}, Na^+, Cl^- and Blood routine indicators: white blood cell count, WBC, red blood cell count, RBC, hemoglobin, Hgb, hematocrit, HCT. Data was analyzed by two-way ANOAV (B), one-way ANOVA test (D, H-T).

SUPPLEMENTARY TABLES

Table S1. Effect of KLHL37 depletion on the growth of SK-N-BE(2) derived xenograft

Groups	Tumor weight (g)	Inhibition (%)	RTV	T/C (%)
shCtrl	1.30 ± 0.87	—	16.76 ± 10.18	—
shKLHL37 #1	0.05 ± 0.06	96.38	1.06 ± 1.15	6.31
shKLHL37 #2	0.28 ± 0.36	78.18	4.23 ± 6.39	25.26

Table S1. The effect of KLHL37 depletion on tumor weight of mice at the terminal of the experiment. RTV, relative tumor volume. T/C (%) = $RTV_{Treatment}/RTV_{Control} \times 100\%$. Criteria for therapeutic activity: T/C (%), optimal growth inhibition < 50% means effective. N = 5.

Table S2. The high-content screening result on N-Myc protein level

Table S2 to Table S2.xlsx (Microsoft Excel, xlsx format)

Table S3. Effect of RTA-408 on the growth of SK-N-DZ xenograft tumors

Groups	Body weight (g)		Tumor weight (g)	Inhibition (%)	RTV	T/C (%)
	Start	End				
Control	19.4 ± 1.4	19.2 ± 2.0	0.504 ± 0.194	—	11.16 ± 1.72	—
RTA-408 (50 mg/kg)	19.7 ± 1.5	17.1 ± 0.8	0.077 ± 0.040	84.7	2.92 ± 0.62	26.1
RTA-408 (100 mg/kg)	19.0 ± 2.4	17.7 ± 2.0	0.024 ± 0.025	95.2	2.07 ± 0.28	18.6

Table S3. Effect of RTA-408 administration on CDX tumor weight and body weight at predose and postdose. N = 6.

Table S4. Effect of RTA-408 on the growth of SK-N-AS xenograft tumors

Groups	Body weight (g)		Tumor weight (g)	Inhibition (%)	RTV	T/C (%)
	Start	End				
Control	20.1 ± 0.6	20.0 ± 0.5	1.121 ± 0.038	—	7.07 ± 0.20	—
RTA-408 (50 mg/kg)	19.8 ± 0.6	20.0 ± 0.4	0.895 ± 0.033	20.1	6.01 ± 0.25	85.0
RTA-408 (100 mg/kg)	19.9 ± 0.4	20.0 ± 0.5	0.758 ± 0.023	32.3	4.90 ± 0.27	69.3

Table S4. Effect of RTA-408 administration on CDX tumor weight and body weight at predose and postdose. N = 6.

Table S5. Effect of RTA-408 on the growth of relapsed patient-derived xenograft tumors

Groups	Body weight (g)		Tumor weight (g)	Inhibition (%)	RTV	T/C (%)
	Start	End				
Control	24.3 ± 1.5	25.7 ± 1.0	0.372 ± 0.092	—	7.30 ± 1.00	—
RTA-408 (50 mg/kg)	22.5 ± 0.3	22.2 ± 0.9	0.207 ± 0.032	44.4	3.01 ± 0.64	41.2
RTA-408 (100 mg/kg)	23.1 ± 1.2	21.9 ± 1.3	0.143 ± 0.031	61.5	2.82 ± 0.65	38.7
Cisplatin	24.0 ± 0.8	22.6 ± 1.5	0.275 ± 0.087	26.1	5.78 ± 0.92	79.2

Table S5. Effect of RTA-408 administration on PDX tumor weight and body weight at predose and postdose. N = 5.

Table S6. Hairpin sequences of shRNA

	shRNA	Hairpin Sequences
1	shKLHL37#1	CCGGCGAGTCTGCAATTAACTGGATCTCGAGATCCAGTTAATTGCAGACTCGTTTTTG
2	shKLHL37#2	CCGGCTCTCTAAAGCAGGTAGAACACTCGAGTGTTCTACCTGCTTTAGAGAGTTTTTG
3	shKEAP1#1	CCGGGCGAATGATCACAGCAATGAACTCGAGTTCATTGCTGTGATCATTCGCTTTTTG
4	shKEAP1#2	CCGGGCAAGGACTACCTGGTCAAGACTCGAGTCTTGACCAGGTAGTCCTTGCTTTTTG
5	shKEAP1#3	CCGG CGGGAGTACATCTACATGCATCTCGAG ATGCATGTAGATGTACTCCCGTTTTTG
6	shKEAP1#4	CCGGGCACTGCAAATAACCCATCTTCTCGAGAAGATGGGTTATTTGCAGTGCTTTTTG

Table S7. Sequences of RT-PCR primers

	Primers	5'-3' sequences		Primers	5'-3' sequences
1	KLHL37-F	TCACAAGTCCTCCTACGCTG	6	TFAP4-F	GAGTATTTCATGGTGCCCACTC
	KLHL37-R	CTCTTTCAGGCCACCACTGA		TFAP4-R	CTCGGGGGTTAGTGGAATGT
2	MYCN-F	ACCACAAGGCCCTCAGTACC	7	FOXM1-F	TTTATCAGTGCTGCTAGCTGAGG
	MYCN-R	TCTCCACAGTGACCACGTCGATTT		FOXM1-R	TCTGAACTGGAAGCAAAGGAG
3	ALK-F	TCTCATCGCAGCCGATATGG	8	E2F5-F	TTCTGGATCTCAAAGCGGCTG
	ALK-R	GGCATCTCCTTAGAACGCTCT		E2F5-R	TGTAGCCACAACTTCTGCTGAT
4	LIN28B-F	AGAATCCCAGCCATGCACTT	9	YWHAZ-F	AGCTGGTTCAGAAGGCCAAA
	LIN28B-R	CTGCCTGACCGTTCTGAGAT		YWHAZ-R	AAGATGACCTACGGGCTCCT
5	ODC1-F	ATGCCTTCTATGTGGCAGACC	10	GAPDH-F	GTCATCCATGACAACTTTGG
	ODC1-R	CAGTCAAATCCTGTCCCGGT		GAPDH-R	GAGCTTGACAAAGTGGTCGT
