# Supplementary material for: Disruption of the KLHL37–N-Myc complex restores N-Myc degradation and arrests neuroblastoma growth in mouse models
Source: J Clin Invest. 2025 Jun 10;135(14):e176655. doi: 10.1172/JCI176655 (PMC12259267; doi:10.1172/JCI176655)
Supplement: Unedited blot and gel images [file jci-135-176655-s062.pdf]

Figure 3A

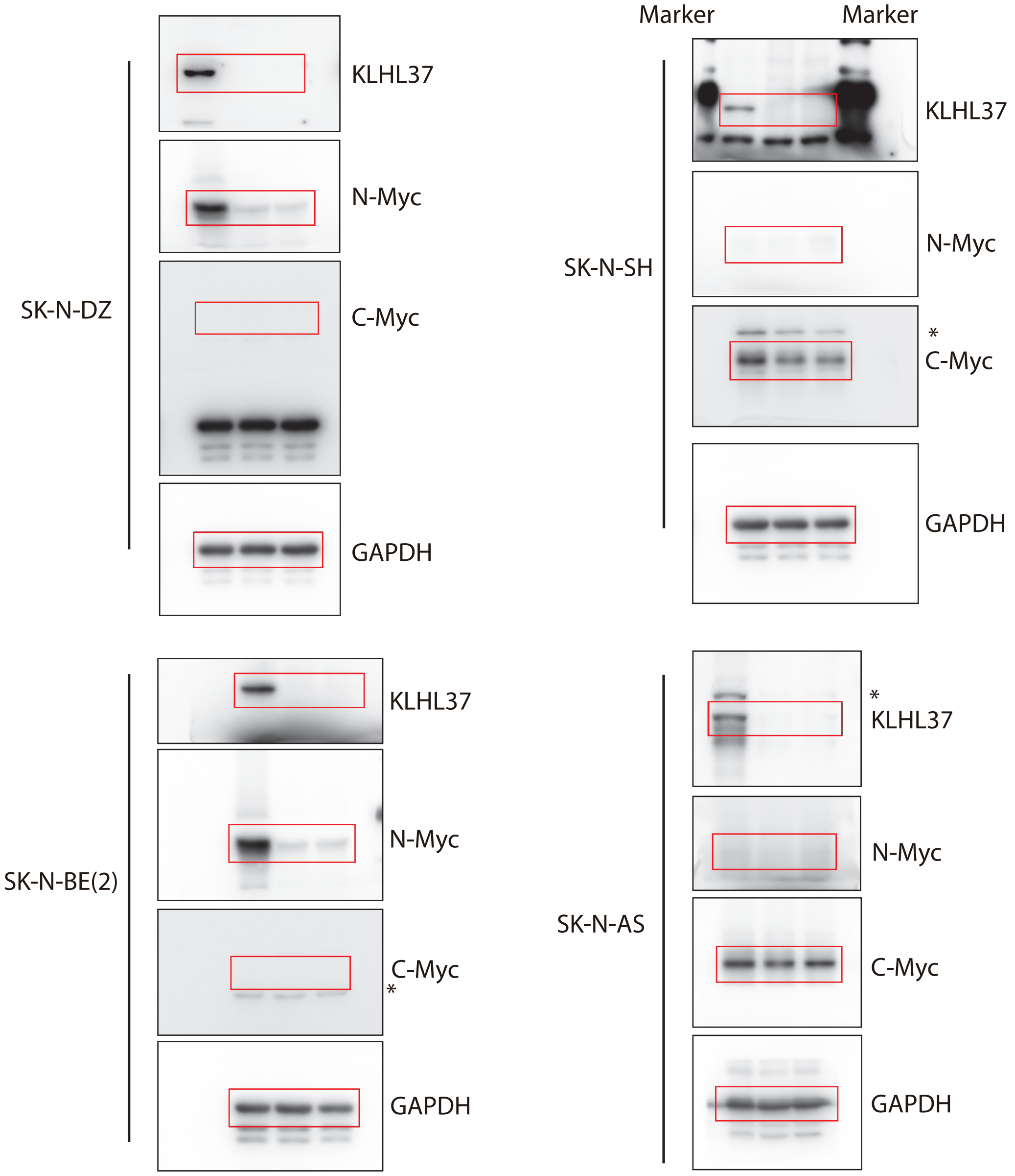

SK-N-DZ
KLHL37
N-Myc
C-Myc
GAPDH
Marker
Marker
SK-N-SH
KLHL37
N-Myc
*
C-Myc
GAPDH
SK-N-BE(2)
KLHL37
N-Myc
C-Myc
*
GAPDH
SK-N-AS
*
KLHL37
N-Myc
C-Myc
GAPDH

Figure 3B

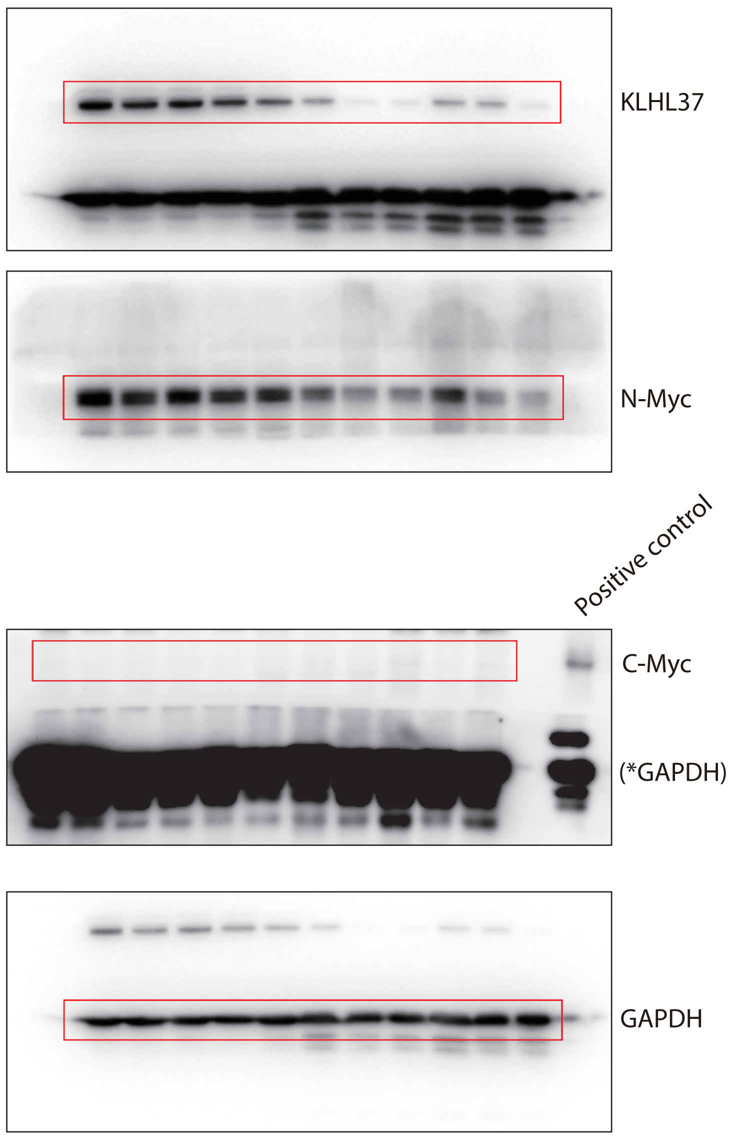

Figure 3C

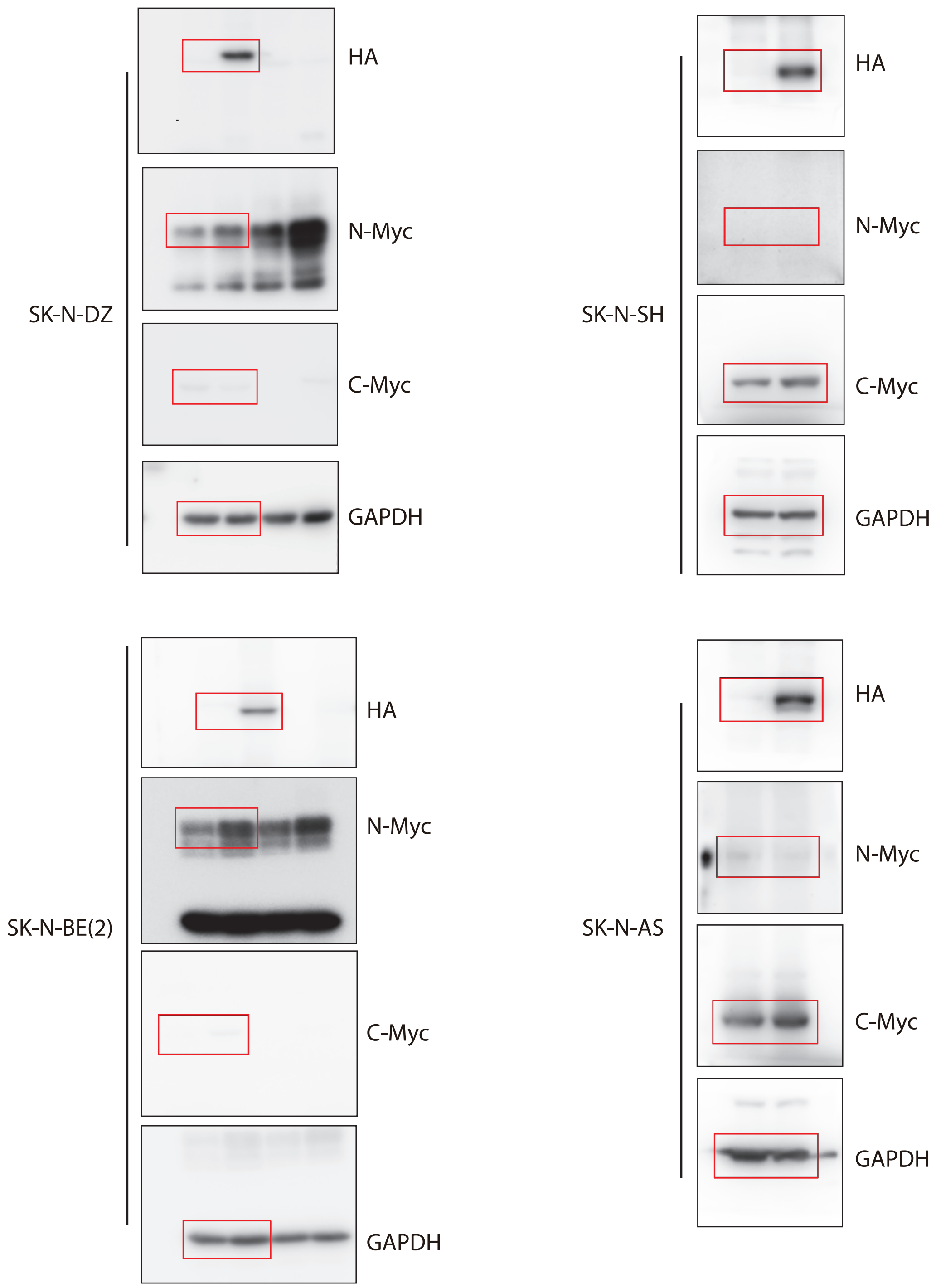
HA
N-Myc
C-Myc
GAPDH
SK-N-DZ
HA
N-Myc
C-Myc
GAPDH
SK-N-SH
HA
N-Myc
C-Myc
GAPDH
SK-N-BE(2)
HA
N-Myc
C-Myc
GAPDH
SK-N-AS

Figure 3D

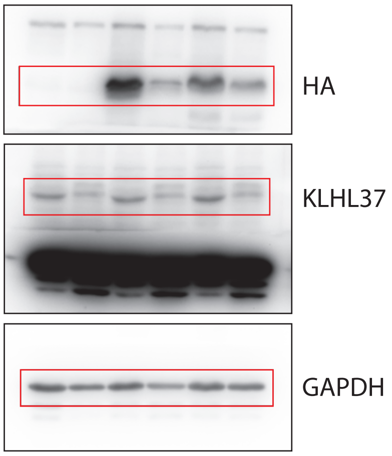

Figure 3E

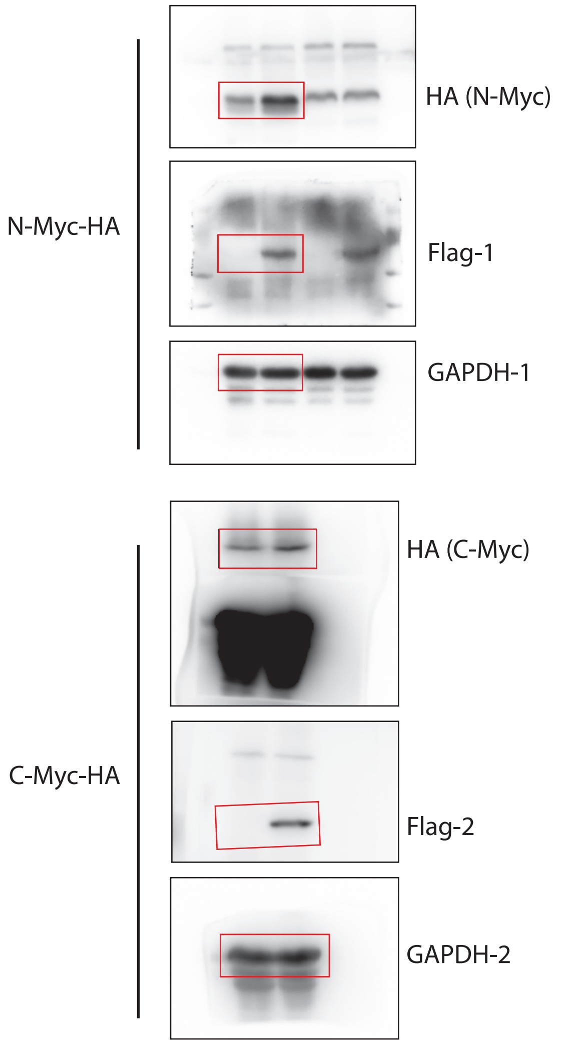

Figure 3F

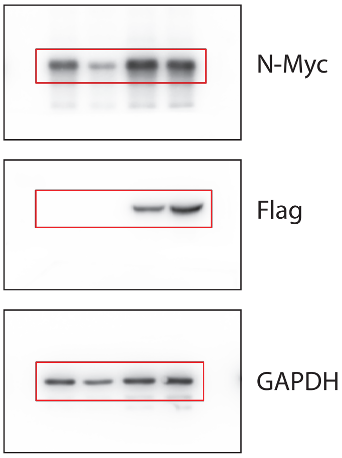

Figure 3G

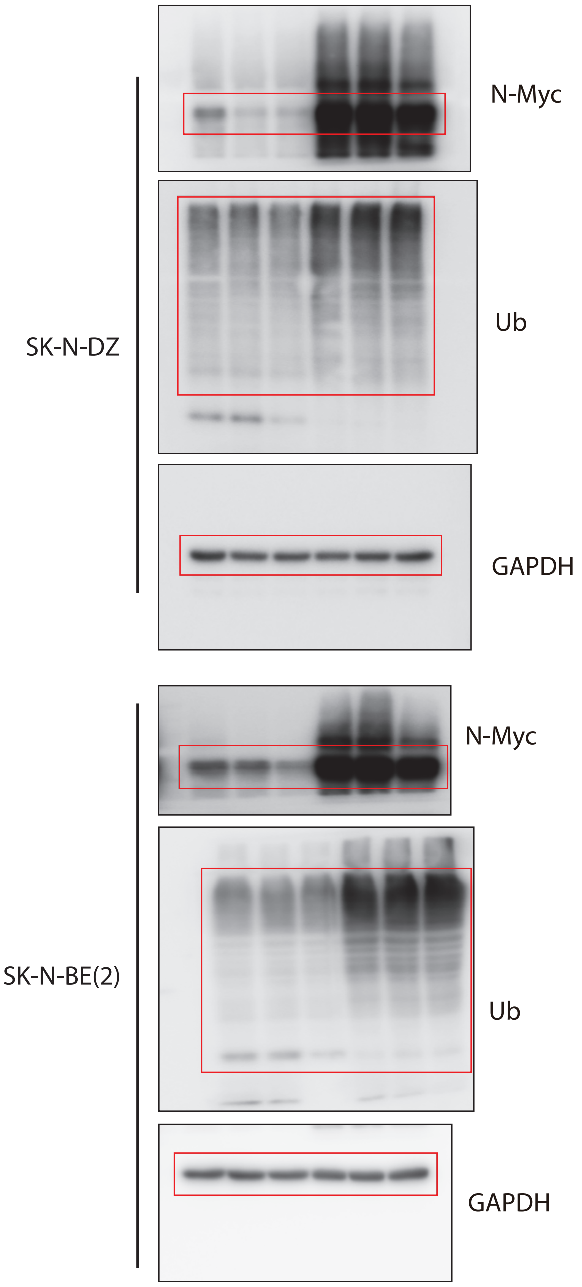

Figure 3H

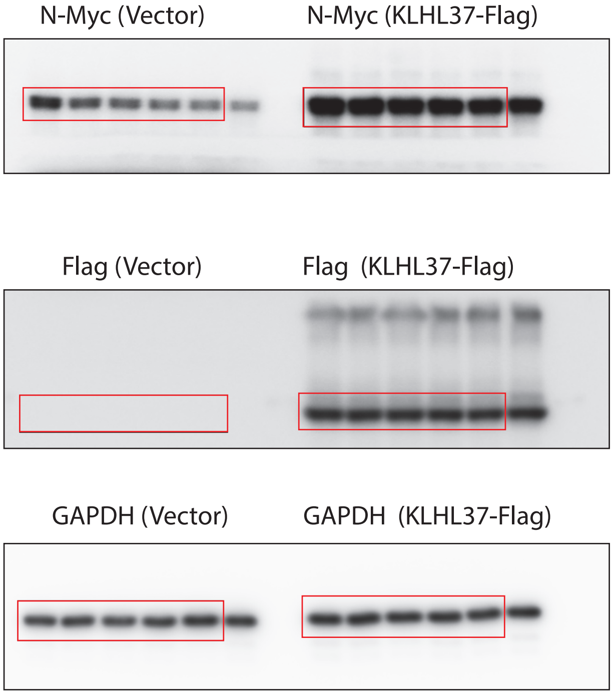
N-Myc (Vector)
N-Myc (KLHL37-Flag)
Flag (Vector)
Flag (KLHL37-Flag)
GAPDH (Vector)
GAPDH (KLHL37-Flag)

Figure 3I

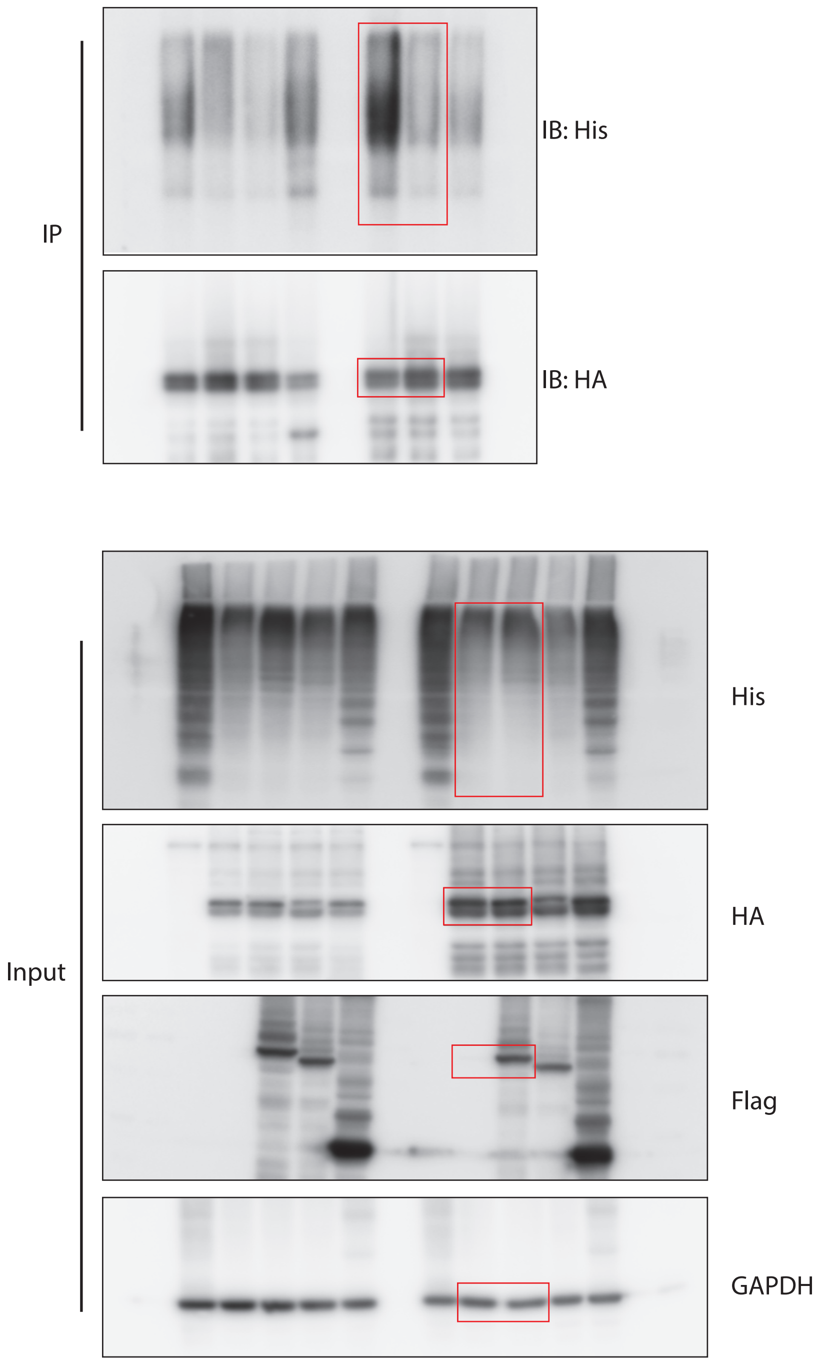

Figure 3J

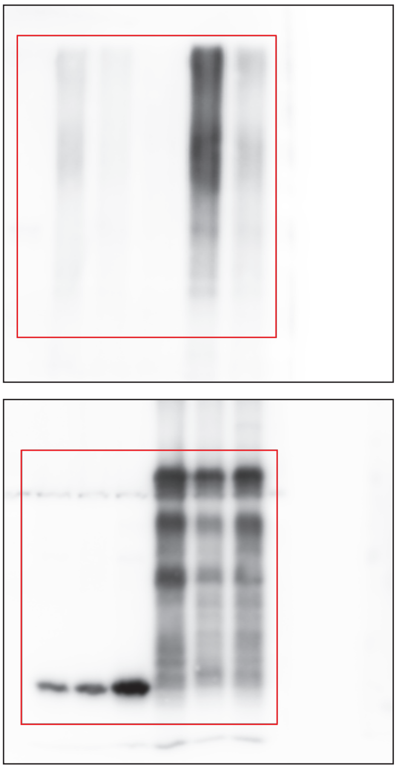

Figure 3K

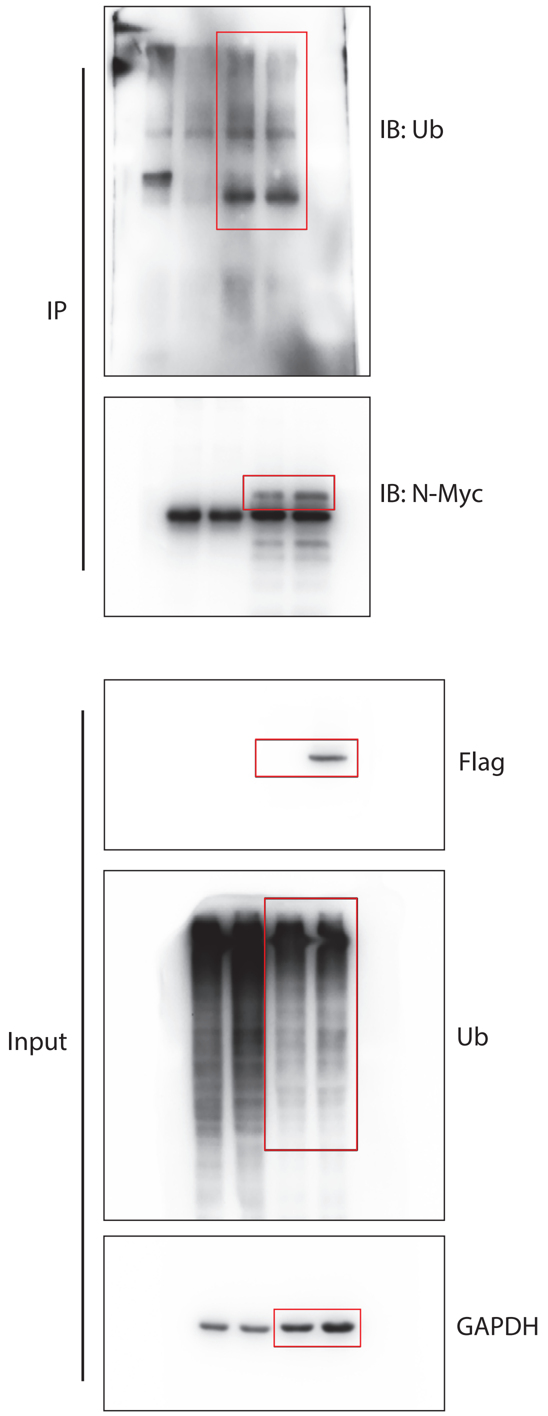

Figure 4A

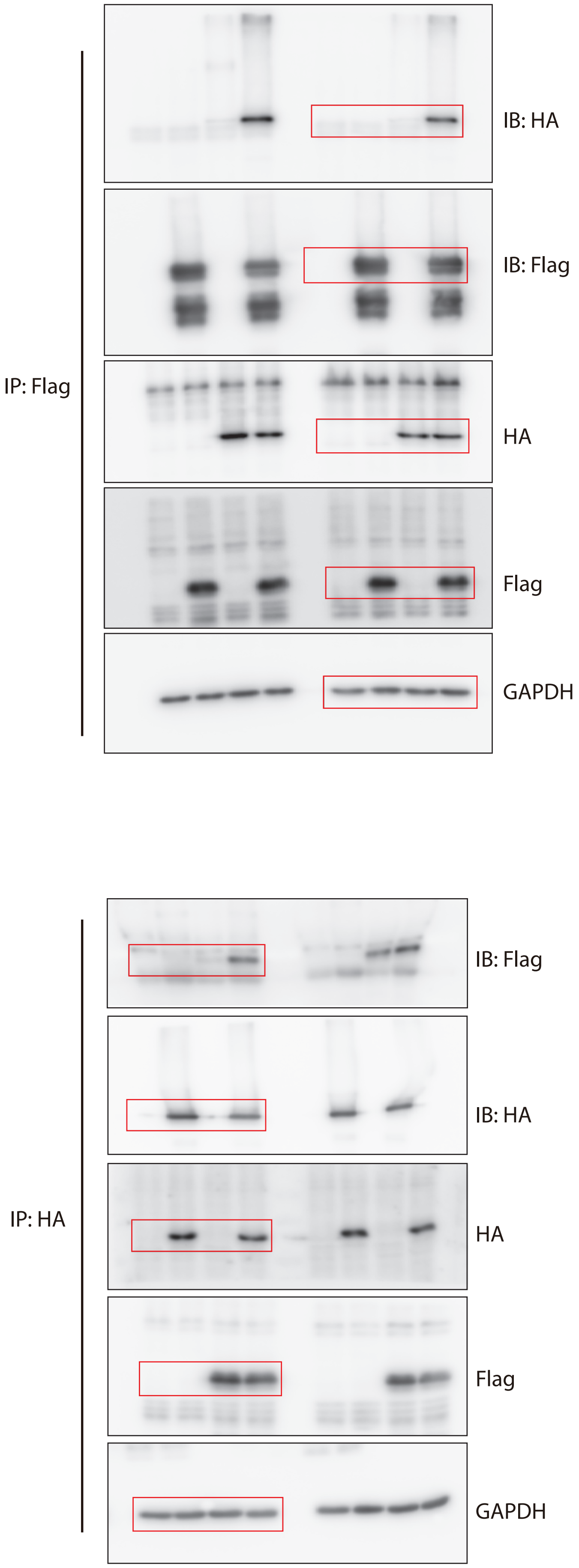
IB: HA
IB: Flag
IP: Flag
HA
Flag
GAPDH
IB: Flag
IB: HA
IP: HA
HA
Flag
GAPDH

Figure 4B

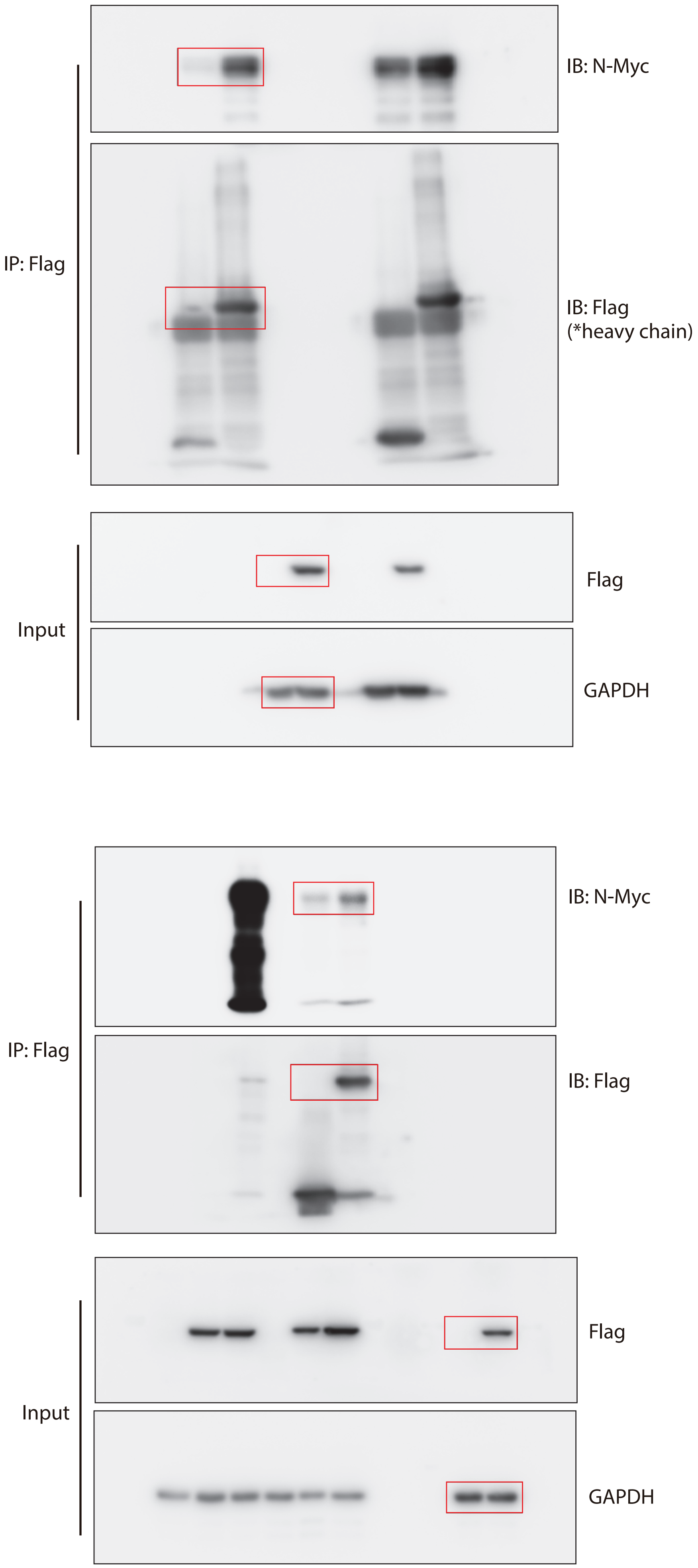

IB: N-Myc
IP: Flag
IB: Flag
(*heavy chain)
Flag
Input
GAPDH
IB: N-Myc
IP: Flag
IB: Flag
Flag
Input
GAPDH

Figure 4D

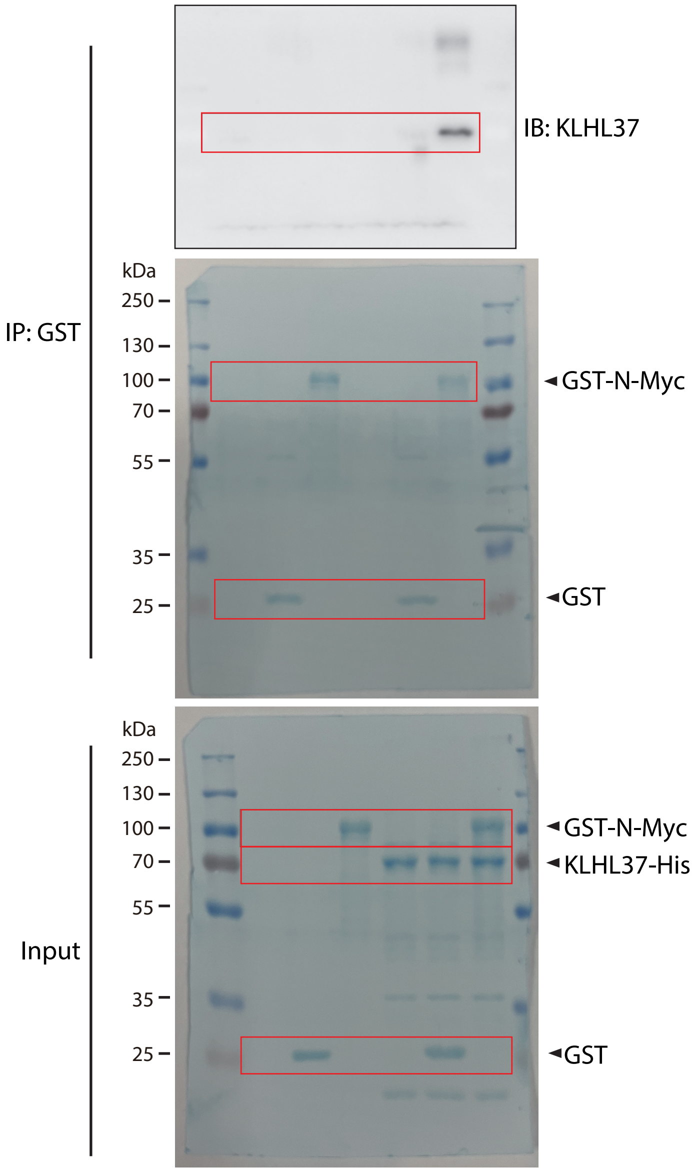
IB: KLHL37
IP: GST
kDa
250
130
100
70
55
35
25
GST-N-Myc
GST
Input
kDa
250
130
100
70
55
35
25
GST-N-Myc
KLHL37-His
GST

Figure 4F

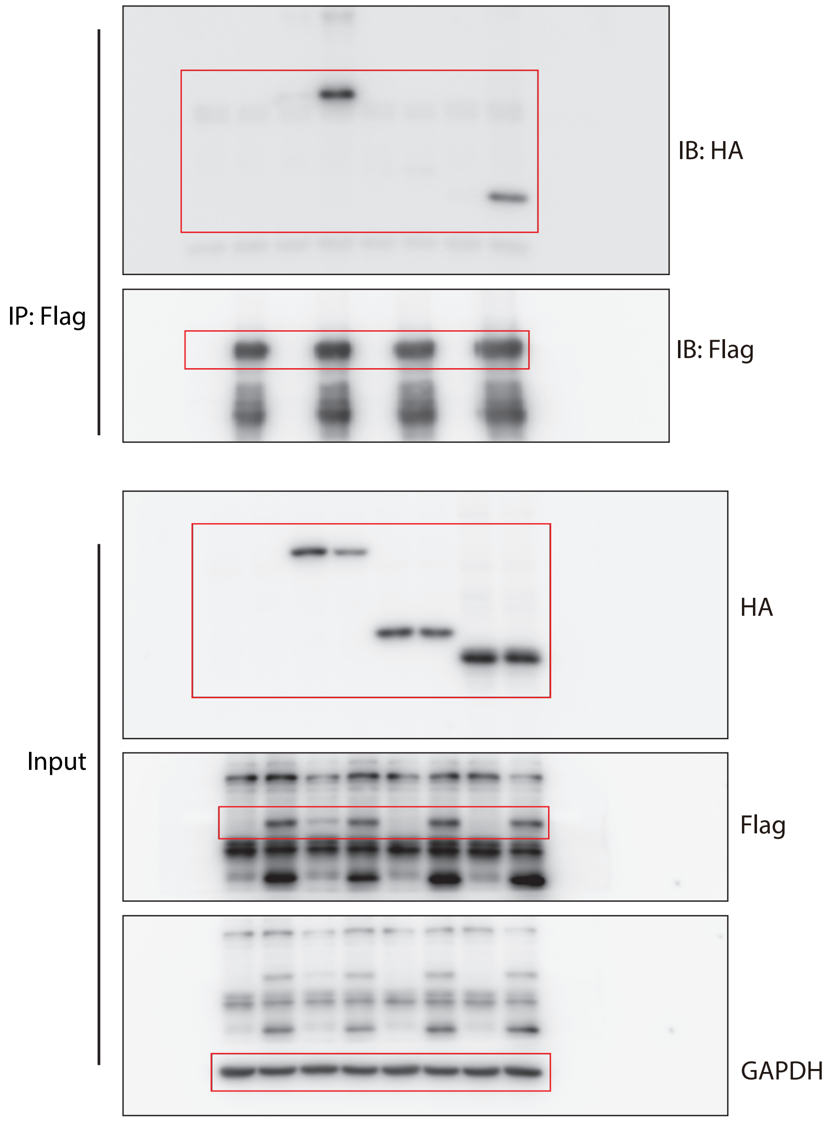

Figure 4G

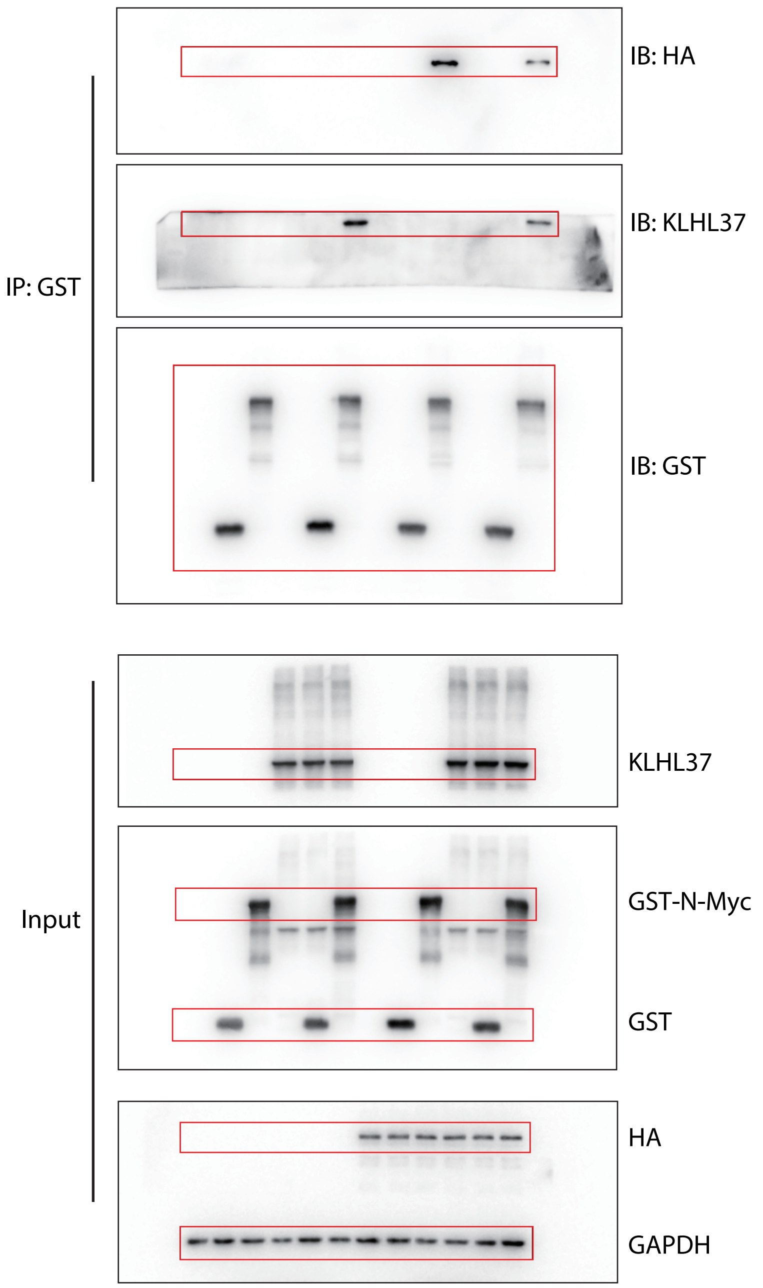
IB: HA
IB: KLHL37
IP: GST
IB: GST
KLHL37
GST-N-Myc
Input
GST
HA
GAPDH

Figure 4H

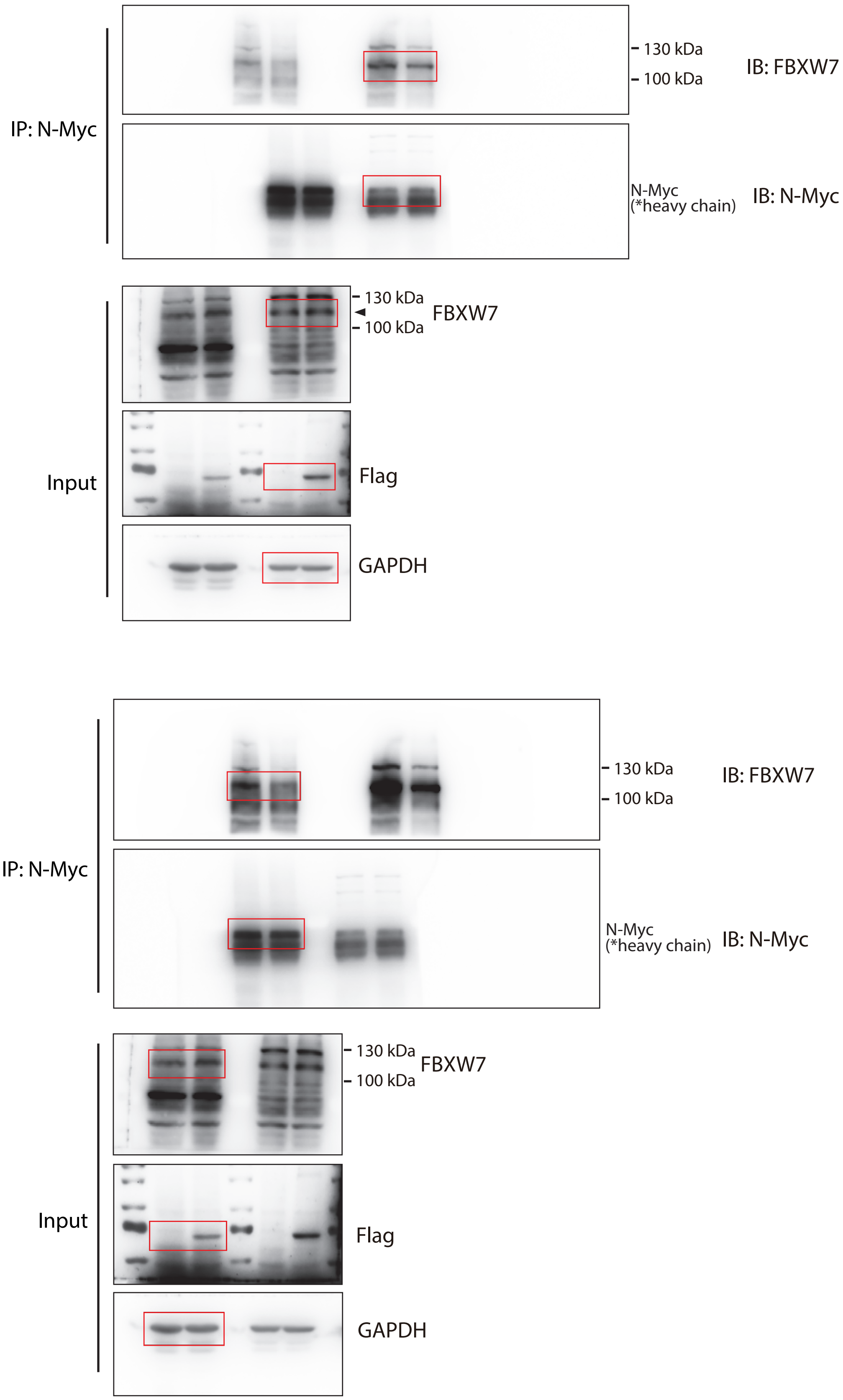
IP: N-Myc
130 kDa
100 kDa
IB: FBXW7
N-Myc
(*heavy chain)
IB: N-Myc
Input
130 kDa
100 kDa
FBXW7
Flag
GAPDH
IP: N-Myc
130 kDa
100 kDa
IB: FBXW7
N-Myc
(*heavy chain)
IB: N-Myc
Input
130 kDa
100 kDa
FBXW7
Flag
GAPDH

Figure 4I

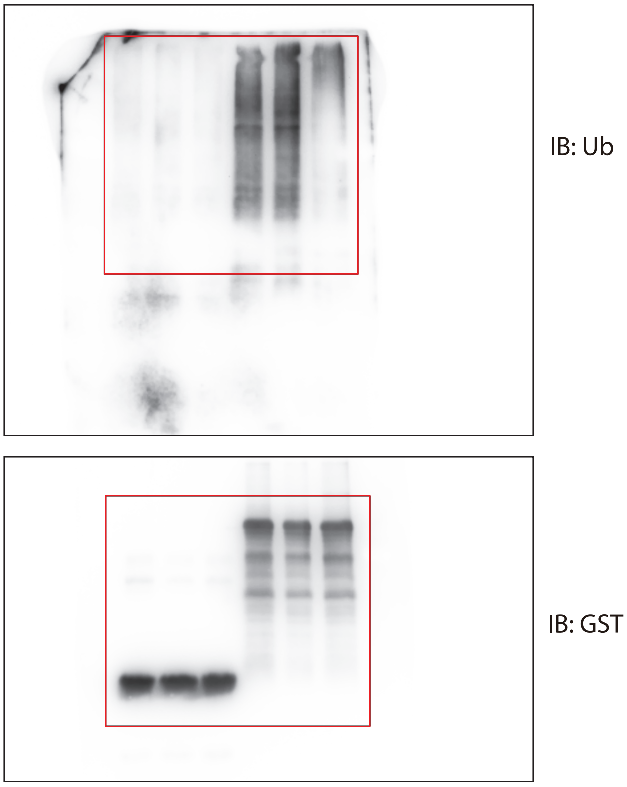

Figure 4J

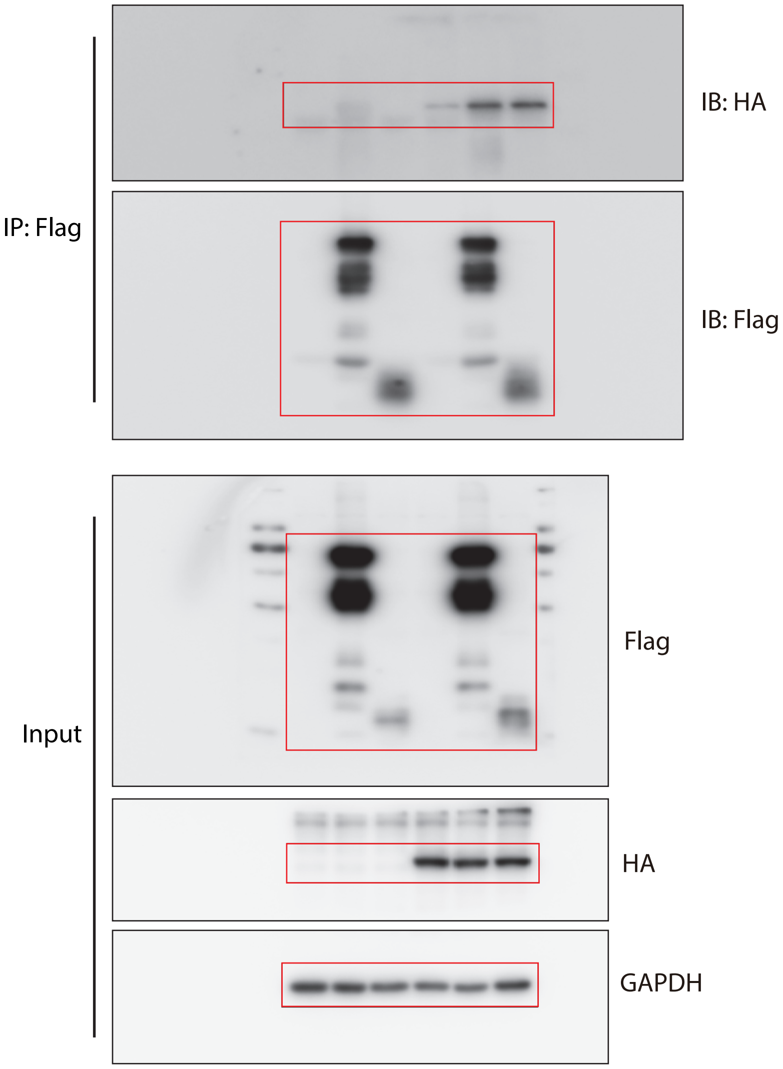

Figure 5C

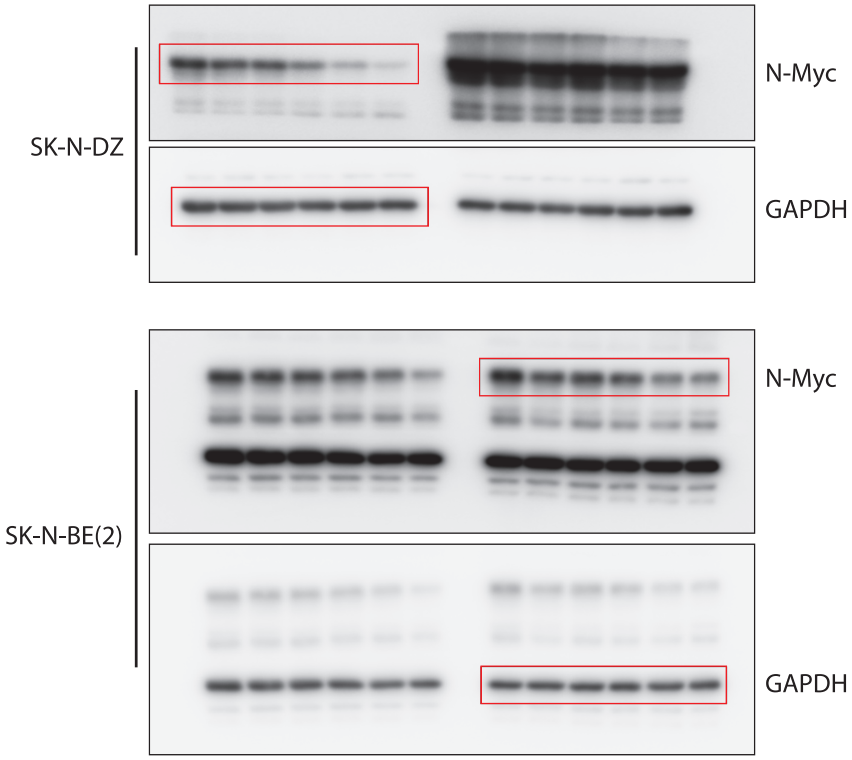

Figure 5D

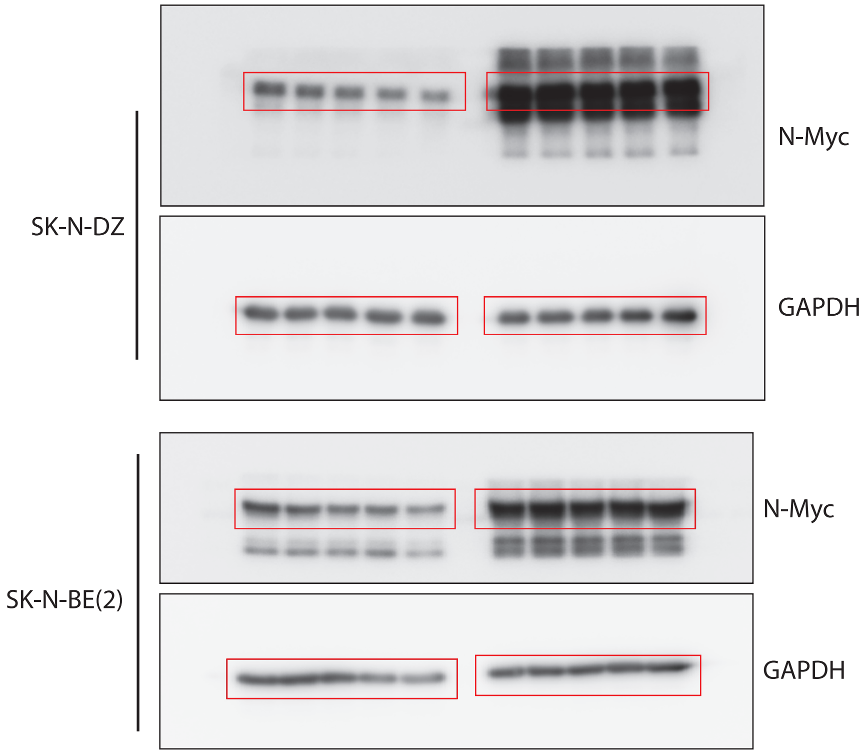

Figure 5H

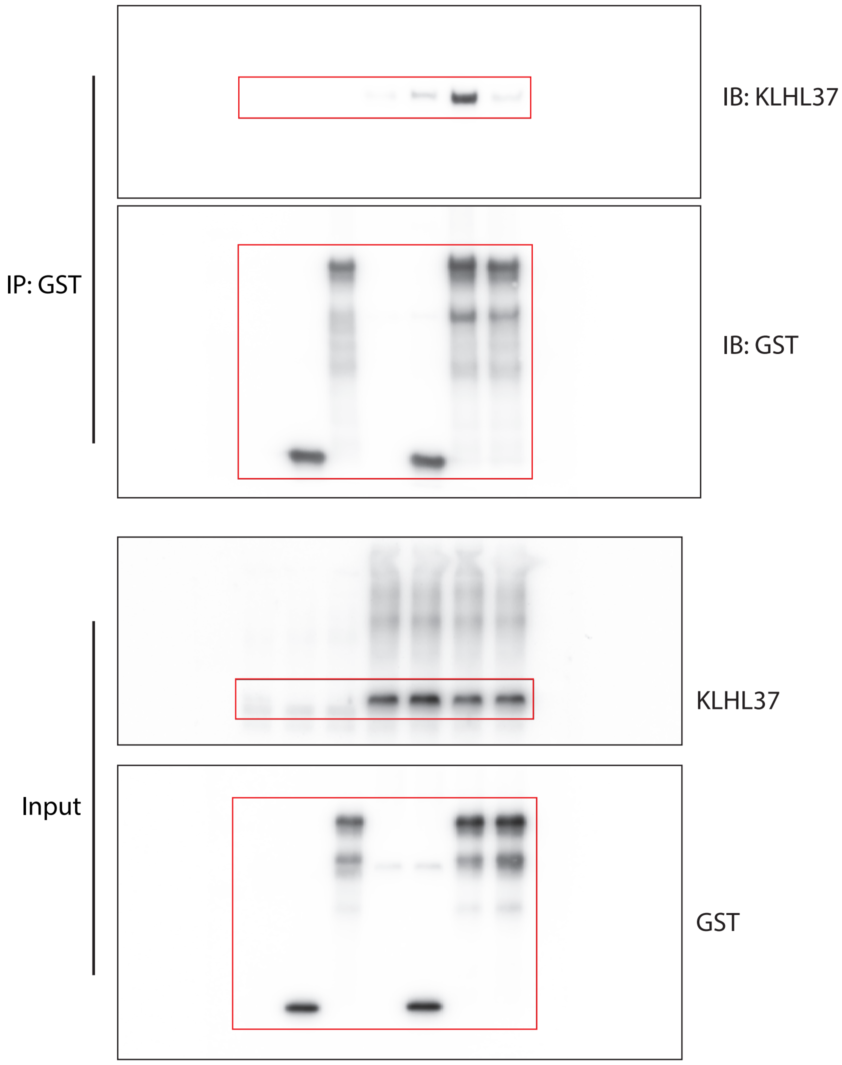

Figure 5l

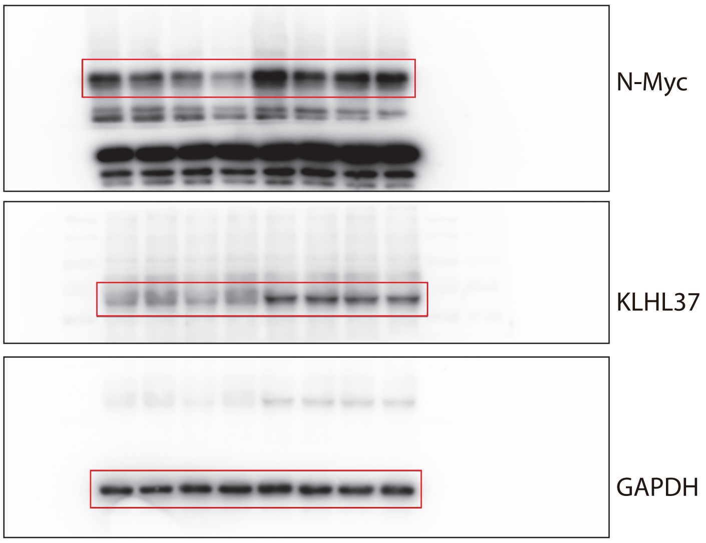

Figure 6H

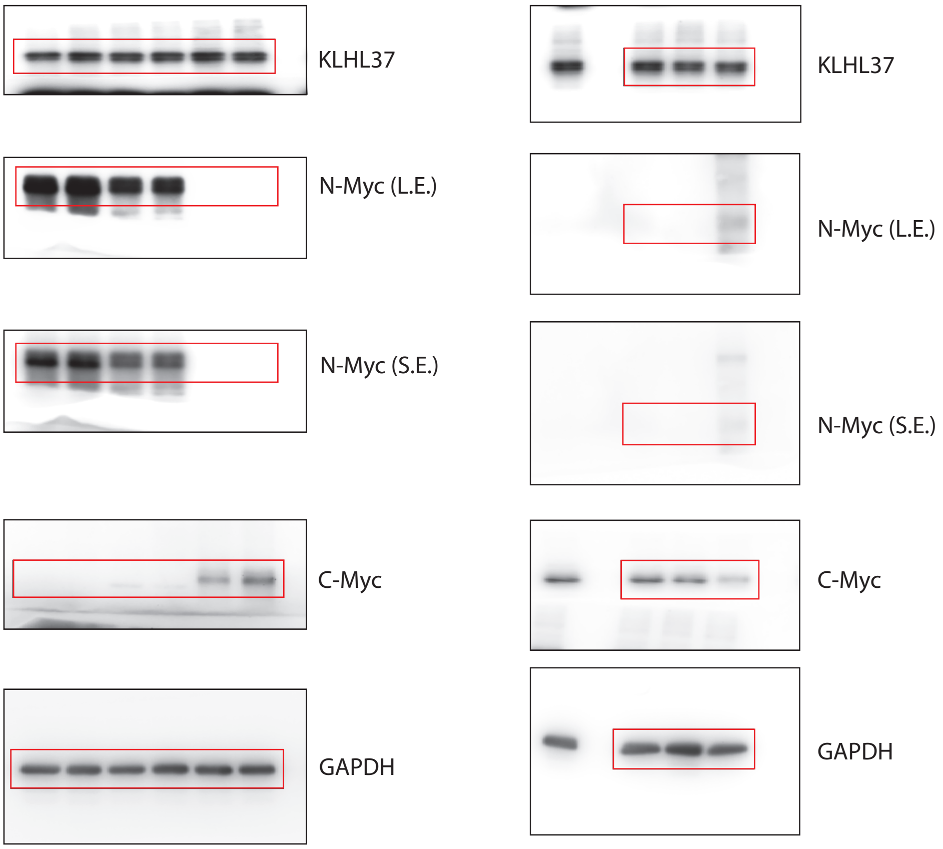

KLHL37
N-Myc (L.E.)
N-Myc (S.E.)
C-Myc
GAPDH
KLHL37
N-Myc (L.E.)
N-Myc (S.E.)
C-Myc
GAPDH

Figure 7D

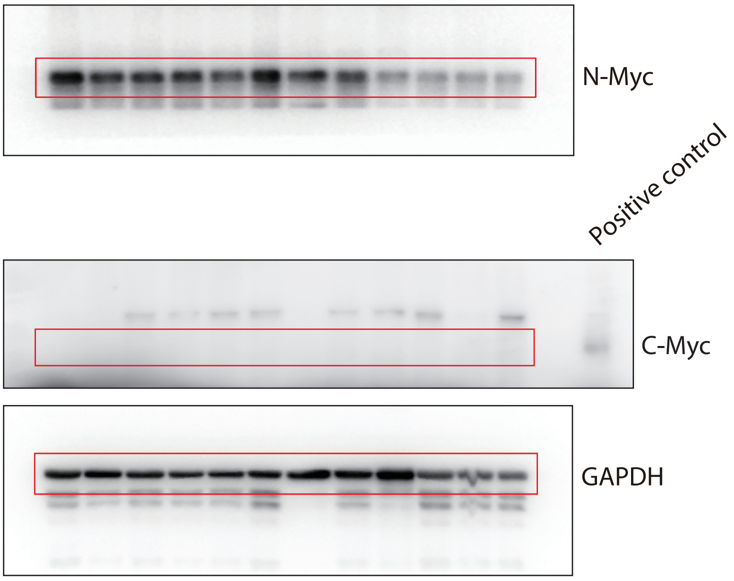

Figure S1A

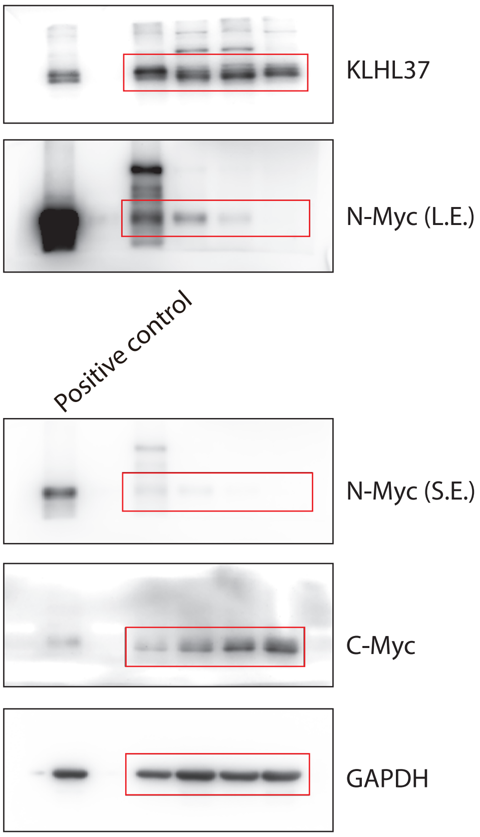

Figure S2B

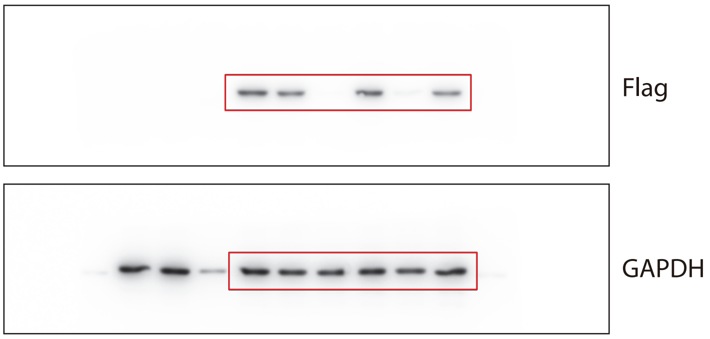

Figure S4A

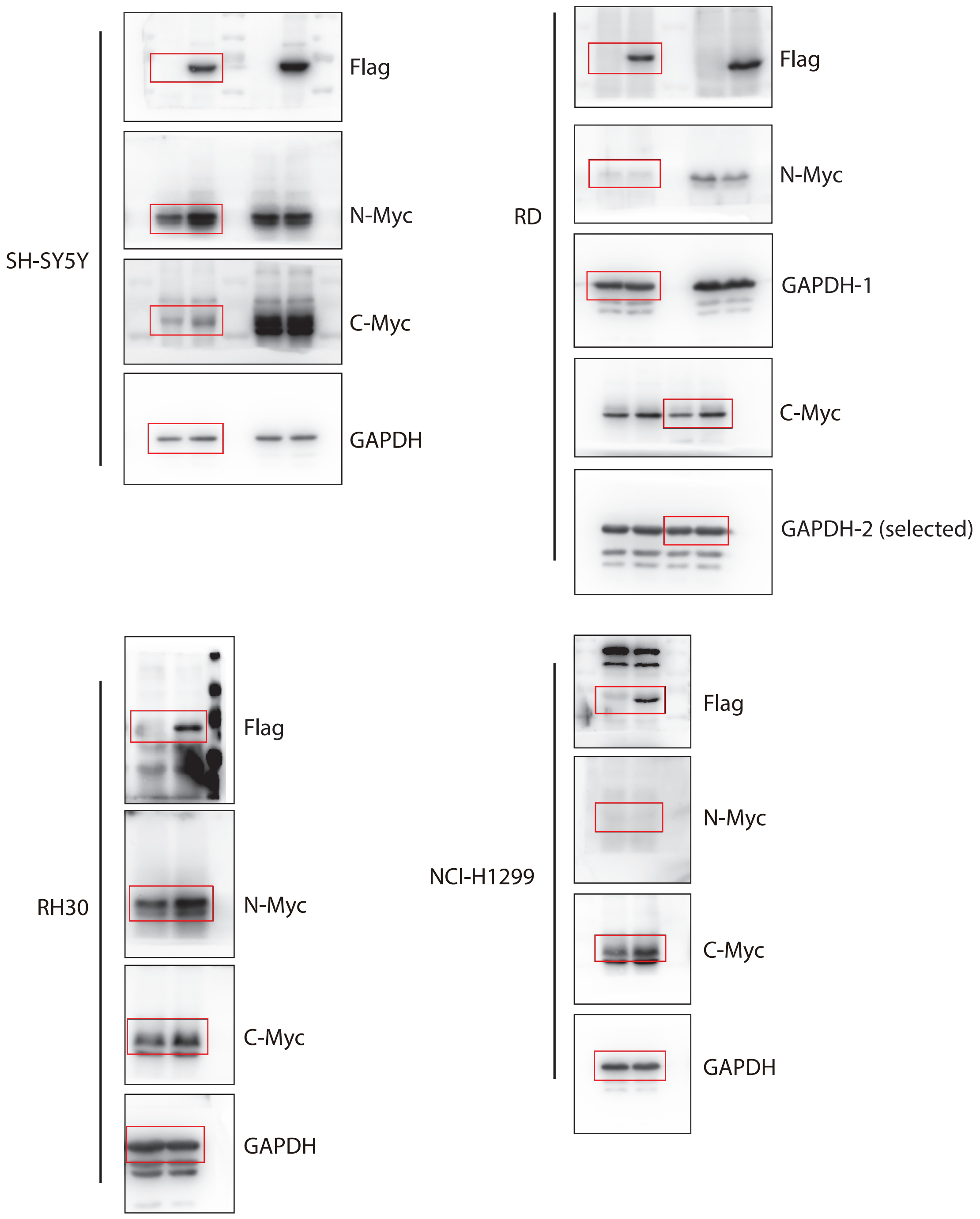
SH-SY5Y
Flag
N-Myc
C-Myc
GAPDH
RD
Flag
N-Myc
GAPDH-1
C-Myc
GAPDH-2 (selected)
RH30
Flag
N-Myc
C-Myc
GAPDH
NCI-H1299
Flag
N-Myc
C-Myc
GAPDH

Figure S4B

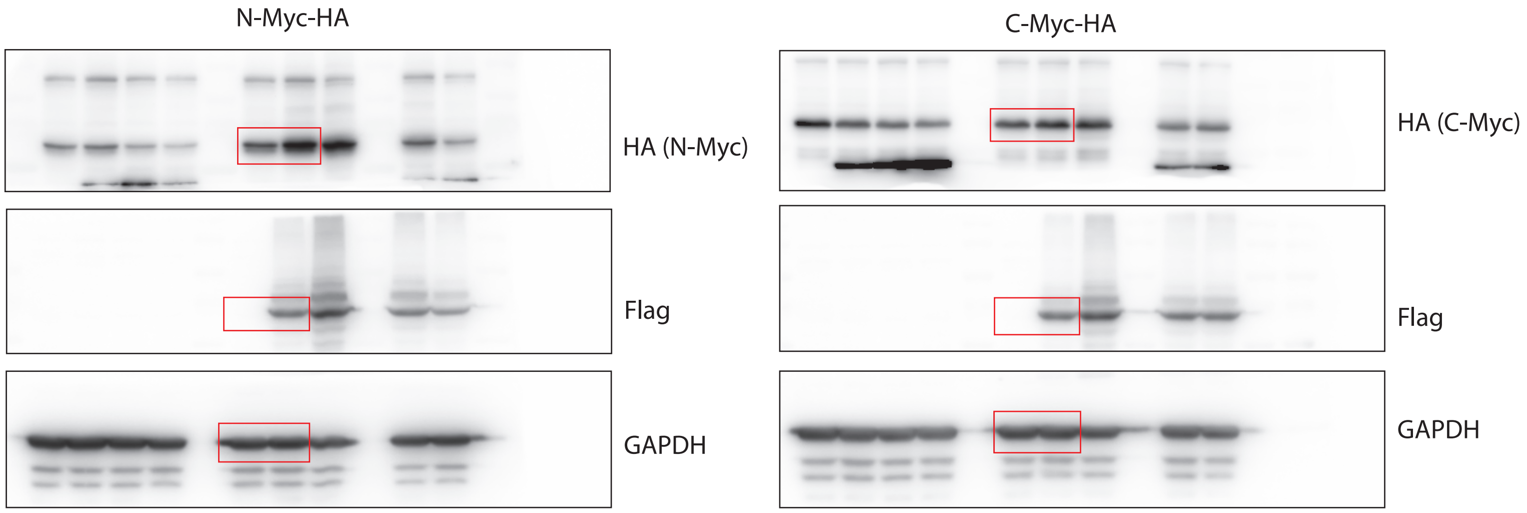

Figure S4C

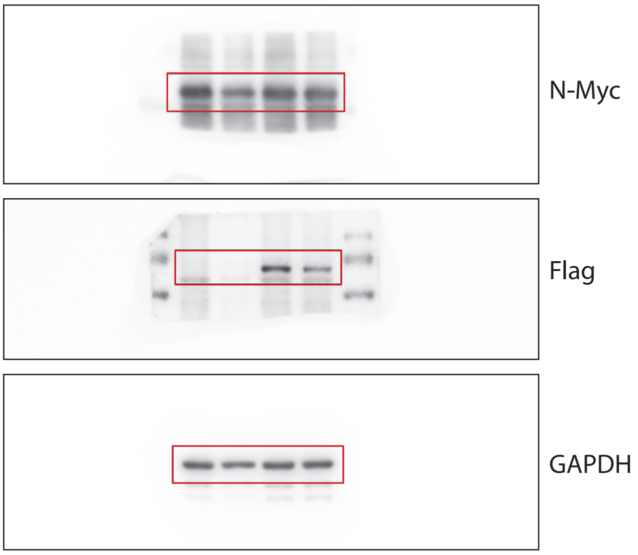

Figure S5A

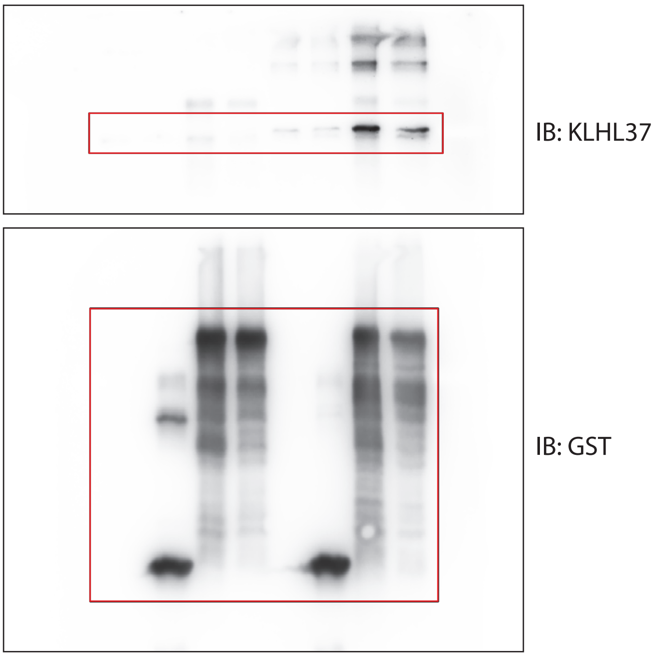

Figure S5C

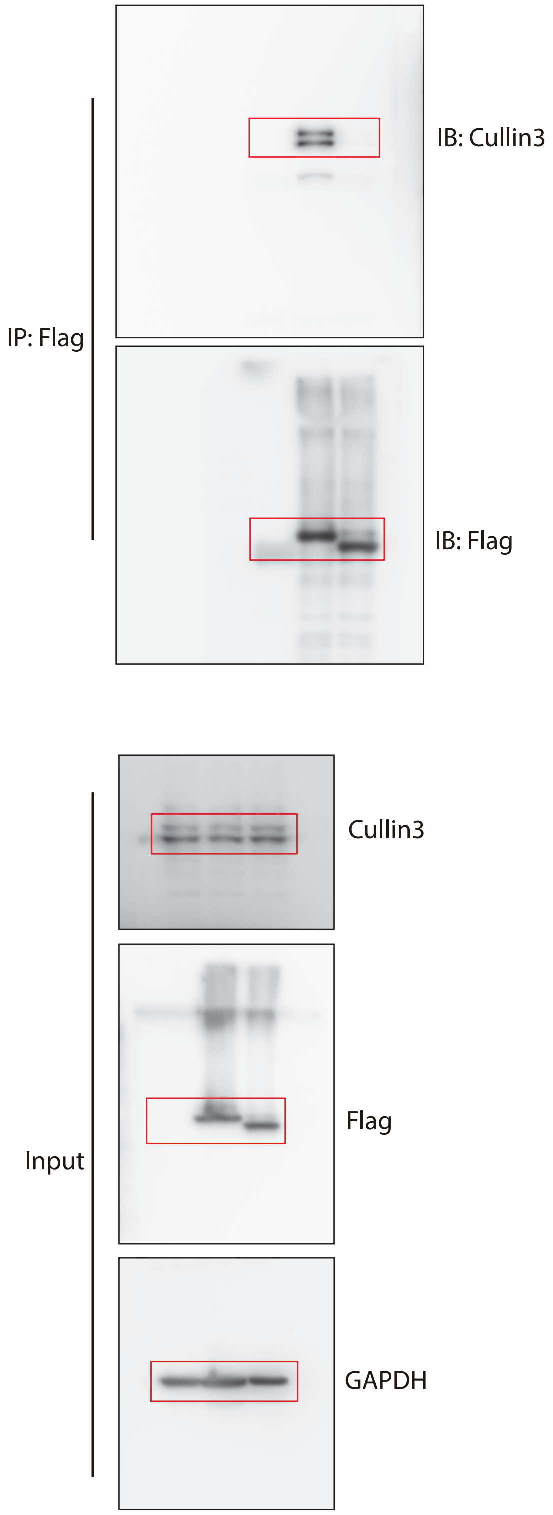

Figure S5D

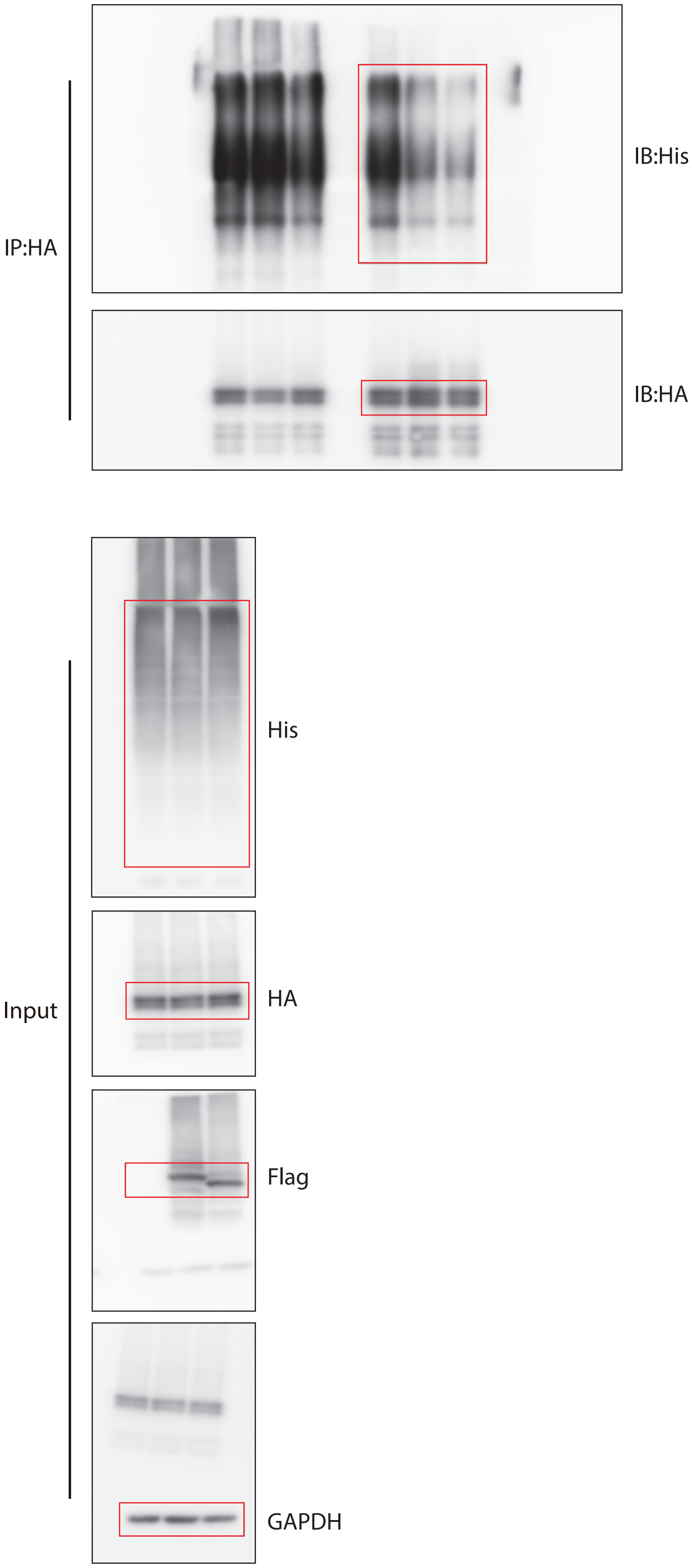

Figure S5E

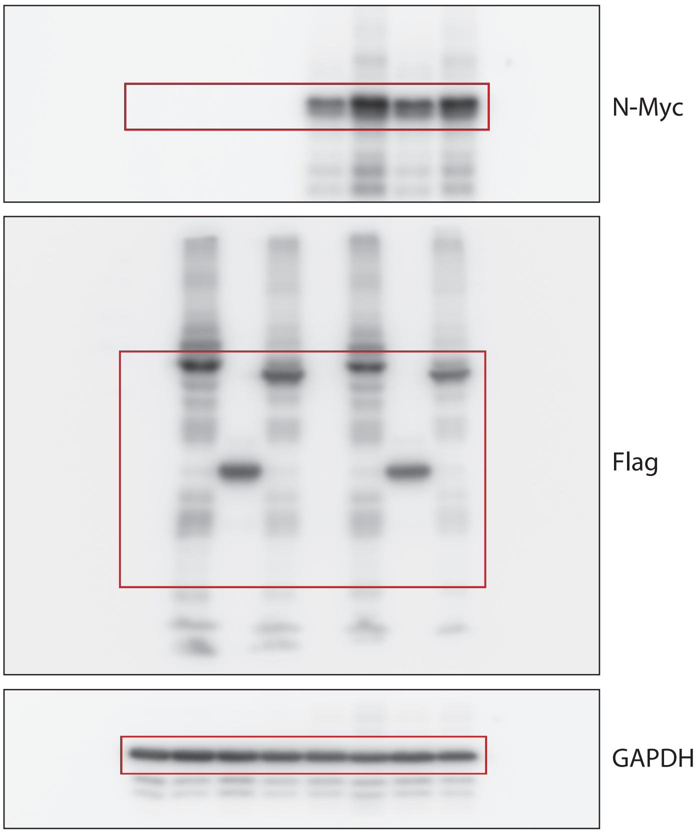

Figure S6A

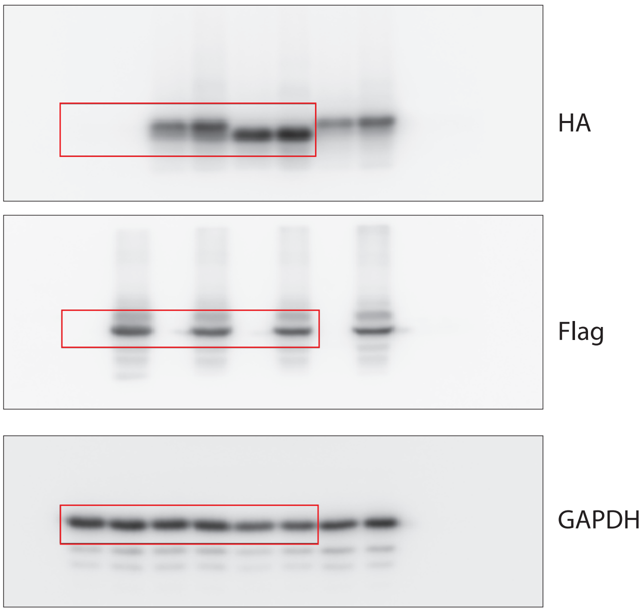

Figure S6B

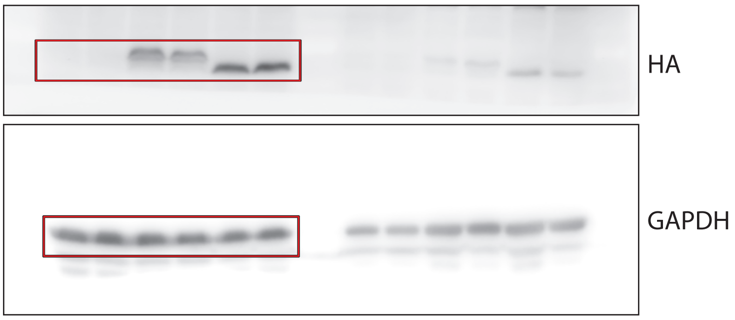

Figure S6C

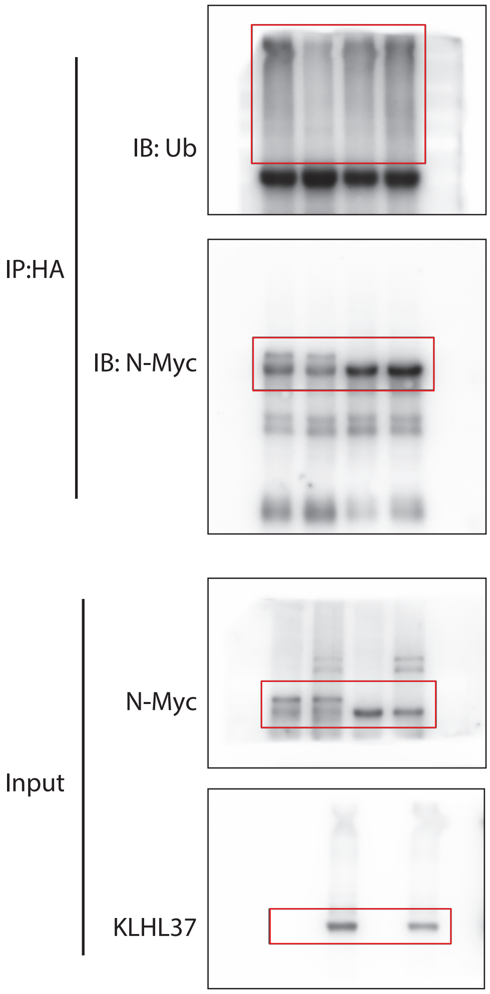

Figure S6D

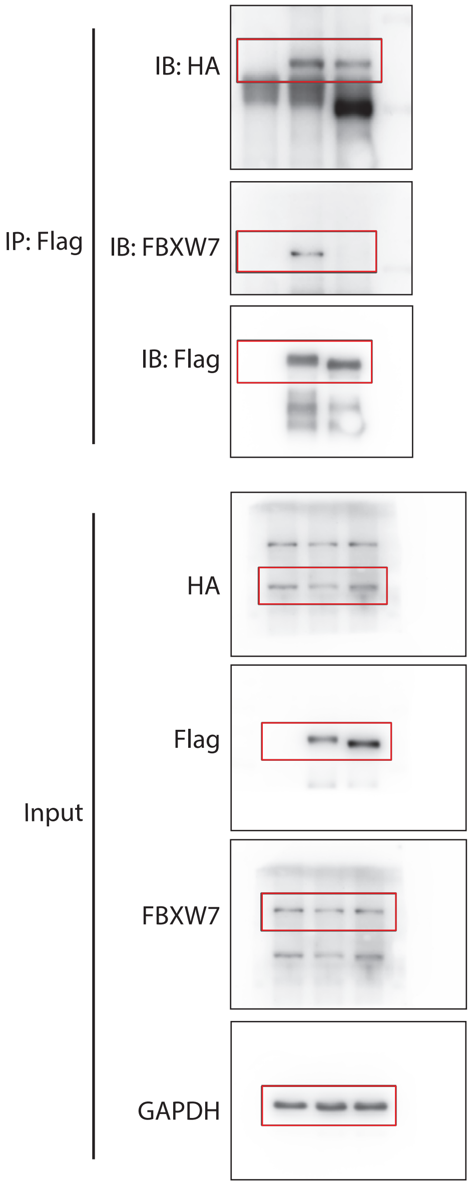

Figure S6E

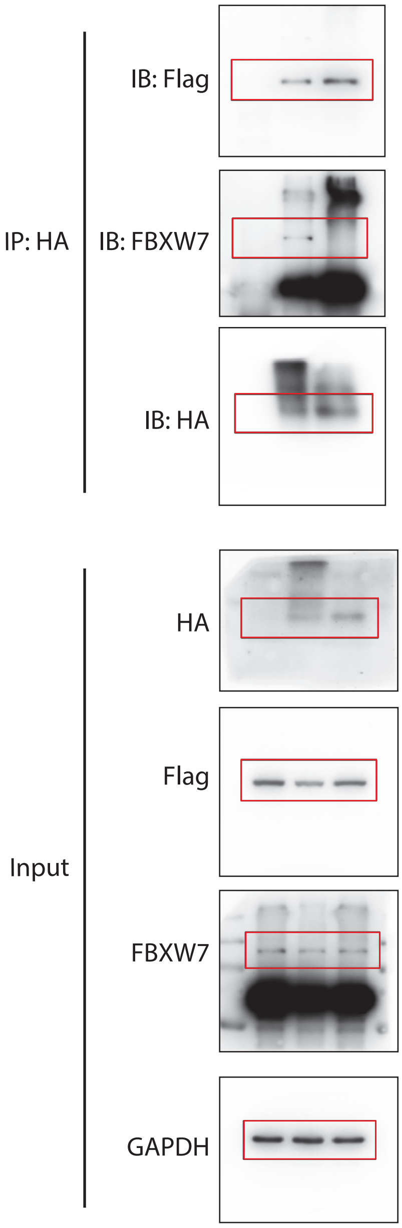

Figure S7C

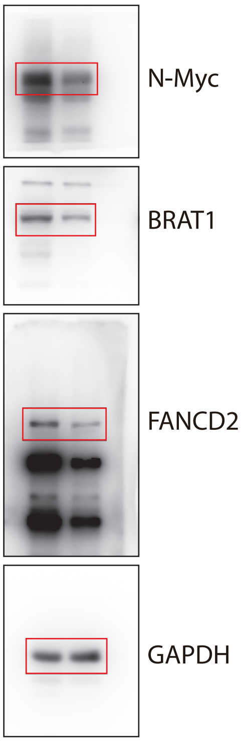

Figure S8A

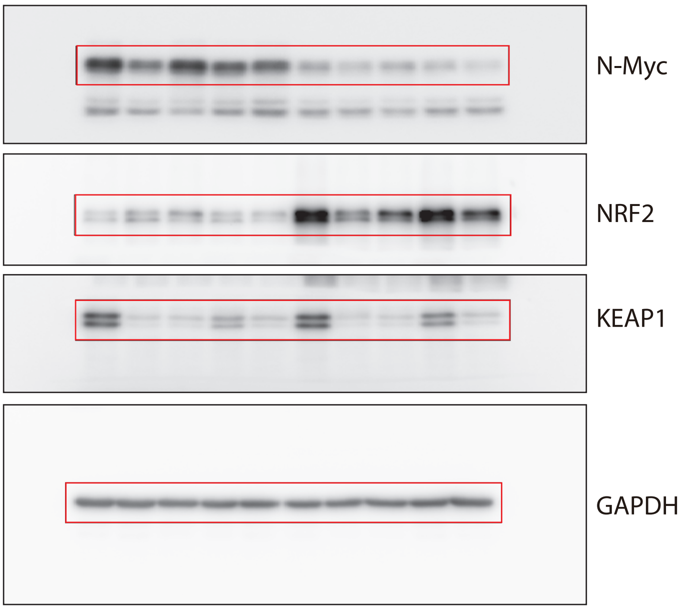

Figure S8B

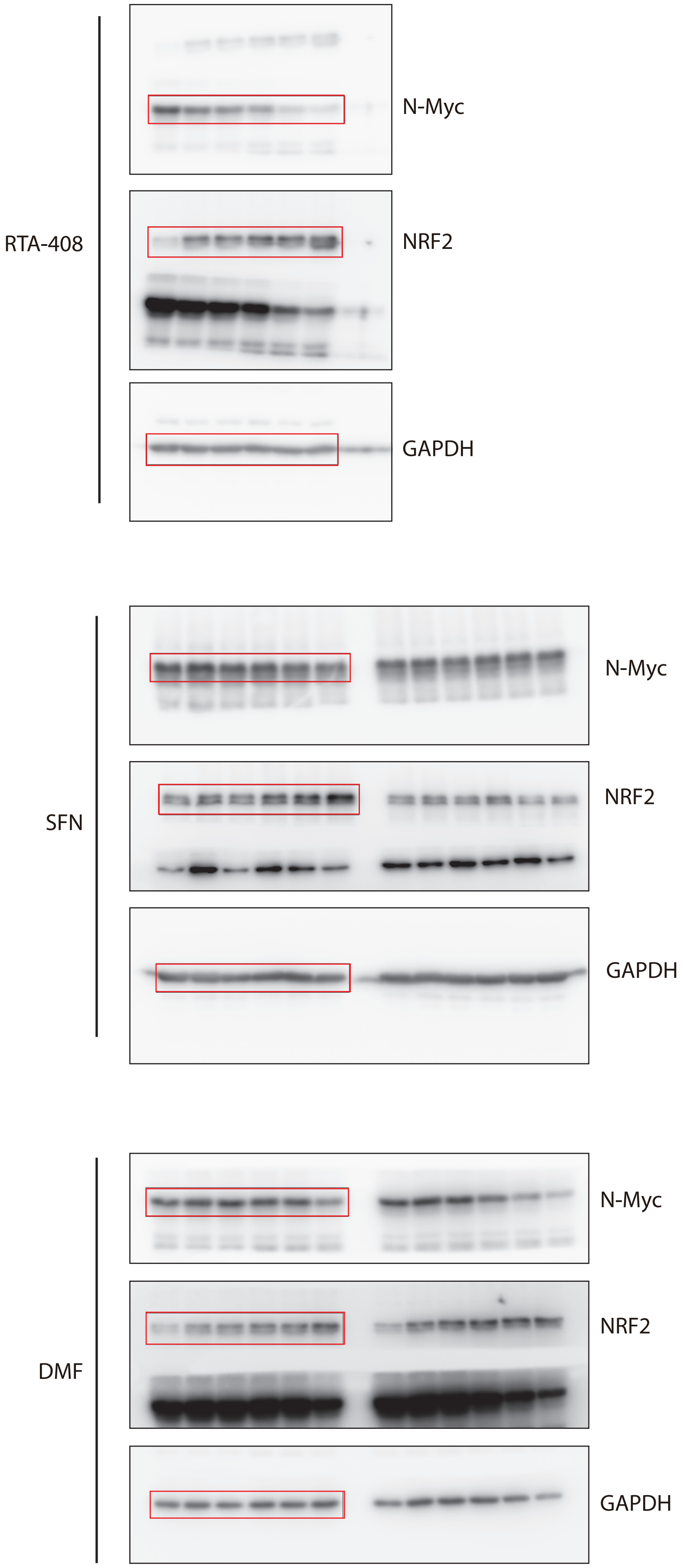
RTA-408
N-Myc
NRF2
GAPDH
SFN
N-Myc
NRF2
GAPDH
DMF
N-Myc
NRF2
GAPDH

Figure S8C

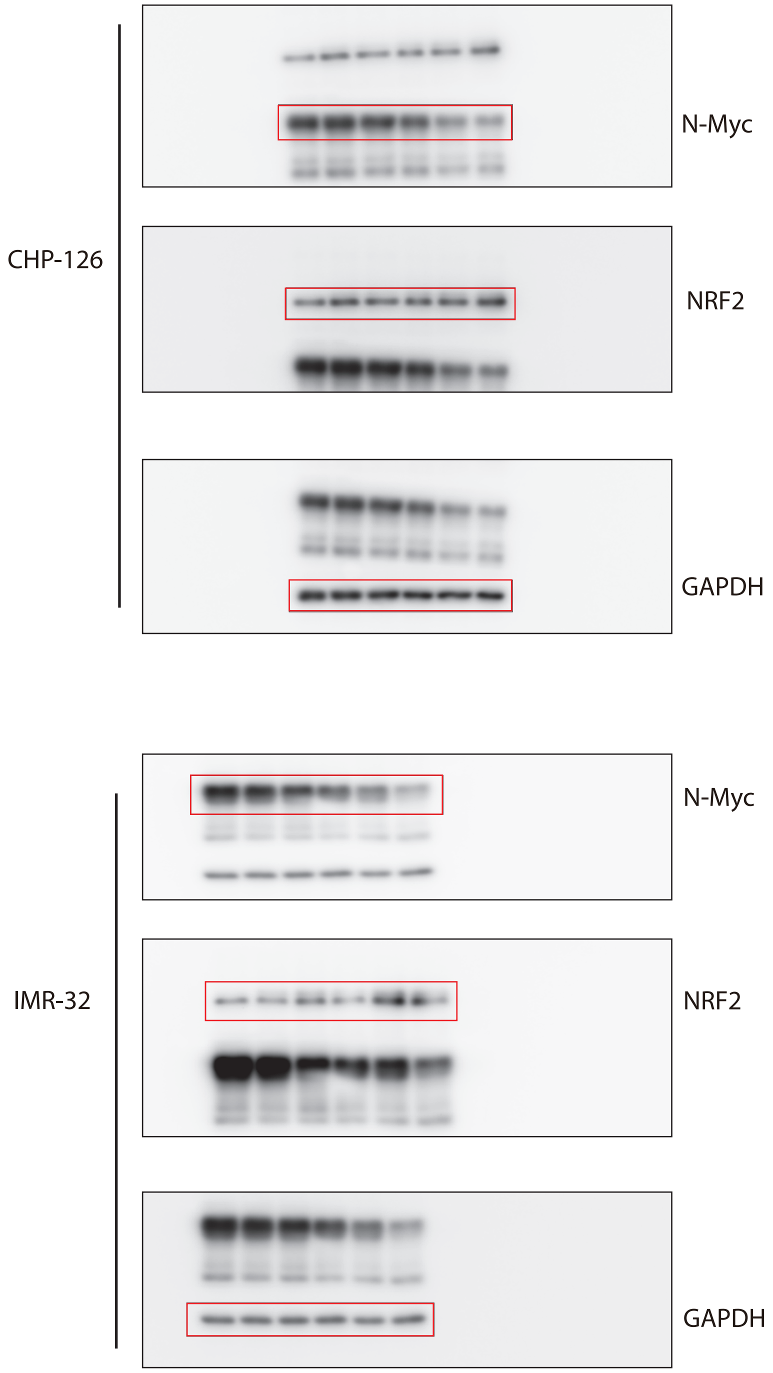

Figure S8D

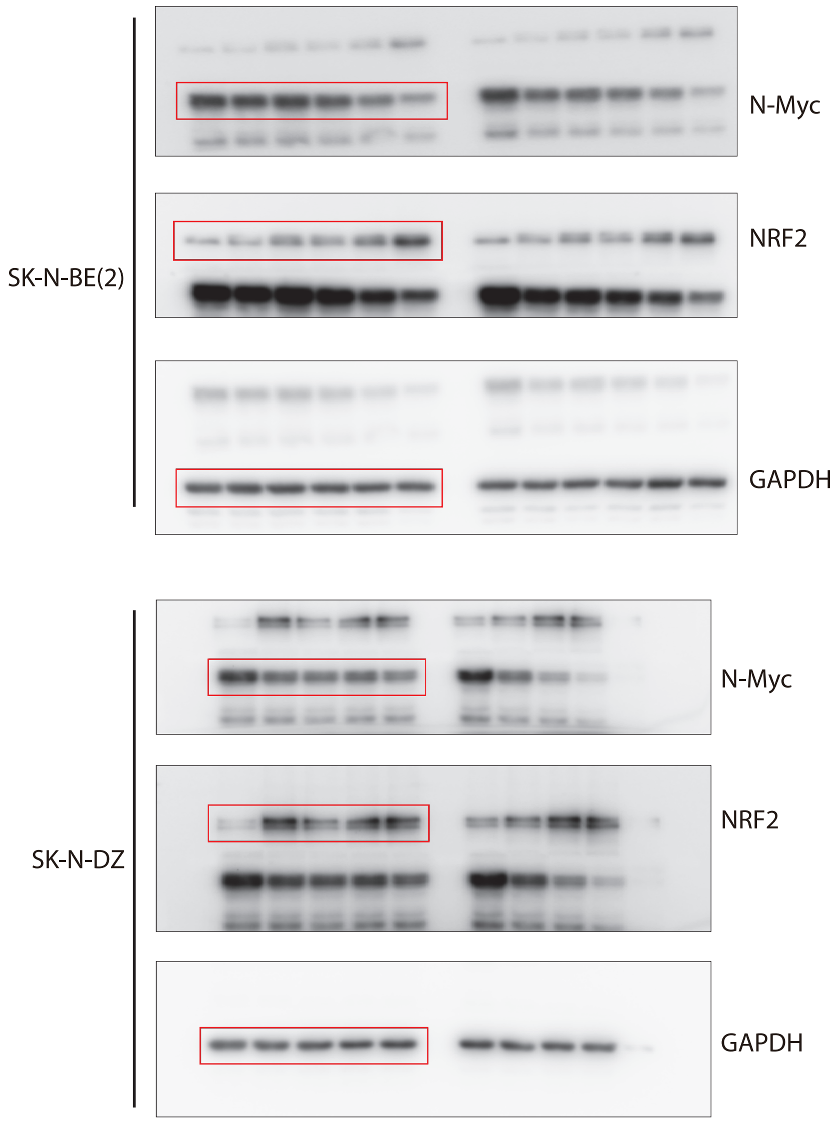

Figure S8E

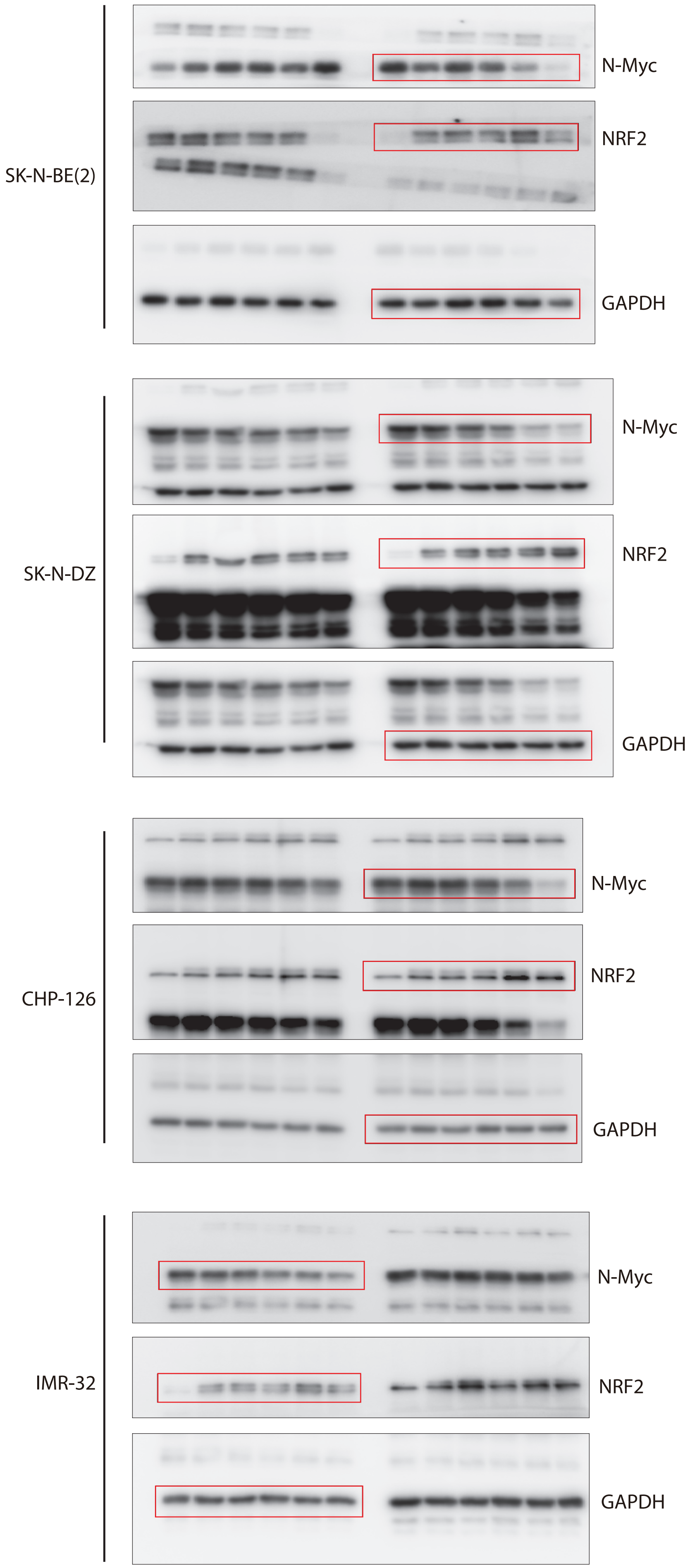
SK-N-BE(2)
N-Myc
NRF2
GAPDH
SK-N-DZ
N-Myc
NRF2
GAPDH
CHP-126
N-Myc
NRF2
GAPDH
IMR-32
N-Myc
NRF2
GAPDH

Figure S8H

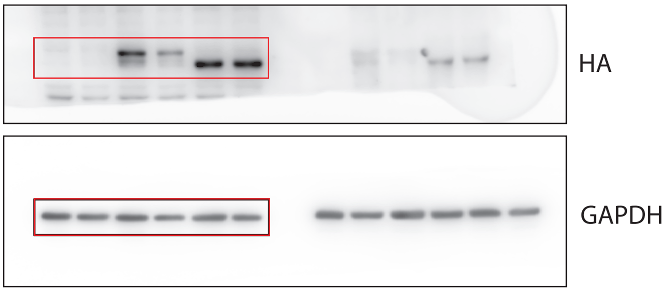

Figure S9E

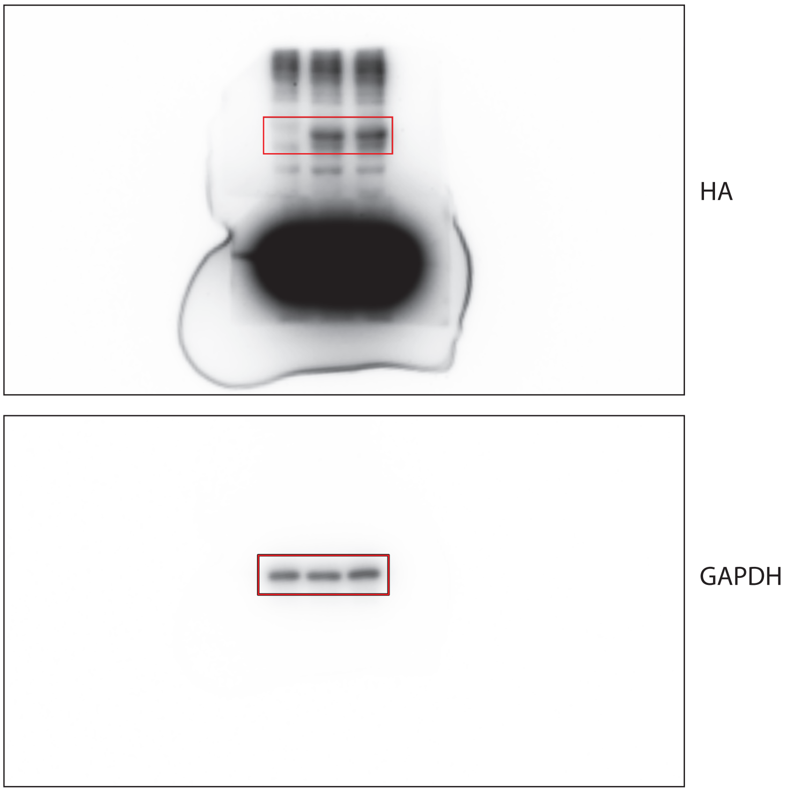

Figure S9F

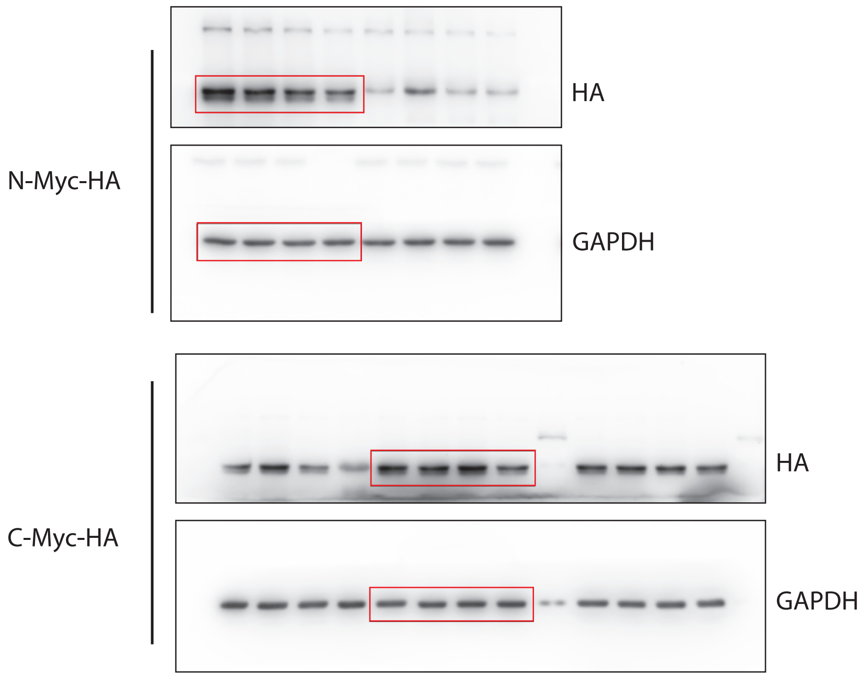

Figure S10A

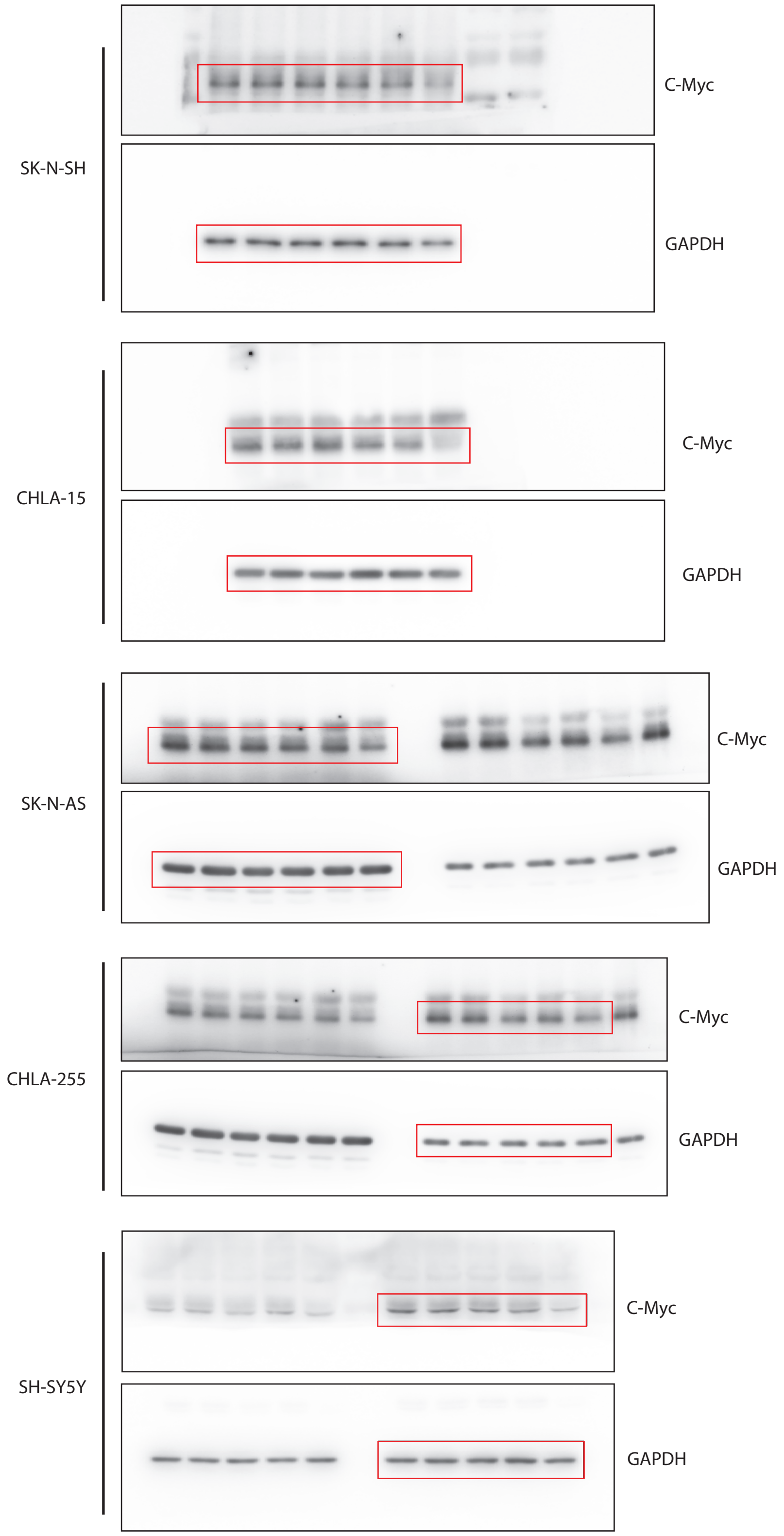
SK-N-SH
C-Myc
GAPDH
CHLA-15
C-Myc
GAPDH
SK-N-AS
C-Myc
GAPDH
CHLA-255
C-Myc
GAPDH
SH-SY5Y
C-Myc
GAPDH

Figure S11E

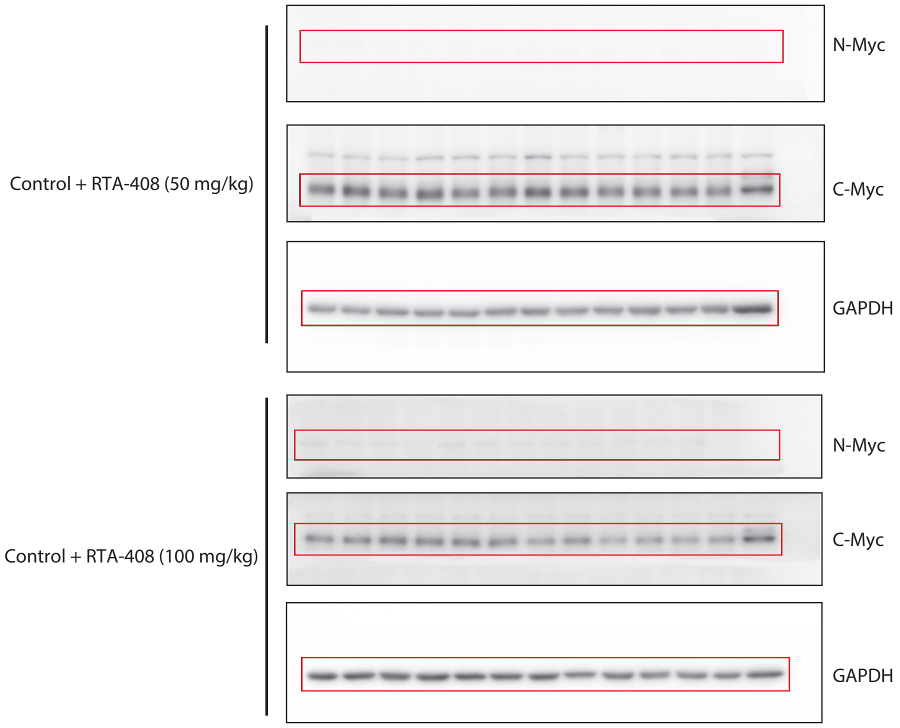
N-Myc
C-Myc
GAPDH
Control + RTA-408 (50 mg/kg)
N-Myc
C-Myc
GAPDH
Control + RTA-408 (100 mg/kg)
